# A Mixed-Valence Ti(II)/Ti(III) Inverted Sandwich Compound as a Regioselective Catalyst for the Uncommon 1,3,5-Alkyne Cyclotrimerization

**DOI:** 10.1021/acs.inorgchem.4c00149

**Published:** 2024-05-01

**Authors:** Elena Álvarez-Ruiz, Ignacio Sancho, Marta Navarro, Israel Fernández, Cristina Santamaría, Alberto Hernán-Gómez

**Affiliations:** † Departamento de Química Orgánica y Química Inorgánica, Instituto de Investigación Química “Andrés M. del Río” (IQAR), Universidad de Alcalá, Campus Universitario, Alcalá de Henares, Madrid E-28805, Spain; ‡ Departamento de Química Inorgánica, Orgánica y Bioquímica, Facultad de Ciencias y Tecnologías Químicas, Universidad de Castilla-La Mancha, Ciudad Real 13071, Spain; § Departamento de Química Orgánica I, Facultad de Ciencias Químicas and Centro de Innovación en Química Avanzada (ORFEO−CINQA), Universidad Complutense de Madrid, Madrid 28040, Spain

## Abstract

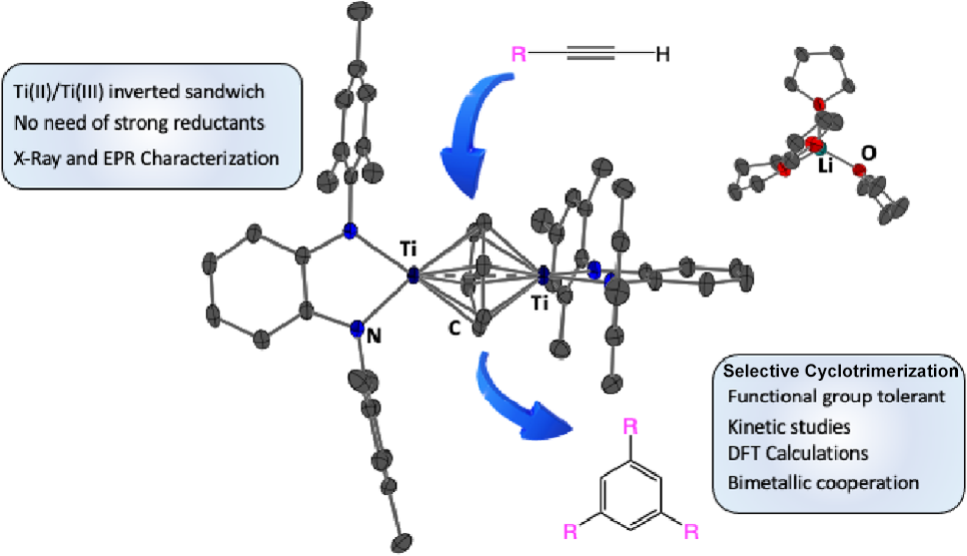

The synthesis, structure, and catalytic activity of a Ti(II)/Ti(III) inverted sandwich compound are presented in this study. Synthesis of the arene-bridged dititanium compound begins with the preparation of the titanium(IV) precursor [TiCl_2_(^Mes^PDA)(thf)_2_] (^Mes^PDA = *N*,*N*′-bis(2,4,6-trimethylphenyl)-*o*-phenylenediamide) (**2**). The reduction of **2** with sodium metal results in species [{Ti(^Mes^PDA)(thf)}_2_(μ-Cl)_3_{Na}] (**3**) in oxidation state III. To achieve the lower oxidation state II, **2** undergoes reduction through alkylation with lithium cyclopentyl. This alkylation approach triggers a cascade of reactions, including β-hydride abstraction/elimination, hydrogen evolution, and chemical reduction, to generate the Ti(II)/Ti(III) compound [Li(thf)_4_][(Ti^Mes^PDA)_2_(μ–η^6^: η^6^-C_6_H_6_)] (**4**). X-ray and EPR characterization confirms the mixed-valence states of the titanium species. Compound **4** catalyzes a mild, efficient, and regiospecific cyclotrimerization of alkynes to form 1,3,5-substituted arenes. Kinetic data support a mechanism involving a binuclear titanium arene compound, similar to compound **4**, as the resting state. The active catalyst promotes the oxidative coupling of two alkynes in the rate-limiting step, followed by a rapid [4 + 2] cycloaddition to form the arene product. Computational analysis of the resting state for the cycloaddition of trimethylsilylacetylene indicates a thermodynamic preference for stabilizing the 1,3,5-arene within the space between the two [Ti^Mes^PDA] fragments, consistent with the observed regioselectivity.

## Abstract 2

In this study, we introduce a low-valent Ti(II)/Ti(III) inverted sandwich compound, characterized through X-ray and EPR spectroscopy. Insights into the formation mechanism are obtained by DFT studies, NMR spectroscopy, and pressure reaction monitoring. This compound effectively catalyzes the regioselective cyclotrimerization of alkynes, showing good functional group tolerance and remarkable regioselectivity for 1,3,5-arenes achieved through bimetallic mediation.

## Introduction

**Figure 1:**
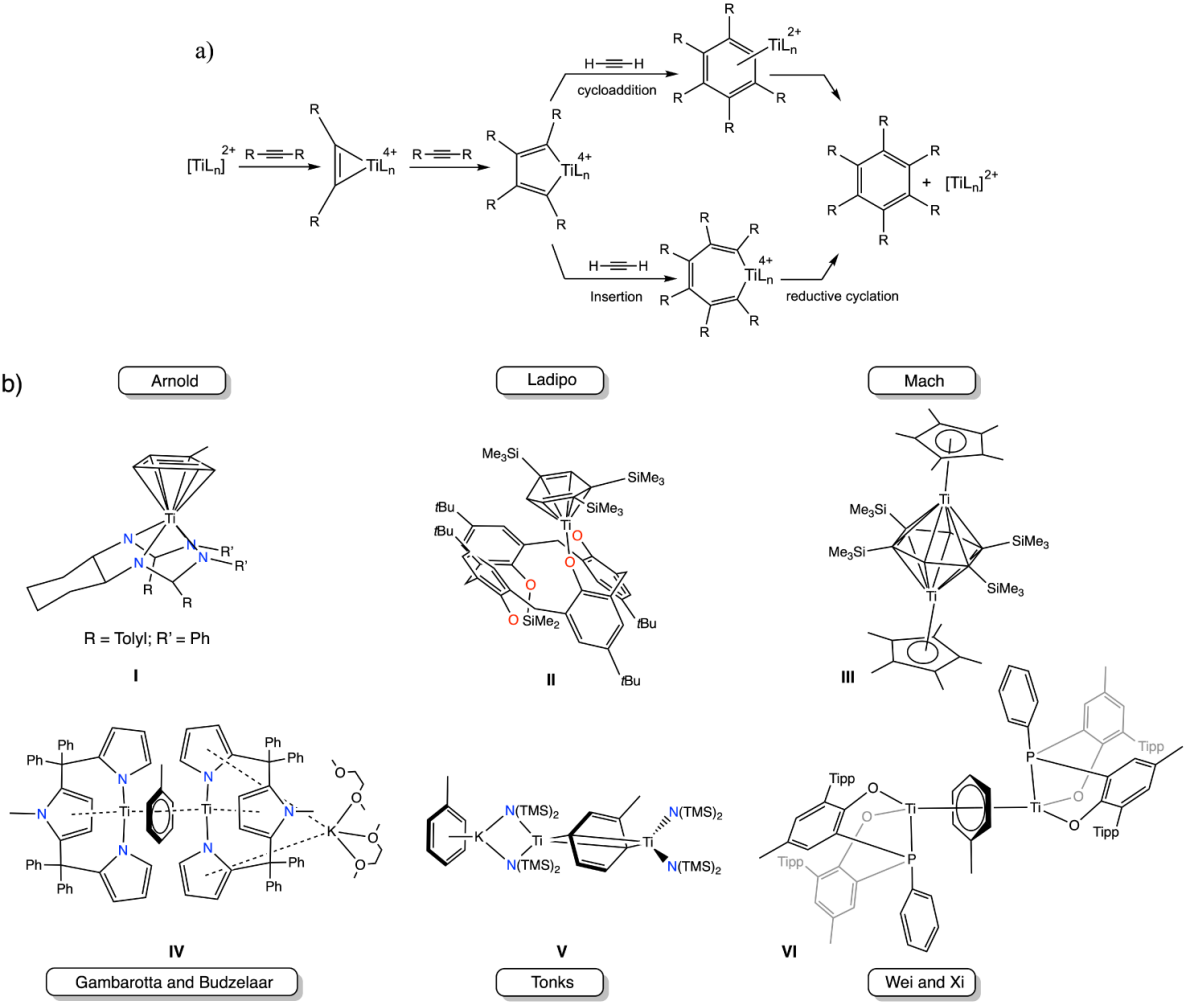
a) Mechanism for the cyclotrimerization of alkynes. b) Reported low-valent titanium–arene compounds.

The isolation of inorganic species invoked as intermediates in catalytic transformations is crucial for both understanding the fundamentals and advancing the catalytic process. In this context, low-valent titanium compounds have become versatile tools capable of mediating a multitude of chemical transformations.^1^ Among them, low-valent titanium–arene or (hetero)arene complexes are considered intermediate species in the cycloaddition reactions of unsaturated organic substrates catalyzed by titanium.^2^ These processes have received particular attention since they provide access to arenes or heteroarenes in one step, using an abundant and nonexpensive transition metal with low toxicity.^1b,3^ More specifically, the [2 + 2 + 1] synthesis of pyrroles from alkynes and azobenzene catalyzed by titanium compounds has progressed rapidly through substantial research efforts from the group of Tonks.^4^ Comparatively, the [2 + 2+ 2] cycloaddition of alkynes to form trisubstituted arene compounds mediated by titanium species has received less attention in recent times. Mechanistically,^5^ the cyclotrimerization of alkynes is initiated by the reaction of a Ti(II) species with an alkyne to form a metallacyclopropene compound (Figure 1a). Then, the latter intermediate evolves to a metallacyclopentadiene complex upon reaction with 1 equiv of alkyne. From this point, two pathways unfold: a [4 + 2] cycloaddition leads to a Ti–arene compound and eventually a benzene derivative, while an alternative insertion process forms metallacycloheptatriene before a reductive cyclization occurs. Interestingly, the mechanism points out that the catalytic cycle can be accessed by a titanium–arene compound. While a variety of structurally characterized titanium–arene compounds have been reported in the literature,^6^ their use in cyclotrimerization remains sparse.

Among the monometallic species, notable examples include the work of Arnold,^7^ who described the synthesis of the mononuclear compound **I** (Figure 1b) through hydrogenolysis of a titanium bisbenzyl amidinate. Later, Ladipo^8^ reported the formation of compound **II** using a calix[4]arene platform (Figure 1b). This compound was generated by the initial chemical reduction of the dichloride titanium precursor with magnesium, followed by the cyclotrimerization of trimethylsilylacetylene. For dinuclear titanium species, Mach^9^ described the isolation of species **III** (Figure 1b) bridged by an arene unit upon thermal treatment of the trisalkyl precursor [Cp*TiMe_3_] (Cp* = η^5^-C_5_Me_5_). Gambarrota and Budzelaar^10^ published the formation of a mixed-valence Ti(I)/Ti(II) toluene-bridged species by reacting a tripyrrole titanium dichloride compound with potassium metal (Figure 1b, **IV**). Through the reduction of the Ti(III) compound [Ti(N(TMS)_2_)_3_] (N(TMS)_2_ = bis(trimethylsilyl)amido) with the strong reductant KC_8_, Tonks^11^ has described the formation of the mixed-valence Ti(II)/Ti(III) dinuclear titanium compound **V** displayed in Figure 1b. More recently, Wei and Xi have reported the isolation of the arene-bridged dititanium complex **VI** upon reaction of the bulky 2,4,6-triisopropylbenzene-substituted bis(o-hydroxyphenyl)-phenylphosphine Ti(III) compound with a large excess of KC_8_.^12^ Despite the promising nature of these isolated species in cyclotrimerization reactions, it is notable that only Ladipo^8^ explored the catalytic potential of species **II** in the cycloaddition reaction of a selection of alkynes. This study stands out, offering one of the rare examples in which a titanium catalyst affords excellent yields and high regioselectivity for the formation of 1,2,4-trisubstituted benzene derivatives. In a more extended practice, to initiate the catalytic cycle, a ligand-supported Ti(IV) halide species is treated with a metallic reductant (Mg or Zn). This is illustrated by Okamoto in the [2 + 2 + 2] cycloaddition of alkynes catalyzed by the reaction mixture formed by CpTiX_3_ (Cp = η^5^-C_5_H_5_; X = Cl, O^i^Pr), Mg, or Zn in the presence of ClSiMe_3_ as a beneficial additive.^13^ Avoiding the use of metallic reductants, Tonks^14^ has described a series of titanium imide compounds that catalyze these cycloadditions. It is presumed that the Ti(II) species is generated by the reductive elimination of a six-membered metallacycle composed of two alkynes and one imido fragment giving rise to a pyrrole. The same group advanced this field toward the formation of naphthalene derivatives assembling alkynes and benzyne, using [Cp*_2_ZrPh_2_] and the catalytic system [TiI_4_(thf)_2_]/Zn.^15^ While all these systems exhibit high activity, they are not selective when asymmetric alkynes are employed. Notable exceptions are based on the low-valent titanium alkoxides reported by Six^16^ that display high levels of control similar to Ladipo’s results.^8,17^ In contrast, titanium-based catalysts favoring the regioselective formation of the 1,3,5-isomer are uncommon and typically are substrate dependent. For example, the group of Rothwell^18^ reported a titanacyclopentadiene complex that facilitates the 1,3,5-cyclotrimerized product in high selectivity only for sterically hindered terminal alkynes. In contrast, using smaller alkynes leads to a mixture of both isomers, with 1,2,4-isomer being the predominant. Similarly, Ohta^19^ described a [bis(indolyl)TiCl_2_] (indolyl = 2,2′-bis(indolyl)-methanes) compound which in combination with magnesium metal favors the formation of the symmetric 1,3,5-arene product only for the trimethylsilyl-substituted terminal alkyne.

High selectivity for the 1,3,5-isomer is consistently achieved by the catalytic system utilizing mono- or dinuclear titanium *p*-*tert*-butylthiacalix[4]arene and sodium metal described by Morohashi and Hattori.^20^ However, the substrate scope is limited, and no information on the truly active species is provided.

Despite these notable advances, the development of titanium-based, functional-group-tolerant methods for selectively synthesizing 1,3,5-substituted arenes remains underdeveloped. This area of study is particularly compelling, given the widespread occurrence of these aromatic compounds in complex structural motifs with applications as precursors for drug development,^21^ materials for solar energy conversion,^22^ ionic liquids,^23^ markers for RNA delivery,^24^ and supramolecular receptors.^25^

Drawing inspiration from Ladipo’s methodology, herein, we report the isolation of a low-valent titanium arene catalyst for the regioselective cyclotrimerization of alkynes toward the 1,3,5-regioisomer. Within this study, we synthesized the Ti(IV) compound [TiCl_2_(^Mes^PDA)(thf)_2_] (**2**) [^Mes^PDA = *N,N*′-bis(2,4,6-trimethylphenyl)-*o*-phenylenediamide] to act as a precursor for the formation of our desired low-valent titanium compounds. Circumventing the use of strong reductants (*E*°< −2.5 V), we prepare the inverted sandwich Ti(II)/Ti(III) complex [Li(thf)_4_][(Ti^Mes^PDA)_2_(μ–η^6^: η^6^-C_6_H_6_)] (**4**). This was accomplished through an initial alkylation of the Ti(IV) parent complex, followed by a subsequent series of β-abstraction, β-elimination reactions, hydrogen evolution, and chemical reduction, as evidenced by ^1^H NMR, reaction pressure monitoring, and DFT calculations. X-ray analysis of compound **4** reveals that the bridging benzene is assigned as a dianion fragment and hence the valence of the titanium atoms as a mixture of Ti(II) and Ti(III). Consequently, EPR spectroscopy (Figure S10) confirms the presence of an unpaired electron localized on the titanium atoms. Species **4** proves to be a highly active and selective catalyst for the cyclotrimerization of a broad variety of aliphatic and aromatic alkynes, toward the formation of the 1,3,5-trisubstituted benzene. Kinetic analysis reveals that complex **4** is the resting state of the cyclotrimerization reactions following a [2 + 2 + 2] path, in which the oxidative coupling of two alkynes is the rate-determining step. The key to the observed high selectivity is the thermodynamic preference of the bimetallic arrangement for the stabilization of the 1,3,5-regioisomer, as disclosed by DFT calculations.

## Results and Discussion

### Synthesis of Ti Compounds

*Ortho*-phenylenediamido ligands have proved to be good stabilizing ligands for strong reducing reagents. For example, stable PDA-Mg (I) and PDA-Zn (I) compounds have been reported (PDA = *ortho*-phenylenediamide).^26^ In contrast, for group 4 metals, to our knowledge, only our group has reported the preparation of a PDA-Ti(III) compound^27^ and there are no reports on their use in stabilizing lower oxidation states.

**Scheme 1:**
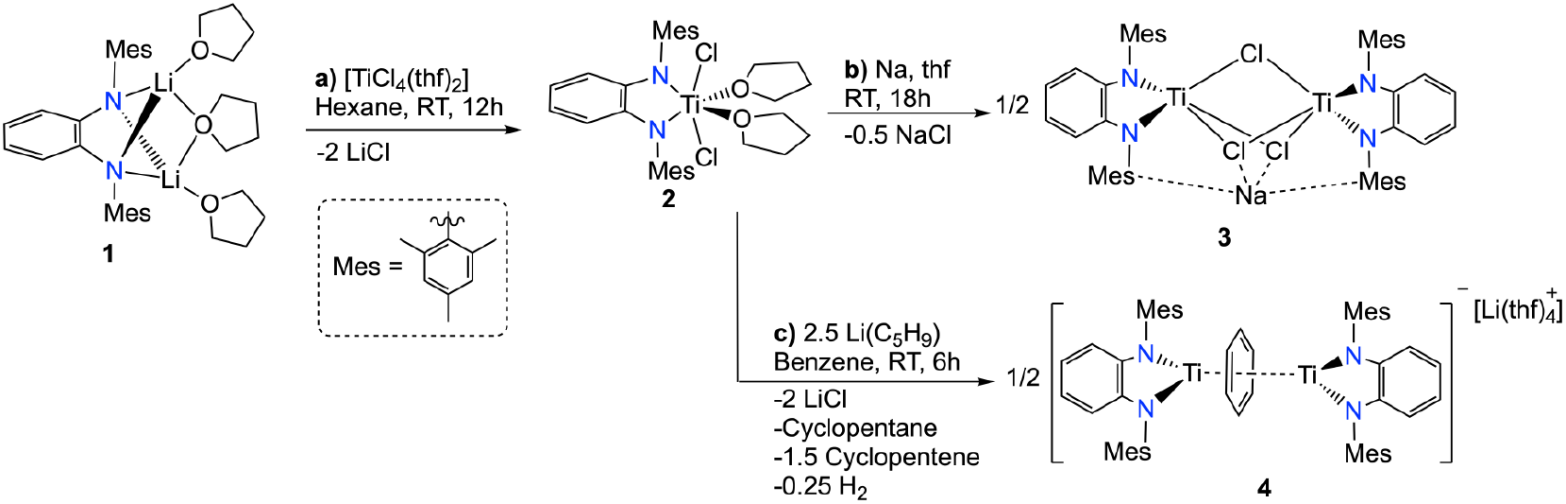
Synthesis of Compounds. a) **2**, b) **3**, and c) **4**

We began studying the incorporation of the ^Mes^PDA ligand into a titanium(IV) precursor. The reaction of the corresponding lithium ^Mes^PDA species **1** with one equivalent of [TiCl_4_(thf)_2_] leads to species **2** in 87% yield, along with LiCl elimination (Scheme 1, reaction a).

**Figure 2:**
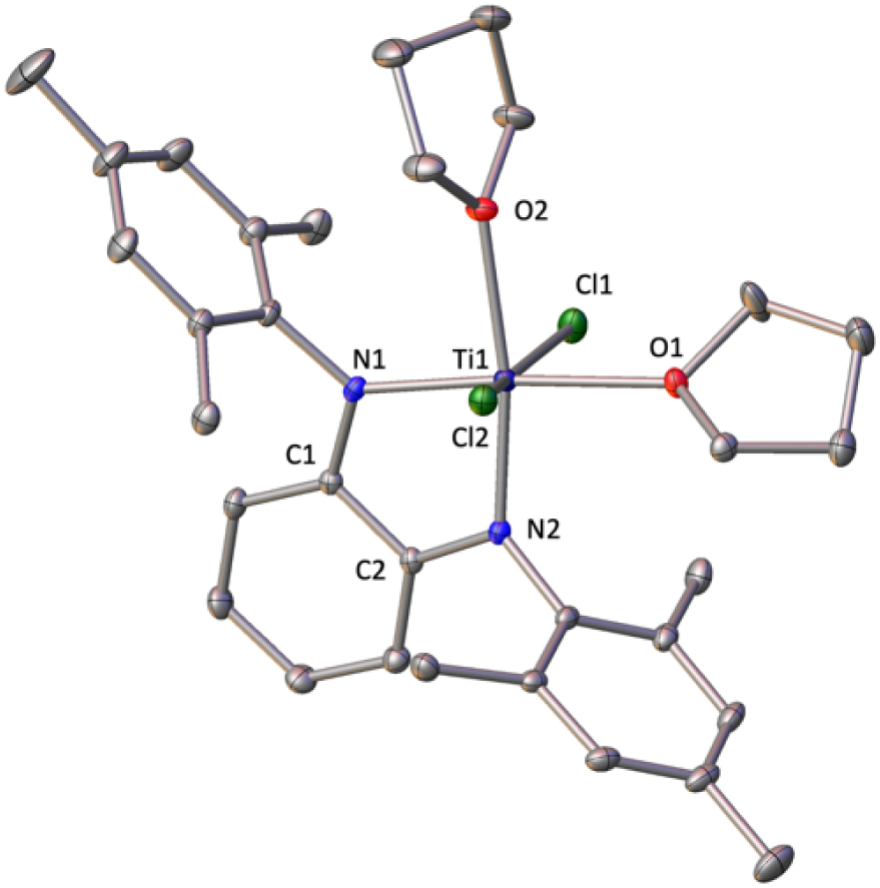
Solid-state structure of compound **2** with ellipsoids at 30% of probability. Hydrogen atoms are omitted for clarity. The dihedral angle between planes formed by N1–Ti1–N2 and N1–C1–C2-N2 is 14.1°. Selected average bond distances (Å) and angles (°): Ti–N 1.970(5), Ti–O 2.208(1), Ti–Cl 2.364(9), N–Ti–N 80.48(9), O–Ti–O 95.1(1), Cl–Ti–Cl 162.08(3).

X-ray analysis of single crystals for **2** reveals a monomeric structure (Figure 2) in which the dianionic ^Mes^PDA^2–^ ligand coordinates to titanium through the two nitrogen atoms. The observed Ti–N bonds (average = 1.970(5) Å) lie in the highest limit of the range 1.864 (4)–1.970 (7) Å reported for the related chelate diamido titanium dichloride species [*cis*-9,10-PhenH_2_(NR)_2_TiCl_2_]^28^ (PhenH_2_ = 9,10-dihydrophenanthrene; *R* = 2,6-^i^Pr_2_C_6_H_3_, 2,6-Me_2_C_6_H_3_, *^t^*Bu) and [(pada)TiCl_2_(py)_2_]^29^ (pada = *N, N*-bis(3,5-dimethylphenyl)-phenanthrene-9,10-diamide).

The metallic center adopts a pseudooctahedral geometry by additional binding of two thf molecules within the plane of the PDA ligand (∑α *=* 360°), while the two axial positions are occupied by two chlorine atoms. Albeit PDA ligands can also act as a donor through the π electron density at the C=C bond,^30^ the nearly planar titanium diazametallacycle (dihedral angle 14.1(1)°) and the long Ti⋯C_α_ distances (average = 2.839(3) Å) rules out the π component, which is in agreement with the presence of the two σ donor thf molecules. A similar bonding situation was reported by Scholz^31^ for the thf-solvated titanium compound [TiCl_2_(^Cy^DAD)(thf)_2_] (^Cy^DAD = *N,N*′-bis(cyclohexyl)-1,4-diaza-1,3-butadiene), where the diamido ligand engages only in σ^2^ coordination with the titanium atom (Ti⋯C_α_ = C_α’_ = 2.892(2) Å, dihedral angle for diazametallacycle 0°). In contrast, Tilley^32^ described the unsolvated diamido titanium compound [TiCl_2_(^SiIpr^PDA)] (^SiIpr^PDA = *N,N*′-bis(triisopropylsilyl)-*o*-phenylenediamide), in which the PDA acts as both σ^2^ and π donor ligand, evidenced by shorter Ti⋯C_α_ distances (2.351(2) Å) and a significant puckering of the diazametallacycle (dihedral angle 56.9°).

In agreement with the X-ray analysis, the ^1^H and ^13^C NMR spectra (Figures S1 and S2) of **2** in C_6_D_6_ show one set of signals for the ^Mes^PDA ligand and the resonances corresponding to the coordinated thf molecules.

Moving into the chemical reduction of compound **2**, we used sodium metal as a chemical probe to gain some insights into the redox potential necessary for accessing the low oxidation states III or II. Treatment of compound **2** with an excess of sodium metal (2 equiv) in thf as solvent leads to the formation of the ^1^H NMR silent (range −50 ppm to 50 ppm, Figure S4) and heterobimetallic contacted ion-paired Na/Ti(III) complex **3** (Scheme 1, reaction b).

**Figure 3:**
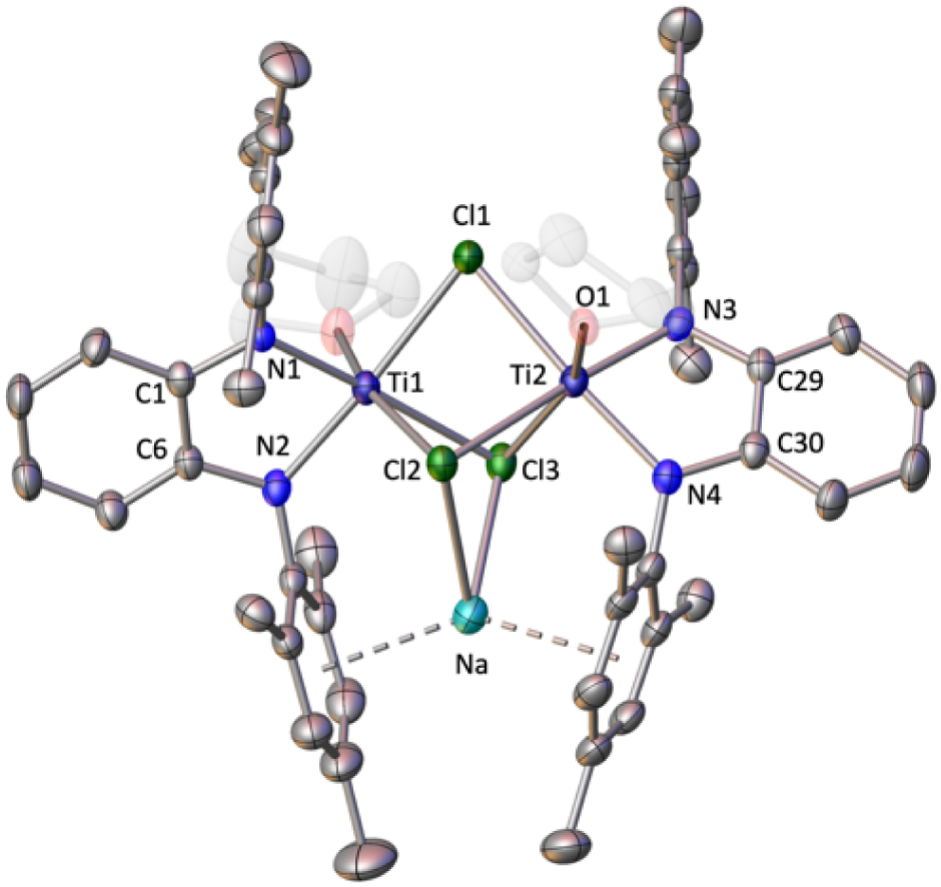
Solid-state structure of compound **3** with ellipsoids at 30% of probability. Hydrogen atoms are omitted for clarity. Selected average bond distances (Å) and angles (°): Ti–N 2.01(1), Ti–O 2.139(8), Ti–Cl 2.53(5), Na–Cl 2.78(1), Na-centroid 2.589(3) and 2.644(3), O2–Ti1–Cl2 163.07(7), N1–Ti1–N2 80.4(1), Cl1–Ti1–Cl3 84.81(3), Cl1–Ti2–N4 175.41(8), O1–Ti2–N3 102.0(2), Cl2–Ti2–Cl3 77.63(3).

The molecular structure shows a trimetallic species formed by two units of [Ti(^Mes^PDA)(thf)] and one sodium atom bridged by three chlorine atoms (Figure 3). Both titanium atoms display a coordination number of six comprised of two nitrogen atoms of the chelate diamido ligand, three bridging chlorines, and a molecule of thf. The less acidic Ti(III) metal center results in elongated Ti–N bonds [average = 2.01(1) Å] than those registered for **2** (1.970(5) Å). Compared to structurally characterized aryl-amide Ti(III) species,^33^ these bond lengths are also lengthened by ca. 0.1 Å; however, they are similar to the Ti(III) amide compounds reported by our group [Li(thf)_4_][Ti(^Ar^PDA)_2_]^27^ (Ar = 2,4,6-trimethylphenyl and 2,6-diisopropylphenyl) and for the nitrile- and isocyanide-solvated titanium amido compounds [Ti(N(SiMe_3_)_2_)_3_L_2_].^34^ Of note, the Ti–Cl bond distances, exhibiting an average value of 2.53(5) Å, are within the range found for other dimeric chlorine-bridged titanium(III) compounds (average Ti–Cl 2.428(2)–2.550(2) Å).^35^ Furthermore, the sodium cation is held within the dimeric fragment by interaction with two lateral mesityl rings (Na-centroid 2.589(3) and 2.644(3) Å) and with two chlorine atoms (Na–Cl 2.78(1) Å), similarly to previously reported compounds with individual Na^+^ cations or NaCl units sitting between two aryl rings.^36^

An X-band EPR study of compound **3** in thf solution at 160 K revealed a rhombohedral spectrum with *g* values of *g*_1_ = 1.9824, *g*_2_ = 1.9605, and *g*_3_ = 1.8900 (see Figure S10), similar to those observed for monomeric Ti(III) compounds where the unpaired electron is centered on the metal.^37^ Additionally, the effective magnetic moment (μeff), determined by the Evans method in [D_8_]-thf, was found to be 1.88 μ_B_. These observations suggest that in thf solution, compound **3** transitions from its original solid-state dimeric arrangement into a thf-solvated monomeric form, with the concurrent release of NaCl.

The one electron reduction of compound **2** into species **3**, even in the presence of an excess of sodium metal, suggests that achieving a low-valent Ti(II) species requires the use of stronger reductants. However, to access “[L_n_Ti(II)]” fragments, we explored an alternative approach. Formation of dialkyl titanium compounds as a precursor of masked Ti(II) alkene compounds via β-hydrogen abstraction is an extended practice in synthesis.^1^ For instance, in the Kulinkovich reaction, cyclopentylmagnesium chloride is combined with Ti(O^i^Pr)_4_ to prepare a titanium cyclopentene precatalyst.^1^ In a similar vein, reacting compound **2** with a slight excess (2.5 equiv) of cyclopentyl lithium (Scheme 1, reaction c) leads to the generation of the ^1^H NMR silent (range from −50 to 50 ppm, Figure S6) and paramagnetic species **4**.

**Figure 4:**
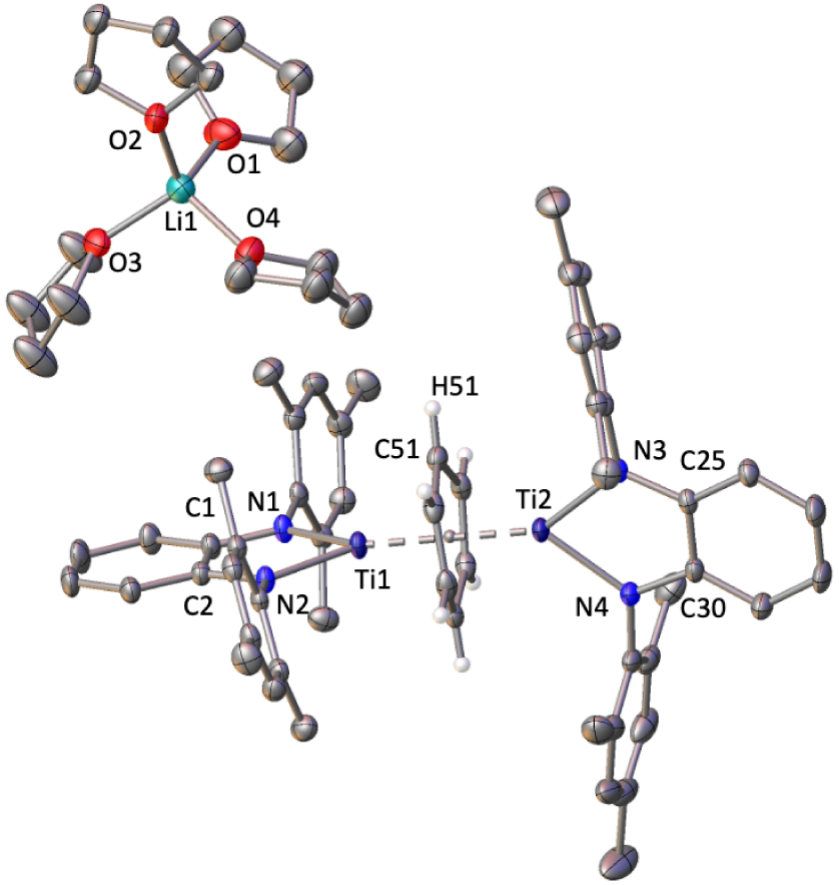
Solid-state structure of compound **4** with ellipsoids at 30% of probability. Hydrogen atoms, except those of the benzene ring, are omitted for clarity. Selected average bond distances (Å) and angles (°): Ti–N 2.014(4), Ti–C 2.23(5), C–C 1.451(4), Ti-centroid 1.691(3), N–Ti–N 78.63(6).

Analysis of single crystals suitable for X-ray diffraction discloses **4** as a solvent-separated ionic compound containing fragments [(Ti^Mes^PDA)_2_(μ–η^6^: η^6^-C_6_H_6_)]^−^ and [Li(thf)_4_]^+^ (Figure 4).

The anionic fragment displays an inverted sandwich structure in which two titanium atoms are η^6^ bonded to opposite sides of a benzene ring. There are three possible electronic interpretations for this anion: (i) Ti(II)/Ti(III), bridged by a benzene dianion; (ii) Ti(I)/Ti(II), bridged by a neutral benzene; and (iii) Ti(III)/Ti(IV), bridged by a benzene tetraanion. The structural analysis of compound **4** suggests a Ti(II)/Ti(III) system as the best electronic description. This interpretation is made evident by the loss of aromaticity of the bridging arene fragment. Thus, this fragment deviates from planarity with a dihedral angle of 10.6(2)° and displays a lengthening of the C–C bond distances (average of 1.451(4) Å). A tetraanionic benzene interpretation is unlikely since the experimental C–C bond distances in complex **4** diverge from the calculated 1.507 Å of such moiety.^38^ Additionally, our experimental data align with the previous findings on the arene dianion bonded to two titanium atoms in compound **V** [{(N(SiMe_3_)_2_)_2_Ti}(μ,η^6^-C_7_H_8_){Ti(N(SiMe_3_)_2_)(μ-N(SiMe_3_)_2_)(KC_7_H_8_)}] (Figure 1) where the torsion angle is 20.1(2)°, and the average C–C bond is 1.442(5) Å.^11^

**Figure 5:**
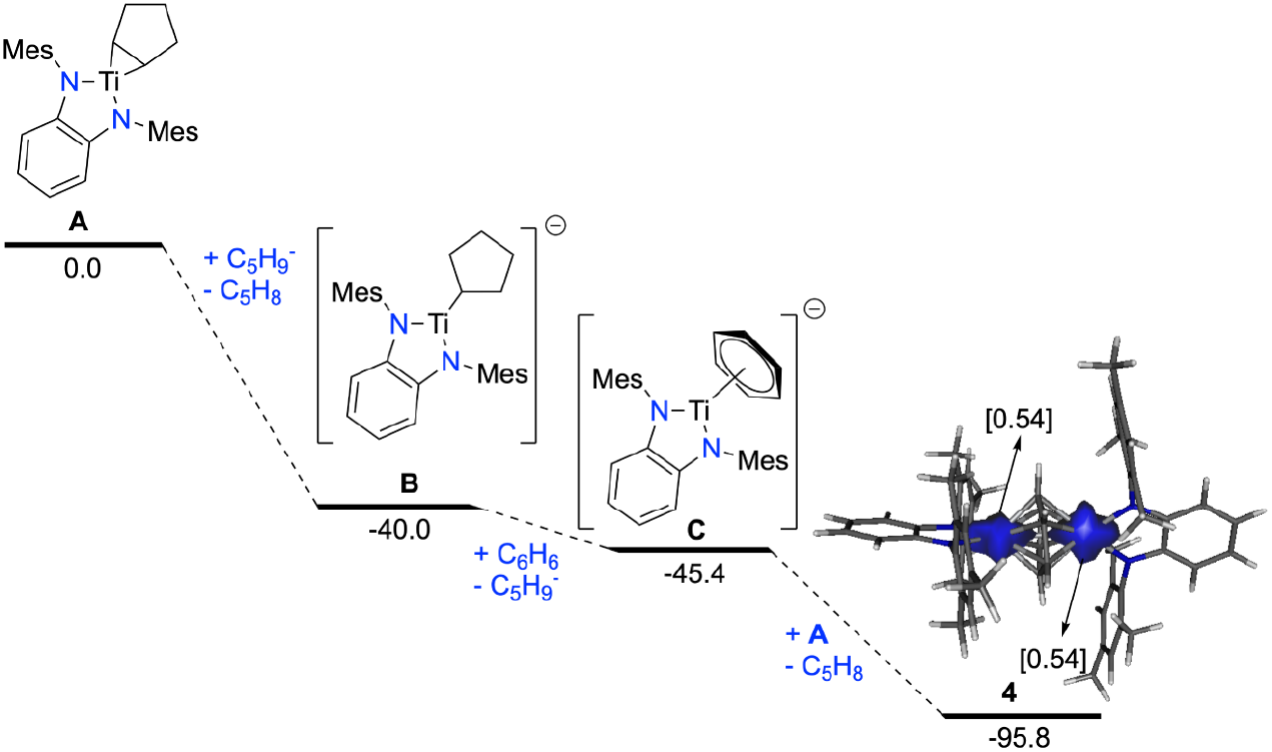
Computed reaction profile for the formation of **4** from **A**. Relative free energies (at 298 K) are given in kcal/mol. The computed spin density in **4** is also depicted and shows that the unpaired electron is equally distributed on both titanium atoms (0.54 e). All data were computed at the M06L/def2-SVP level.

Despite the formal assignment of oxidation states II and III to compound **4**, the computed spin densities for the DFT-optimized structure of species **4** reveal no differences between both titanium atoms, indicating an equal distribution of electron density across them (see Figure 5). Consequently, complex **4** displays four similar Ti–N bond lengths that can be averaged in 2.014(4) Å. This contrasts with the Ti–N bond distances reported for the Ti(II)/Ti(III) compound **V**(11) as shown in Figure 1, where the titanium with longer Ti–N distances (d = 2.129(2) and 2.146(2) Å) is assigned an oxidation state of II. However, it is worth considering that their observed elongation of the Ti–N bonds might also result from the asymmetric interaction with the alkali metal, affecting only one side of the dinuclear fragment. Specifically, the nitrogen atoms that simultaneously bind to titanium and potassium exhibit longer Ti–N distances.

Characterization of complex **4** in benzene solution was initially performed using the Evans method, which revealed the presence of one unpaired electron, as indicated by an effective magnetic moment (μ_eff_) of 1.78 μ_B_. Further insights were obtained by comparing EPR spectra in both the solid state and benzene solution at 160 K. The spectra in these states display a single broad signal at *g* = 1.977 for the benzene solution and 1.978 for the solid state, corroborating the retention of the solid-state structure in solution and suggesting that the unpaired electron is localized on the titanium atom (see Figure S10). Moreover, this observation aligns with the DFT-optimized structure of compound **4**, where the electron density is equally distributed across both titanium atoms (see Figure 5).

Isolation of compound **4** confirms the ability of the PDA ligands to stabilize titanium in oxidation states below III. This is particularly noteworthy when compared with low-valent early transition metals (e.g., Ti, V, Nb) chelated by the structurally similar bidentate β-diketiminate (NacNac) ligands, which results in reductive cleavage of the ligand.^39^

**Scheme 2:**
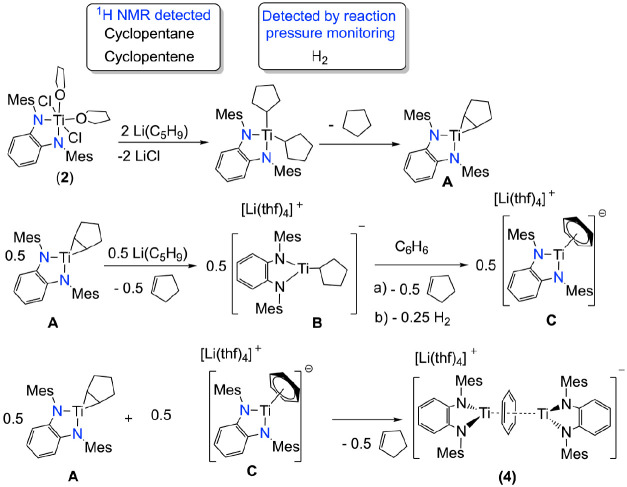
Proposed Mechanism for the Formation of Compound **4**

Intrigued by the formation of complex **4**, we monitored the reaction of synthesis by ^1^H NMR spectroscopy, revealing the formation of cyclopentane and cyclopentene (see Figure S15). Additionally, we registered the reaction pressure over time in a closed reaction vessel using the Man on the Moon X102 device,^40^ revealing a pressure increase proportional to the generation of 0.25 equiv of H_2_. These observations support a mechanism in which an initial bis(cyclopentyl)titanium compound is formed (Scheme 2). Then, it undergoes β-hydride abstraction to give rise to cyclopentane and titanacyclopropane intermediate **A**. According to the use of a 0.5 excess of the lithium reagent, half of the intermediate **A** undergoes alkene substitution by a cyclopentyl anion, producing ionic species **B**. Subsequently, **B** proceeds through β-hydride elimination toward the formation of a Ti–H fragment. These fragments are known to release H_2_ and undergo chemical reduction,^41^ ultimately forming, in the presence of benzene, the ionic species “[Li(thf)_4_][Ti(^Mes^PDA)(η^6^-C_6_H_6_)]” (**C**). Finally, the combination of **C** with the remaining intermediate **A** leads to species **4** and liberation of cyclopentene to the media.

To further support our mechanistic proposal, we carried out DFT calculations at the dispersion-corrected M06L/def2-SVP level (see computational details in the Supporting Information) to assess the thermodynamic feasibility of the proposed mechanism. These calculations revealed a thermodynamically favored pathway, where the formation of each intermediate becomes exergonic. Notably, the formation of the dinuclear species **4** from the monomeric intermediate **C** is significantly favored (Δ*G*_R_ = −50.4 kcal/mol), which is consistent with previous reports by Gambarotta and Budzelaar.^10^

### Catalysis

Encouraged by the potential of compound **4** as a low-valent titanium arene species to act as an intermediate in the synthesis of aromatic compounds through cyclotrimerization of terminal alkynes, we tested its catalytic activity in the trimerization of phenylacetylene, using a 10 mol % catalyst loading in benzene at 25 °C. To our delight, within 6 h, species **4** provides the 1,3,5-aromatic product in high yields and high selectivity (Table 1, entry 1). Next, we investigated the solvent effect on the reaction outcome. Under similar reaction conditions, using a coordinating solvent such as pyridine or acetonitrile resulted in total inhibition of the reaction progress (Table 1, entries 2 and 3). In a second step, the influence of catalyst loading was evaluated, revealing that the concentration of catalyst can be reduced up to values of 2.5 mol % with no variation on yield and selectivity parameters (Table 1, entries 4–6). Finally, we explored the reaction time effect, being able to shorten it to just 3 h (Table 1, entry 7).

**Table 1:**
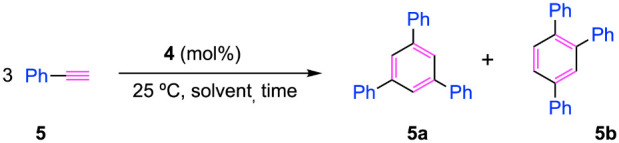
Optimization for Catalytic Trimerization of Phenylacetylene.^a^

entry	catalyst (mol %)	solvent	time (h)	yield (%)^b^	regioselectivity (5a:5b)
1	**4** (10)	C_6_D_6_	6	>99	85:15
2	**4** (10)	[D_5_]-pyridine	6	-	-
3	**4** (10)	[D_3_]- acetonitrile	6	-	-
4	**4** (7)	C_6_D_6_	6	>99	85:15
5	**4** (5)	C_6_D_6_	6	>99	85:15
6	**4** (2.5)	C_6_D_6_	6	>99	85:15
7	**4** (2.5)	C_6_D_6_	3	>99	85:15
8	**4** (2.5)	C_6_D_6_	3	90^c^	93:7^c^
9	**2** (2.5)	C_6_D_6_	3	-	-
10	**3** (2.5)	C_6_D_6_	3	7	ND^d^

a Reaction conditions: phenylacetylene (0.3 mmol), **4** (10–2.5 mol %), 0.5 mL of benzene, room temperature, and 6–3 h.

b Yields were determined by ^1^H NMR spectroscopy using a 10 mol % of ferrocene as internal standard.

c Isolated yields: The isomer ratio of the isolated product was determined by ^1^H NMR spectroscopy.

d The ratio could not be determined due to the low yield.

Using the optimized conditions (2.5 mol %, C_6_H_6_, 3h), compound **5a** was isolated in high yield (90%) and high selectivity (Table 1, entry 8).

In evaluating catalytic performance, our titanium catalytic system demonstrates enhanced activity, evidenced by turnover number (TON = 40) and turnover frequency (TOF = 13 h^–1^) values, when compared to those reported for the in situ reduced [CpTiCl_3_] (TON = 15; TOF = 1 h^–1^)^13^ and [bis(indolyl)TiCl_2_] (TON = 6; TOF = 1 h^–1^).^19^ However, our results do not reach the higher performance metrics of titanium alkoxide catalysts described by Ladipo (TON = 99; TOF = 396 h^–1^),^8^ Rothwell (TON = 235),^18^ and Morohashi and Hattori (TON = 380).^20^ The relatively lower TON value of our system can be attributed to its expected lesser stability toward hydrolysis compared to titanium alkoxide compounds, which allows for the use of lower catalyst loadings. Despite these inferior activity levels, our system demonstrates unprecedented selectivity for the 1,3,5-isomer, a feature not observed in any of the previous catalytic systems where the 1,2,4-isomer is typically favored.

To prove the critical role of the Ti(II) center in compound **4** for the cycloaddition of phenylacetylene, we conducted the reaction under optimized conditions using Ti(IV) compound **2** and Ti(III) complex **3** as catalysts. These experiments revealed no conversion with compound **2** (Table 1, entry 9) and only a poor 7% yield with species **3** (Table 1, entry 10).

Motivated by the high levels of regioselectivity toward the unusual 1,3,5-isomer, we investigated the functional group tolerance of our catalytic system. Increasing the steric bulk of the terminal aromatic ring by including a methyl substituent in *ortho* position (Table 2, Entry 1) did not significantly affect the isolated yield (82%) and maintained the selectivity levels (88:12). Contrasting with the lack of reactivity in the presence of coordinating solvents, substrates with a methoxide and ethoxide in the *para* positions of the phenyl group attached to the reacting alkyne underwent quantitative and selective cyclotrimerization to give the 1,3,5-isomer (Table 2, entries 2 and 3). Furthermore, the 1,3,5-selectivity for the formation of arenes was confirmed by X-ray diffraction studies on **8a** (see Figure S19). Remarkably, this high catalytic activity persists in the presence of strongly coordinating functional groups such as the dimethylamino fragment (Table 2, entry 4). However, the incorporation of pyridine and nitrile moieties into the alkyne resulted in no reaction, recovering the starting material (Table 2, entries 5 and 6). Moreover, catalyst **4** is also compatible with substrate **12** with a trifluoromethyl substituent, delivering 1,3,5-arene in good yield (Table 2, entry 7). Furthermore, introducing a second aromatic ring, as in the naphthyl-substituted derivative **13**, did not compromise the high levels of yield and selectivity (Table 2, entry 8). Nonaromatic substituents on the alkyne like the sterically hindered trimethylsilyl group in reagent **14** also led to the 1,3,5-arene compound selectively in a 98% yield (Table 2, entry 9). Conversely, the dimethylaminomethyl-substituted compound **15** resulted in a reduced yield of 83% (Table 2, entry 10) but retained the high selectivity (>99:1).

**Table 2:**
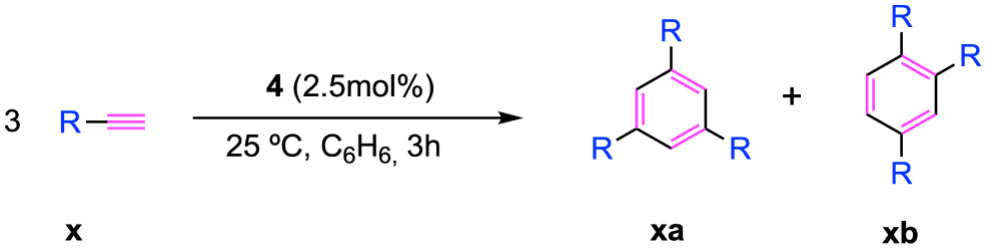

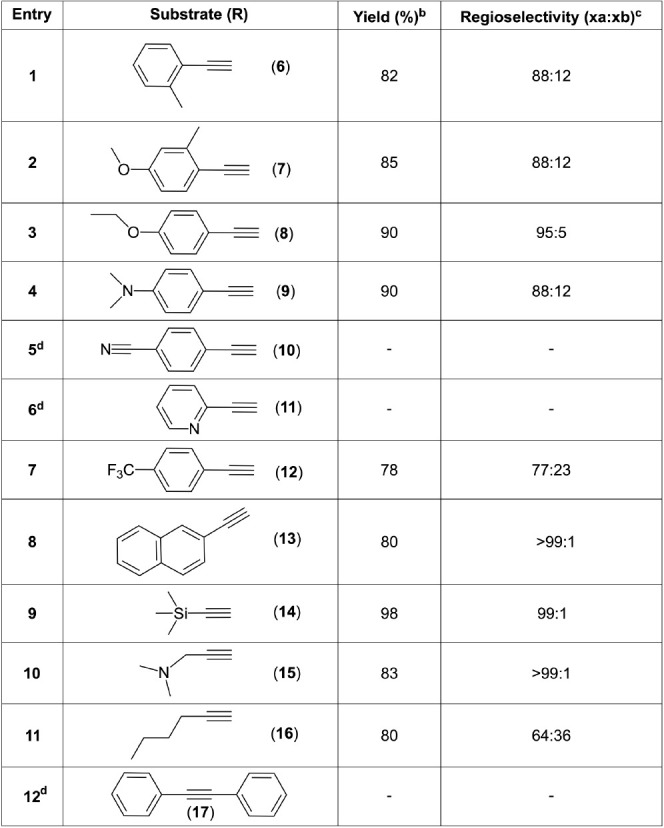
Cyclotrimerization of Alkynes Using Catalyst **4**

a Conditions: alkyne (3.0 mmol), **4** (2.5 mol %, 0.075 mmol), 5 mL of benzene, room temperature, and 3 h.

b Isolated yield of a and b after column chromatography.

c Determined by ^1^H NMR spectroscopy.

d No reaction was observed, recovering the alkynes employed.

Surprisingly, 1-hexyne **16** produced a drop in selectivity to a value of 64:36 (Table 2, entry 11). Finally, with increasing steric hindrance on both sides of the alkyne, as in diphenylacetylene **17**, did not afford the cyclotrimerized product under the optimized conditions (Table 2, entry 12).

Compared to previous reports on cyclotrimerization reactions mediated by titanium compounds,^13,19^ our catalytic system eliminates the need for reductants such as magnesium or zinc metal. As a result, a broader functional group spectrum is tolerated, being compatible with trifluoromethyl groups (Table 2, entry 7) which as far as we are aware, the desired cyclotrimerization product has not been isolated using a titanium-based catalytic system.^42^ More notably, compound **4** proves to be compatible with certain coordinating groups present in either aromatic or aliphatic alkynes. Such behavior has only been observed for titanium calix[4]arene compound reported by Ladipo,^8^ which is compatible with aliphatic alkynes including ether, sulfide, and amine groups. However, a key distinction is that while the latter system leads to the 1,2,4-isomer as the major product, complex **4** provides a complementary methodology producing the unusual 1,3,5-regioisomer with high selectivity. Drawing a comparison with the titanium catalytic systems reported by Rothwell^18^ (titanacyclopentadiene species), Ohta^19^ (bis(indolyl) titanium complex) and Morohashi and Hattori^20^ (*p*-*tert*-butylthiacalix[4]arene titanium compound), capable of mediating the formation of the 1,3,5-isomer, reveal that our system exhibits enhanced functional group tolerance and increased regioselectivity. The latter is especially noticeable when contrasted with the titanacyclopentadiene^18^ and the bis(indolyl) titanium catalysts,^19^ in which the regioselectivity is substrate dependent, providing only the 1,3,5-isomer in the case of alkynes with sterically hindered substituents. However, compared to the titanium *p*-*tert*-butylthiacalix[4]arene catalysts,^20^ it should be noted that our system demands larger catalyst loading.

### Mechanistic Insights

Determined to gather mechanistic information, we subjected our system to a variety of mechanism analyses (see Section 6 in the Supporting Information for further details). In specific, we employed the initial rates and integration methodologies to determine the orders of the catalyst and the alkyne, respectively. Our investigation began by examining the dependency on the catalyst concentration. We measured the initial rates for the catalytic cyclotrimerization of trimethylsilylacetylene at different catalyst loadings, monitoring the alkyne consumption until achieving a maximum of 7.5% conversion (see Figure S16). The resulting plot of the initial rates against the concentration of catalyst **4** displays a linear trend (see Figure S17) consistent with a pseudo-first-order dependence on the catalyst. Albeit a mononuclear pathway cannot be fully ruled out, the pseudo-first-order dependence combined with the EPR characterization in solution of compound **4** (see EPR spectroscopy section in the Supporting Information for further details) and the greater stability of the dinuclear species versus the mononuclear counterpart deduced by DFT calculations (Figure 5) indicate that compound **4** does not dissociate during the catalytic process, hence suggesting the cooperation of the two metals in the cycloaddition reactions. For the alkyne order, the data acquired upon the monitoring of the trimethylsilylacetylene concentration over time for the cycloaddition of the alkyne using a 5 mol % of **4** until 85% yield align with a second-order integrated eq (Figure S18). Therefore, the rate-determining step involves the oxidative coupling of two alkynes mediated by the two titanium centers in compound **4**. Similar kinetic results were reported by Ladipo,^8^ describing the trimethylsilyl-substituted arene–titanium compound as the resting state of the process. In contrast, the author observed a first-order dependence on the alkyne. Notably, while there are no reports on bimetallic titanium compounds cooperating for the formation of arenes from alkynes, Meijer^43^ described the concerted oxidative coupling of two alkynes by two cyclooctatetraene titanium fragments. Moreover, a dinuclear reaction path has been described for early transition metals of group 5.^2,5^ Specifically, Mashima^44^ has isolated a variety of dinuclear tantalum metallacyclopentadiene compounds for the cyclotrimerization of alkynes.

An alternative methodology for investigating the resting state involves analysis of the reaction products generated by quenching the cycloaddition reaction through hydrolysis before it reaches completion. Thus, the detection of the corresponding alkene or 1,3-diene would suggest a titanacyclopropane or titanacyclopentadiene intermediate as the resting state, respectively. Ohta^19^ reported a similar approach to identify the titanacyclopropane species as the resting state. In our case, we quenched the cycloaddition of trimethylsilylacetylene catalyzed by **4** (10 mol %) by exposing the reaction mixture to air before completion. ^1^H NMR of the crude mixture showed only the presence of unreacted trimethylsilylacetylene, ^Mes^PDAH_2_ ligand, and the final aromatic product, with no signs of vinyltrimethylsilane or bis(trimethylsilyl)buta-1,3-diene intermediates. This outcome further supports the hypothesis that a dinuclear compound, similar to compound **4**, acts as the resting state in our system.

**Scheme 3:**
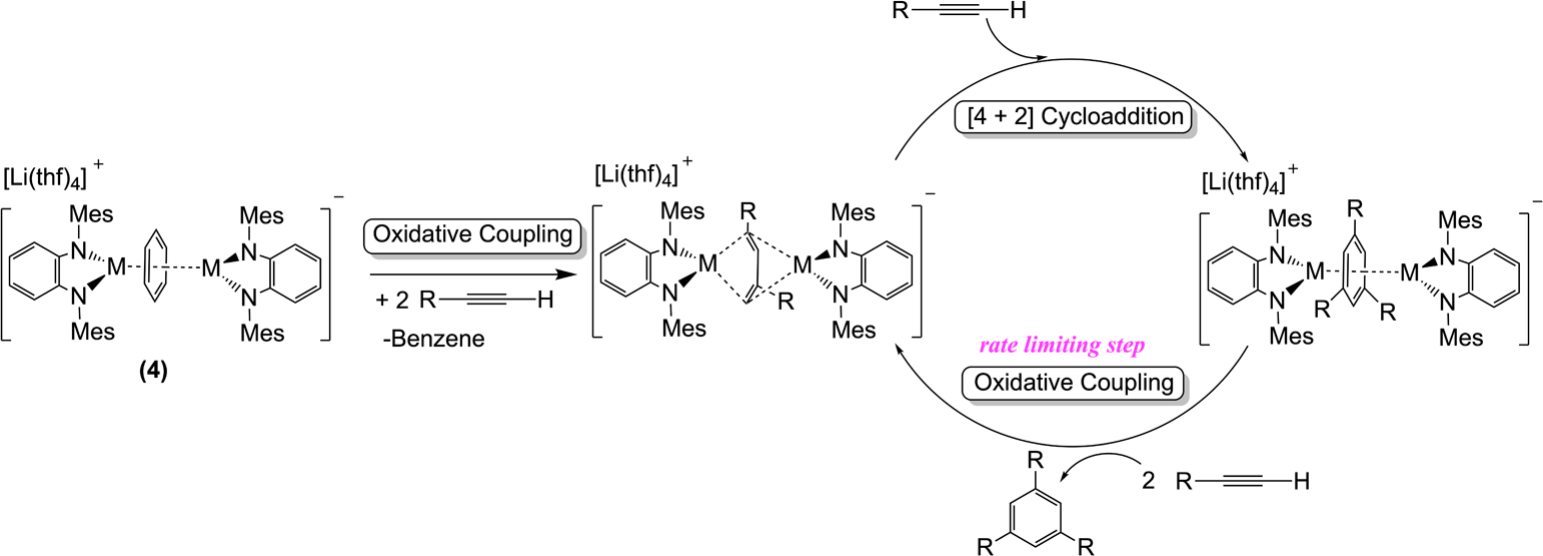
Mechanism for the Cyclotrimerization of Alkynes Catalyzed by Compound **4**

Overall, these data support the mechanism described in Scheme 3. In the first step, bimetallic compound **4** produces the coupling of two alkynes while releasing benzene. Then, [4 + 2] cycloaddition takes place by regenerating the titanium arene active species to reinitiate a new cycle.

**Figure 6:**
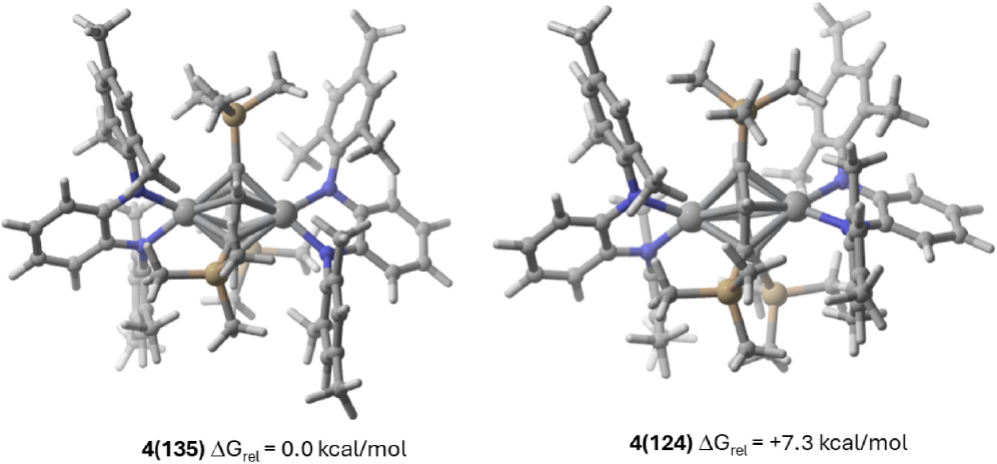
DFT-modeled compounds similar to 4 with tris(trimethylsilyl)benzene as a bridging fragment. All data have been computed at the M06L/def2-SVP level.

Building on this proposed mechanism, we carried out preliminary DFT calculations to investigate the reasons behind the preference for the 1,3,5-isomer over its 1,2,4-counterpart during the catalysis. Due to the high selectivity achieved with trimethylsilylacetylene, we used it as a model substrate for the computational study. Comparison of the computed two possible isomers of the tris(trimethylsilyl)benzene product within the framework formed by the two [Ti^Mes^PDA] units revealed that the 1,3,5-form is thermodynamically more stable by 7.5 kcal/mol, as shown in Figure 6, which is mainly due to unfavorable steric interactions in the 1,2,4-isomer. This energy difference underpins the preferential formation of the 1,3,5-isomer in the catalyzed reactions.

## Conclusions

In conclusion a PDA-supported mixed-valence Ti(II)/Ti(III) inverted sandwich compound was synthesized, characterized, and used as an efficient and selective catalyst for the cyclotrimerization of alkynes. These studies demonstrate that PDA ligands are effective for the stabilization of low-valent titanium compounds, evidenced by the successful stabilization of both the Ti(III) [[{Ti(^Mes^PDA)(thf)}_2_(μ-Cl)_3_{Na}] (**3**) and the inverted sandwich Ti(II)/Ti(III) species [Li(thf)_4_][(Ti^Mes^PDA)_2_(μ–η^6^: η^6^-C_6_H_6_)] (**4**). Notably, we have synthesized the Ti(II)/Ti(III) compound **4** without relying on traditionally strong reductants (*E*°< −2.5 V), using an alternative approach that involves β-hydride abstraction/elimination, hydrogen evolution, and chemical reduction, evidenced by spectroscopical data and quantum-chemical calculations. EPR spectroscopy and DFT analysis confirm the presence of a single unpaired electron located on both titanium atoms. Compound **4** provides one of the uncommon titanium systems with high activity, broad functional group tolerance, and high selectivity toward the cycloaddition of alkynes to form the 1,3,5-trisubstituted arene compound. Through kinetic studies, the catalytic role of inverted sandwich compound **4** is established, being the resting state during the rate-limiting oxidative coupling of two alkynes. The catalytic cycle is closed by a final [4 + 2] cycloaddition, furnishing the final aromatic product. Key for the observed regioselectivity is the thermodynamic preference of the two [Ti^Mes^PDA] fragments for the stabilization of the 1,3,5-isomer with a computed 7.5 kcal/mol energy difference.

## Experimental Section

### General Considerations

All reactions were performed under a protective atmosphere using either standard Schlenk techniques (argon) or in an MBraun drybox (argon). [D_1_]-Chloroform and methanol were purchased from Sigma-Aldrich Chemicals and used as received. [D_6_]-Benzene and [D_8_]-tetrahydrofuran were purchased from Eurisotop, and ethyl acetate, toluene, hexane, and tetrahydrofuran from Scharlab. Solvents were dried by heating to reflux over the appropriate drying agents: [D_6_]-Benzene, benzene, toluene, and hexane (Na/K alloy) and [D_8_]-tetrahydrofuran (Na) and tetrahydrofuran (Na/benzophenone) and distilled prior to use. Phenylacetylene, trimethylsilylacetylene, 1-ethynyl-4-methoxy-2-methylbenzene, 1-hexyne, 4-ethynyl-N,N-dimethylaniline, 3-dimethylamino-1-propyne, and 2-ethynylpyridine were purchased from Sigma-Aldrich Chemicals; 2-ethynyltoluene, 4-ethynyl-α,α,α-trifluorotoluene, and 2-ethynylnaphthalne were purchased from Apollo Scientific; 4-etoxyphenylacetylene was acquired from Fluorochem; 4-ethenylbenzonitrile was purchased from Alfa Aesar. [TiCl_4_(thf)_2_],^45^*N*,*N*′-bis(2,4,6-trimethylphenyl)-*o*-phenylenediamine (^Mes^PDAH_2_),^46^ [Li_2_(^Mes^PDA)(thf)_3_],^47^ and cyclopentyl lithium^48^ were synthesized as described in the literature. NMR spectra were recorded on a Varian Mercury-VX spectrometer operating at 300 MHz for ^1^H, 75 MHz for ^13^C{^1^H}, and 376 MHz for ^19^F-{^13^C}, on a Bruker Neo spectrometer operating at 400 MHz for ^1^H, 100 MHz for ^13^C{^1^H], and on a Bruker 400 Ultrashield system operating at 400 MHz for ^1^H for kinetic studies. ^1^H, ^13^C{^1^H}, and^19^F chemical shifts are expressed in parts per million (δ, ppm) and referenced to residual solvent peaks. All coupling constants (*J*) are expressed in absolute values (Hz), and resonances are described as s (singlet), d (doublet), and m (multiplet). The NMR assignments were performed, in some cases, with the help of ^1^H, ^13^C-HSQC, and ^1^H,^13^C-HMBC experiments. Elemental analyses (C, H, and N) were performed with a LECO CHNS-932 microanalyzer. Samples for IR spectroscopy were prepared as KBr pellets and recorded on the Bruker FT-IR-ALPHA II spectrophotometer (4000–400 cm^–1^). The effective magnetic moments were determined by the Evans NMR method at 293 K (using a 300 MHz instrument with a field strength of 7.05 T).^49^ CW-EPR spectra were performed in a Bruker EMX spectrometer or in a Bruker Magnettech ESR5000 spectrometer. Monitoring of H_2_ release was carried out in a Man on the Moon X102 kit microreactor in the glovebox.^40^ Mass spectrometry (MS) analyses were performed by using an ITQ 900 Thermo Scientific mass spectrometer.

### Crystal Structure Determination of Complexes **3**, **4**, and **8a**

Crystals suitable for X-ray diffraction were obtained by gently heating a benzene suspension of compound **2**, followed by its slow cooling to ambient temperature. Similarly, crystals of compound **8a** were isolated upon slow cooling of a hot hexane solution containing **8a** to room temperature. Dark brown crystals of **3** were obtained through the slow evaporation of a tetrahydrofuran solution, while crystals of compound **4** were prepared by layering a tetrahydrofuran solution with hexane.

The intensity datasets for **3** were collected at 200 K on a Bruker–Nonius Kappa-CCD diffractometer equipped with graphite-monochromated Mo Kα radiation (λ = 0.71073 Å) and an Oxford Cryostream 700 unit, while those for **2**, **4**, and **8a** were collected at 150 K on a Bruker D8 Venture diffractometer equipped with multilayer optics for monochromatization and collimator, Mo Kα radiation (λ = 0.71073 Å), and an Oxford Cryostream 800 unit. Crystallographic data for all complexes are listed in Table S2.

The structures were solved by applying intrinsic phasing (SHELXT)^50^ using the Olex2^51^ package and refined by least-squares against F^2^ (SHELXL).^52^ All nonhydrogen atoms were anisotropically refined, while hydrogen atoms were placed at idealized positions and refined using a riding model.

### Preparation Details of Complexes **2**–**4**

#### Synthesis of [TiCl_2_(^Mes^PDA)(thf)_2_] (**2**)

A 100 mL Schlenk was charged in the glovebox with [Li_2_(^Mes^PDA)(thf)_3_] (0.6 g, 1.0 mmol) and [TiCl_4_(thf)_2_] (0.33 g, 1.0 mmol) in 30 mL of hexane. The resultant solution was allowed to stir overnight. After that time, the solvent was removed under reduced pressure. Then, the reaction mixture was extracted with benzene, filtered through a medium porosity glass frit, and dried under vacuum to yield **2** as a brown solid (0.53 g, 87%). IR (KBr, cm^–1^):  = 3050 (m), 2915 (m), 2857 (m), 1594 (m) 1475 (s), 1305 (m), 1257 (s), 1207 (m), 1149 (m), 1034 (m), 891 (m), 856 (m), 737 (m), 561 (w). ^**1**^**H NMR (300 MHz, 298 K, C**_**6**_**D**_**6**_**)**: δ 6.82 (s, 4H, C*H*_Ar_ - Mes), 6.47–6.44 (m, 2H, C_6_*H*_4_[N(Mes)]_2_), 5.57–5.54 (m, 2H, C_6_*H*_4_[N(Mes)]_2_), 3.58 (m, 8H, thf), 2.53 (s, 12H, C*H*_*3*_), 2.14 (s, 6H, C*H*_3_), 1.11 (m, 8H, thf). ^**13**^**C-{**^**1**^**H}-NMR (75 MHz, 298 K, C**_**6**_**D**_**6**_**):** δ 148.4, 141.2, 136.6, 131.7 (*C*_Ar_), 129.6, 125.5 110.2 (*C*H_Ar_), 73.9 (*C*H_2_, thf) 24.8, 21.1 (*C*H_3_), 19.2 (*C*H_2_, thf). Elemental analysis (%) Calcd for C_32_H_42_N_2_O_2_Cl_2_Ti (605.46): C, 63.48; H, 6.99; N, 4.63. Found: C, 64.02; H, 7.45; N, 4.95.

#### Synthesis of [{Ti(^Mes^PDA)(thf)}_2_(μ-Cl)_3_{Na}] (**3**)

A 50 mL Schlenk sample was charged in the glovebox with [TiCl_2_(^Mes^PDA)(thf)_2_] (0.3 g, 0.5 mmol) and sodium metal (0.025 g, 1.1 mmol) in 20 mL of tetrahydrofuran, and the suspension was allowed to stir overnight. Then, the reaction crude was filtered through a medium porosity glass frit and the solvent was removed under reduced pressure. The resulting solid was dissolved in the minimum amount of tetrahydrofuran and the solution was layered with benzene. This caused the precipitation of compound **3** which was isolated as a dark brown solid (0.217 g, 84%). IR (KBr, cm^–1^):  = 2968 (m), 2915 (m), 2858 (w), 1596 (m), 1481 (s), 1306 (m), 1257 (s), 1148 (m), 1036 (m), 857 (m), 741 (m), 561 (w). Elemental analysis (%) Calcd for C_56_H_68_N_4_O_2_Cl_3_Ti_2_Na (1054.25): C, 63.80; H, 6.50; N, 5.31. Found: C, 64.42; H, 7.44; N, 5.85. The effective magnetic moment of **3** was determined to be 1.88 μ_B_ (based on a unit formula of C_36_H_50_N_2_O_3_Cl_1_Ti_1_) on a [D_8_]-tetrahydrofuran solution. EPR (160 K, thf): *g*_1_ = 1.982, *g*_2_ = 1.960, and *g*_3_ = 1.887.

#### Synthesis of [Li(thf)_4_][(Ti^Mes^PDA)_2_(μ-η^6^:η^6^-C_6_H_6_)] (**4**)

A 100 mL Schlenk vessel was charged in the glovebox with compound **2** [TiCl_2_(^Mes^PDA)(thf)_2_] (0.36 g, 0.60 mmol) and cyclopentyllithium (0.12 g, 1.5 mmol) in 15 mL of benzene. The solution was allowed to stir for 6 h. The formed LiCl was removed upon filtration through a medium porosity glass frit, and all of the volatiles were removed under reduced pressure, affording compound **4** as a black solid (0.25 g, 72%). IR (KBr, cm^–1^):  = 3090 (w), 2953 (m), 2913 (m), 1594 (w), 1478 (s), 1260 (s), 1205(m), 1148 (m), 1031 (s), 878 (s), 739 (m). Elemental analysis (%) Calcd for C_70_H_90_N_4_O_4_Ti_2_Li (1154.16): C, 72.85; H, 7.86; N, 4.85. Found: C, 72.23; H, 7.48; N, 4.05. The effective magnetic moment of **4** was determined to be 1.78 μ_B_ (based on a unit formula of C_70_H_90_N_4_O_4_Ti_2_Li) on a [D_6_]-benzene solution. EPR (160 K, benzene): *g* = 1.977; EPR (160 K, solid state): *g* = 1.978.

#### General Method for Optimization of the Catalyst Conditions

In an argon-filled glovebox, phenylacetylene (0.03 g, 0.3 mmol), complex **4** (0.03–0.075 mmol), ferrocene (0.005 g, 0.03 mmol), and the deuterated solvent (1 mL) were charged into a vial. The mixture was stirred during the specified time (3–6 h) at room temperature. Then, the conversion of phenylacetylene to both isomers (1,3,5 and 1,2,4-triphenylbenzene) was determined by analyzing a sample by ^1^H NMR spectroscopy with ferrocene as the standard.

#### General Procedure for Catalytic Reactions

In an argon-filled glovebox, a 50 mL Schlenk was charged with **4** (0.075 mmol, 2.5 mol %), alkyne (3.0 mmol), in 5 mL of benzene. The reaction was stirred for 3 h. After that time, the reaction mixture was quenched at air and the volatiles were removed under vacuum. Then, the reaction crude was dissolved in the solvent mixture used as eluent. The final products were purified by silica-gel chromatography (see spectroscopical details of isolated products section in the Supporting Information for further details).

## Supporting Information Available

Spectroscopical details for compounds **2**–**4**, Evans method for compounds **3** and **4**, EPR spectroscopy, monitoring of H_2_ evolution over time for the synthesis of compound **4**, reaction monitoring for the synthesis of compound **4** by ^1^H NMR spectroscopy, kinetic and mechanistic studies, crystallographic data for compounds **2**–**4** and **8a**, quenching experiment for the cyclotrimerization of HCCSiMe_3_ catalyzed by compound **4**, ratio of isomers a and b determined by ^1^H NMR spectroscopy, spectroscopical details of isolated products and computational details (PDF)
