# Supplementary material for: A Mixed-Valence Ti(II)/Ti(III) Inverted Sandwich Compound as a Regioselective Catalyst for the Uncommon 1,3,5-Alkyne Cyclotrimerization
Source: Inorg Chem. 2024 May 1;63(19):8642–53. doi: 10.1021/acs.inorgchem.4c00149 (PMC11094787; doi:10.1021/acs.inorgchem.4c00149)
Supplement: Supplementary file 1 — ic4c00149_si_001.pdf [file ic4c00149_si_001.pdf]

# *A Mixed-Valence Ti(II)/Ti(III) Inverted Sandwich Compound as a Regioselective Catalyst for the uncommon 1,3,5-Alkyne Cyclotrimerization.*

*Elena Álvarez-Ruiz,*[a‡] *Ignacio Sancho,*[a‡] *Marta Navarro,*[b] *Israel Fernández,*[c] *Cristina Santamaría,*[a] *Alberto Hernán-Gómez,*[a]*

[a] Departamento de Química Orgánica y Química Inorgánica, Instituto de Investigación Química "*Andrés M. del Río*" (IQAR), Universidad de Alcalá, Campus Universitario, E-28805 Alcalá de Henares, Madrid, Spain.

[b] Departamento de Química Inorgánica, Orgánica y Bioquímica, Facultad de Ciencias y tecnologías Químicas, Universidad de Castilla-La Mancha, 13071-Ciudad Real, Spain.

[c] Departamento de Química Orgánica I, Facultad de Ciencias Químicas and Centro de Innovación en Química Avanzada (ORFEO-CINQA), Universidad Complutense de Madrid, 28040 Madrid, Spain

‡ E.A.-R. and I. S. contributed equally to this work.

Corresponding author:

*E-mail: alberto.hernan@uah.es

**Table of contents**


1. **Spectroscopical details for compounds 2-4** ..... 4

2. **Evans Method for compounds 3 and 4** ..... 7

3. **EPR Spectroscopy** ..... 9

4. **Monitoring of $H_2$ evolution over time for the synthesis of compound 4** ..... 11

5. **Reaction monitoring for the synthesis of compound 4 by $^1H$ NMR spectroscopy.** ..... 13

6. **Kinetic and mechanistic studies** ..... 13

7. **Crystallographic data for compounds 2-4 and 8a.** ..... 17

8. **Quenching Experiment for the Cyclotrimerization of $HCCSiMe_3$ Catalyzed by Compound 4 (15 mol%)** ..... 18

9. **Ratio of isomers a and b determined by $^1H$-NMR spectroscopy.** ..... 19

10. **Spectroscopical details of isolated products** ..... 24

11. **Computational Details.** ..... 38

## 1. Spectroscopical details for compounds 2-4

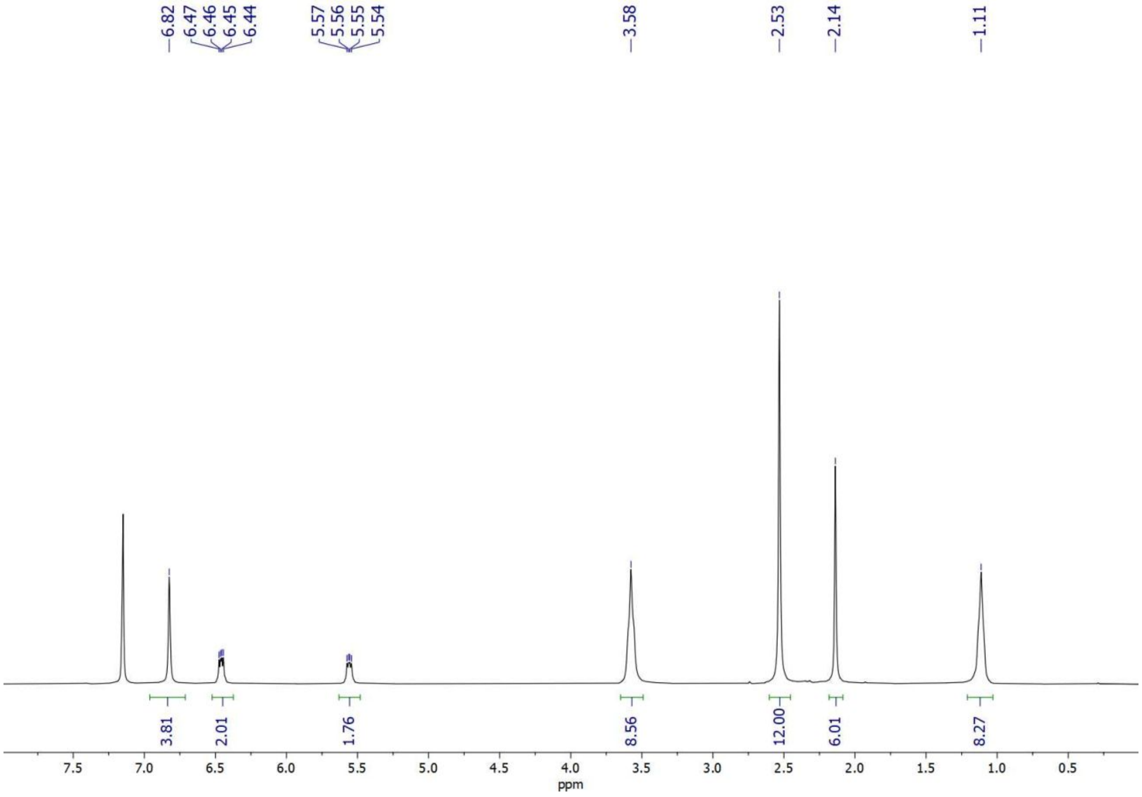


**Figure S1.** $^{1}H$-NMR spectrum ($C_6D_6$ ,300MHz, 298K) for complex $[TiCl_2(^{Mes}PDA)_2(thf)_2]$ (**2**)

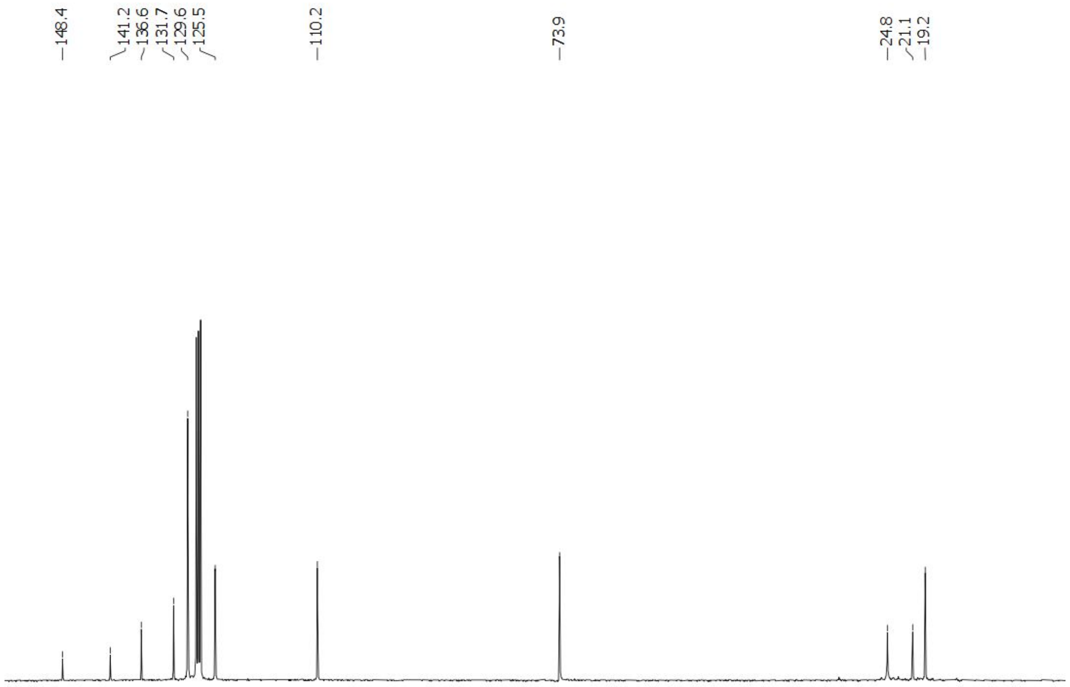


**Figure S2.** $^{13}C$-{$^{1}H$}-NMR spectrum ($C_6D_6$, 75MHz, 298K) for complex $[TiCl_2(^{Mes}PDA)_2(thf)_2]$ (**2**)

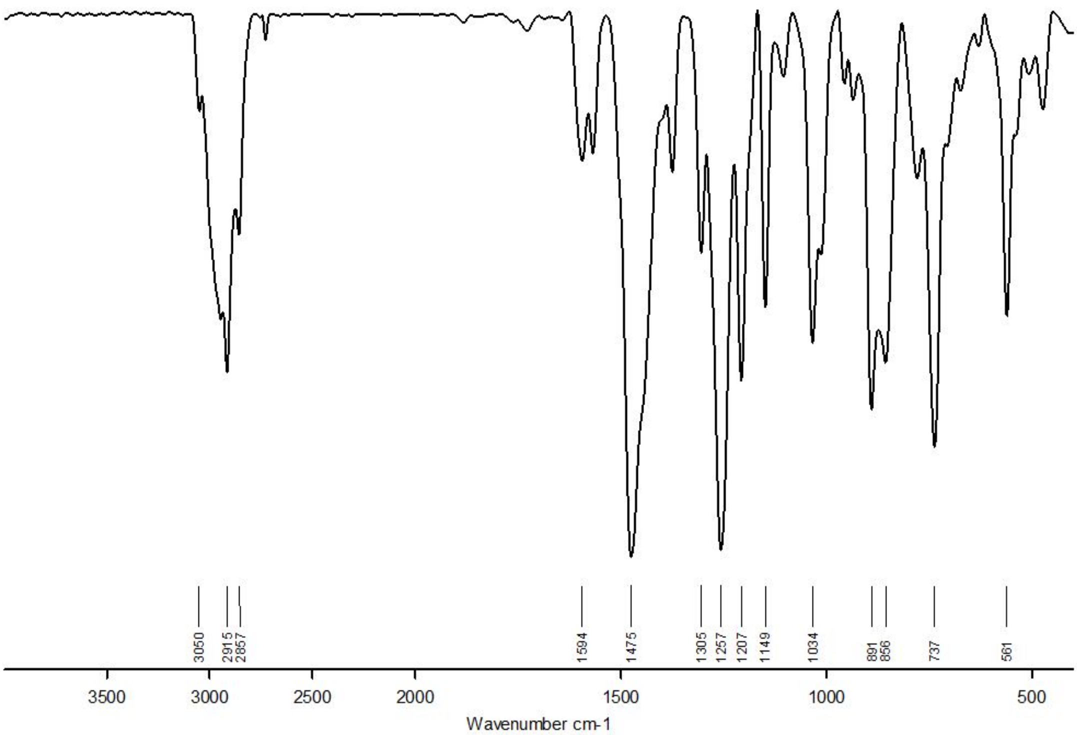


**Figure S3.** FTIR spectrum for complex [$TiCl_2$($^{Mes}$PDA)$_2$(thf)$_2$] (**2**)

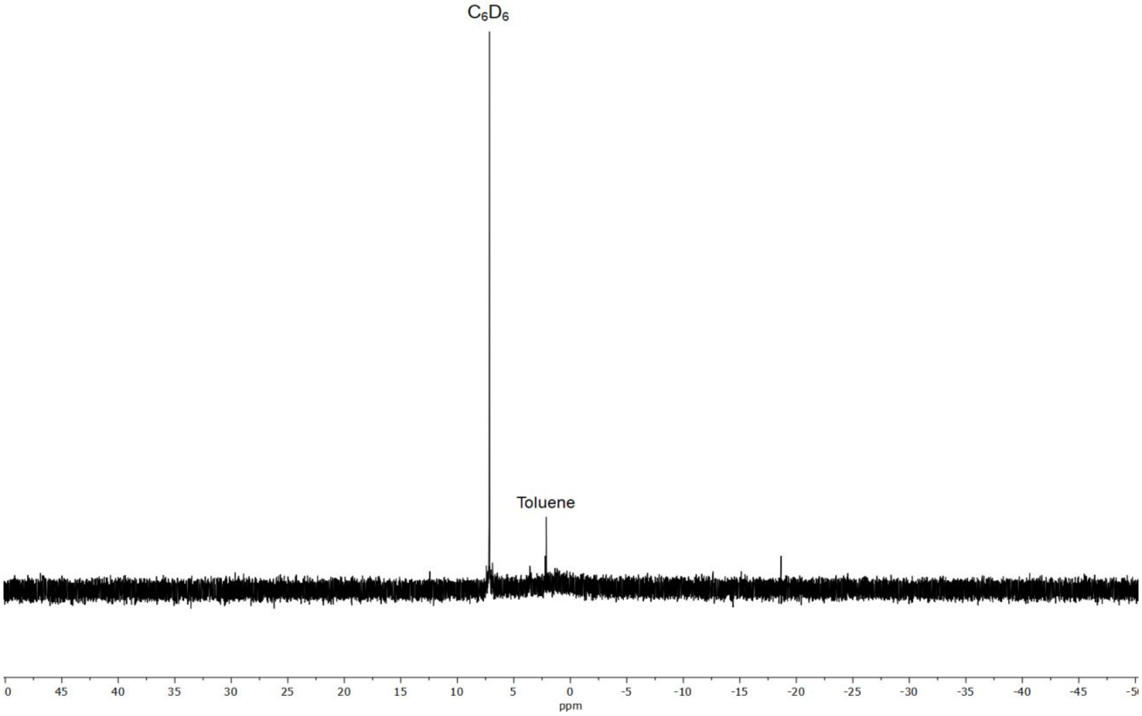


**Figure S4.** $^1$H-NMR spectrum ($C_6D_6$, 300MHz, 298K) for complex [{Ti($^{Mes}$PDA)(thf)}$_2$(μ-Cl)$_3${Na}] (**3**)

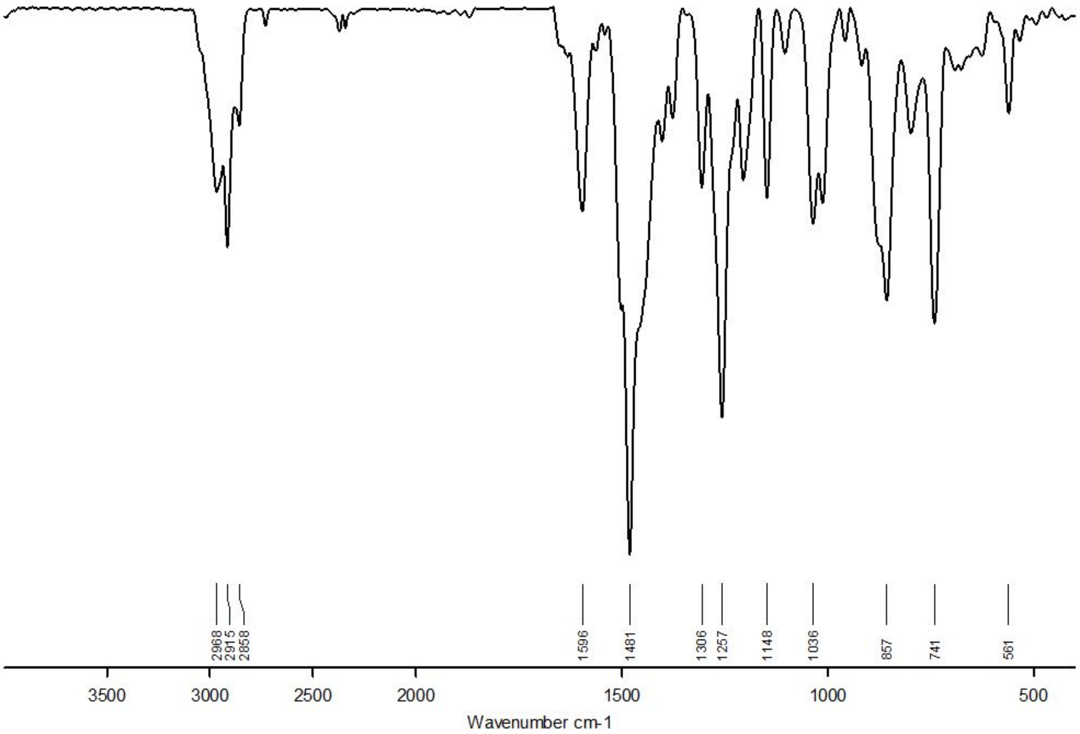


**Figure S5.** FTIR spectrum for complex [{Ti($^{Mes}$PDA)(thf)}$_2$(μ-Cl)$_3${Na}] (**3**)

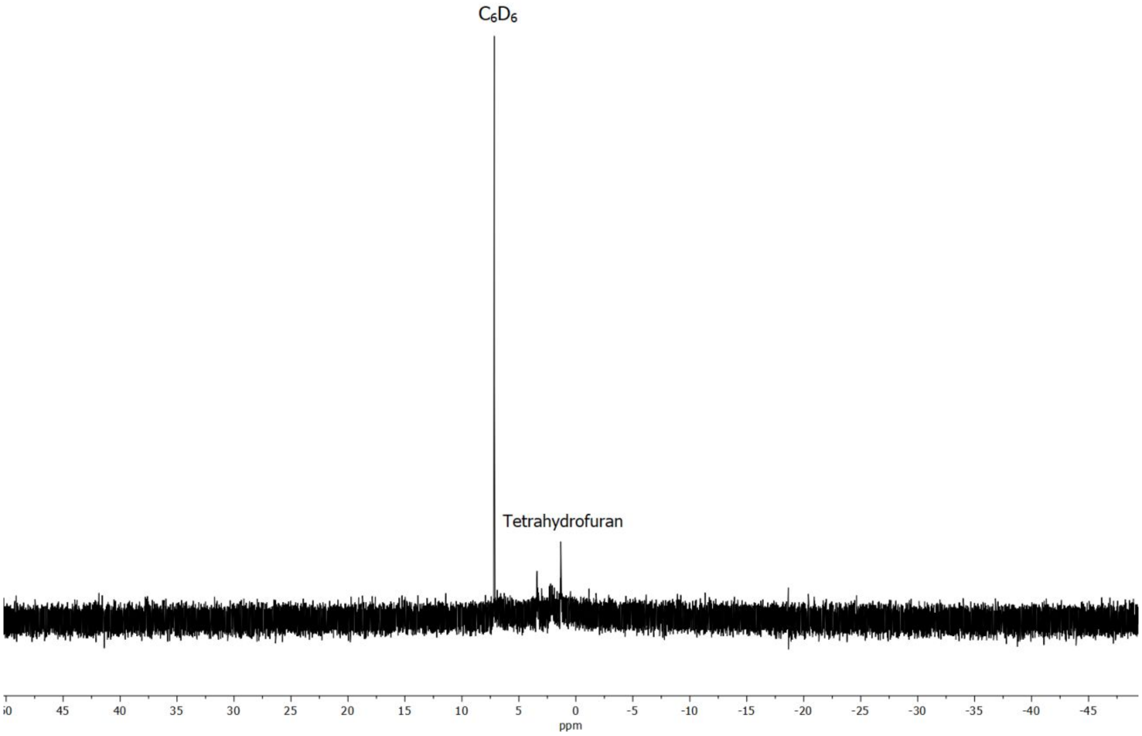


**Figure S6.** $^1$H-NMR spectrum ($C_6D_6$, 300MHz, 298K) for complex [Li(thf)$_4$][(Ti$^{Mes}$PDA)$_2$(μ-η$^6$:η$^6$-$C_6H_6$)] (**4**)

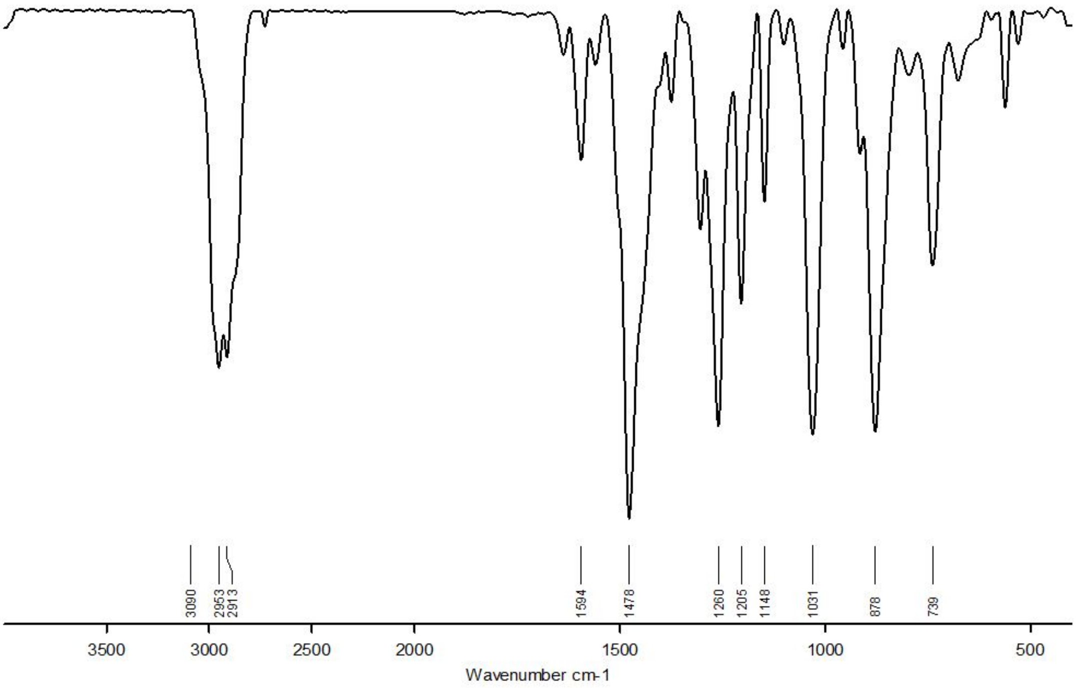


**Figure S7.** FTIR spectrum for complex $[Li(thf)_4][(Ti^{Mes}PDA)_2(\mu\text{-}\eta^6\text{:}\eta^6\text{-}C_6H_6)]$ (**4**)

## 2. Evans Method for compounds 3 and 4

The effective magnetic moments were determined using the Evans[1] method via NMR at 293 K, employing a 300 MHz instrument with a field strength of 7.05 Tesla. This technique involves measuring the frequency shift of the $^1H$ NMR resonance signal of cyclohexane, which serves as a standard. The shift occurs due to the additional magnetic field generated by the presence of the paramagnetic species. For this measurement, a reference coaxial NMR tube containing an 90:10 mixture of deuterated solvent:cyclohexane is used alongside the outer sample tube, which contains the same solvent mixture and the paramagnetic compound under investigation.

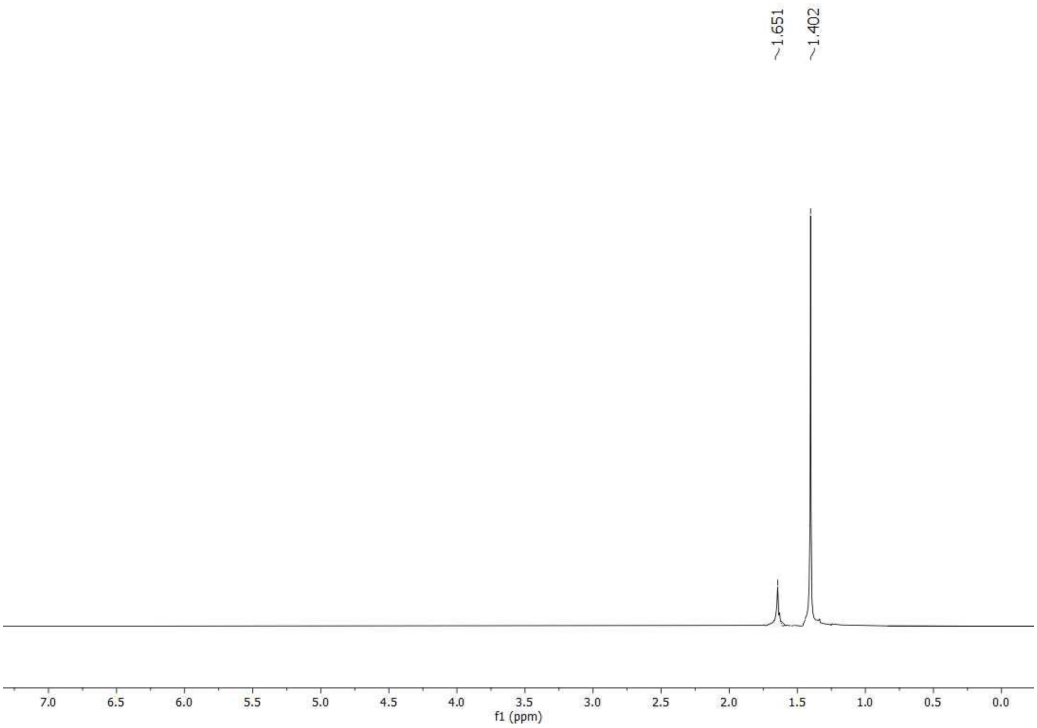


**Figure S8.** $^1$H NMR spectrum obtained using the Evans method for a NMR tube setup containing a [$d_8$-thf]:cyclohexane (90:10) solution of compound [{Ti($^{Mes}$PDA)(thf)}$_2$(μ-Cl)$_3${Na}] (**3**) (0.04 M), and a coaxial tube with a mixture of [$d_8$-thf]:cyclohexane (90:10). The difference between cyclohexane peaks is 74.7 Hz.

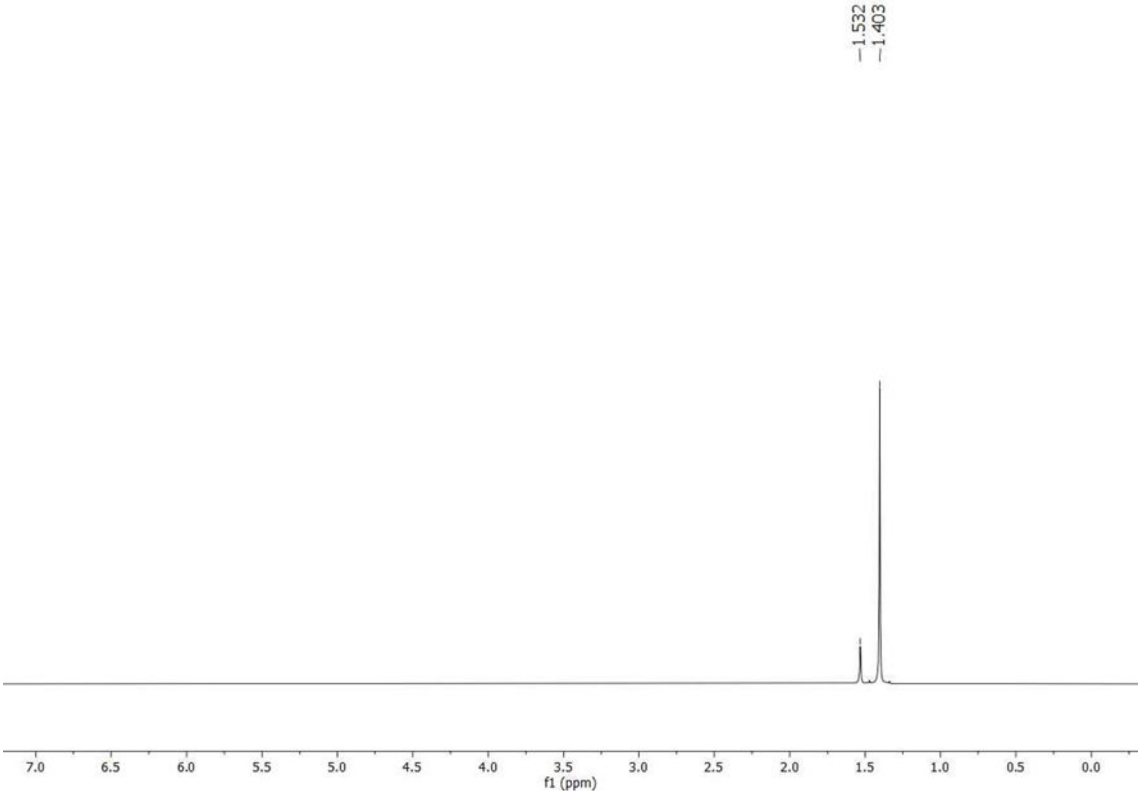


**Figure S9.** $^1$H NMR spectrum obtained using the Evans method for a NMR tube setup containing a $C_6D_6$:cyclohexane (90:10) solution of compound [Li(thf)$_4$][(Ti$^{Mes}$PDA)$_2$(μ-η$^6$:η$^6$-$C_6H_6$)] (**4**) (0.023 M), and a coaxial tube with a mixture of $C_6D_6$:cyclohexane (90:10). The difference between cyclohexane peaks is 38.7 Hz.

## 3. EPR Spectroscopy

A 5 mM frozen thf solution of compound **3** was registered in a Bruker EMX spectrometer, while compound **4** was registered in a Bruker Magnettech ESR5000 spectrometer in solid state and in a 5 mM benzene solution at 160 K.

**Experimental parameters**:

Compound **3**: Temperature = 160 K; MW power = 0.2 mW; modulation frequency = 100 kHz; modulation amplitude = 4.0 G.

Compound **4**: Temperature = 160 K; MW power = 20 mW; modulation frequency = 100 kHz; modulation amplitude = 0.5 G.

**Simulation parameters** (performed using the Easyspin toolbox in Matlab):[2]

Compound **3** (thf solution): g = [1.982, 1.960, 1.887]; lw = [0.126, 1.483]; gStrain = [0.0051, 0.009, 0.02] (Figure S10a).

Compound **4** (Benzene solution): g = [1.977]; lw = [3, 2.7819] (Figure S10b).

Compound **4** (solid state): g = [1.978]; lw = [2.1678, 2.6182] (Figure S10c).

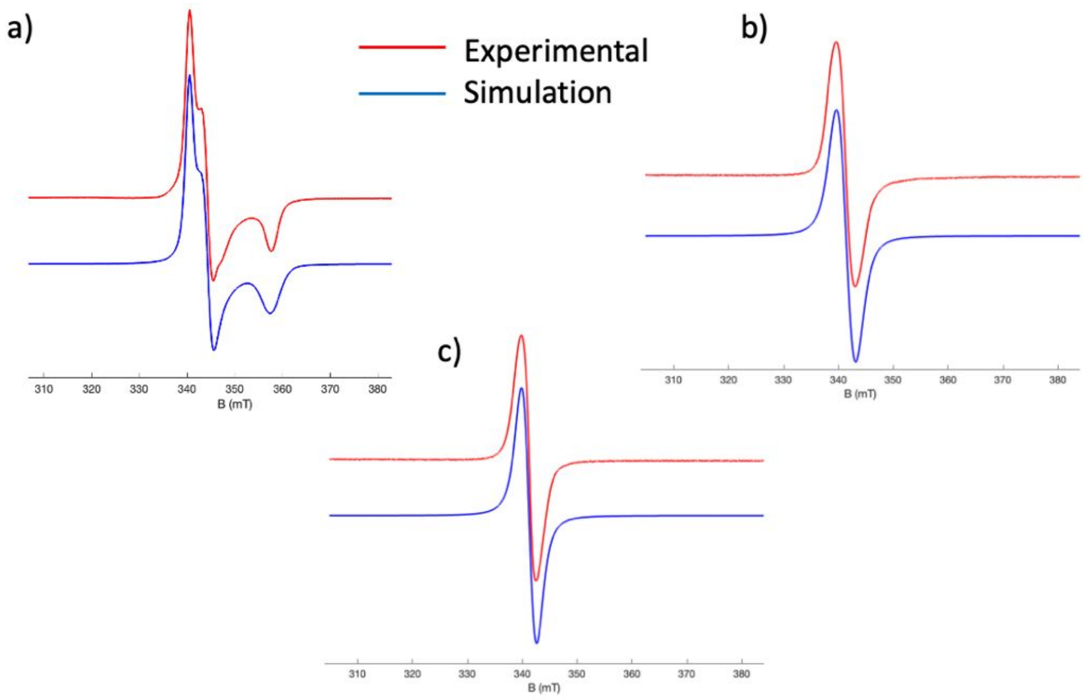


**Figure S10**. Low-temperature (160 K) EPR spectra for compound **3** a) in thf solution and compound **4** in b) benzene solution and c) solid state.

The spin quantification, and consequently, the concentration of a 20 mM solution of compound **4**, was registered using the SpinCount feature, an extension of the ESRStudio software by Bruker. The experimental findings yielded a spin count of 1.06 x $10^{18}$ and a concentration of 17.2 mM. The slight discrepancy from the theoretical concentration of 20 mM may be attributed to the extreme air- and moisture-sensitivity of compound **4**, which likely undergoes partial hydrolysis upon dissolution in benzene. Specifically, the 2.8 mM difference corresponds to a 0.52 mg of compound **4**. Although this solvent is always degassed and dried by distillation over a sodium/potassium alloy, the presence of trace amounts of water and oxygen cannot be completely excluded.

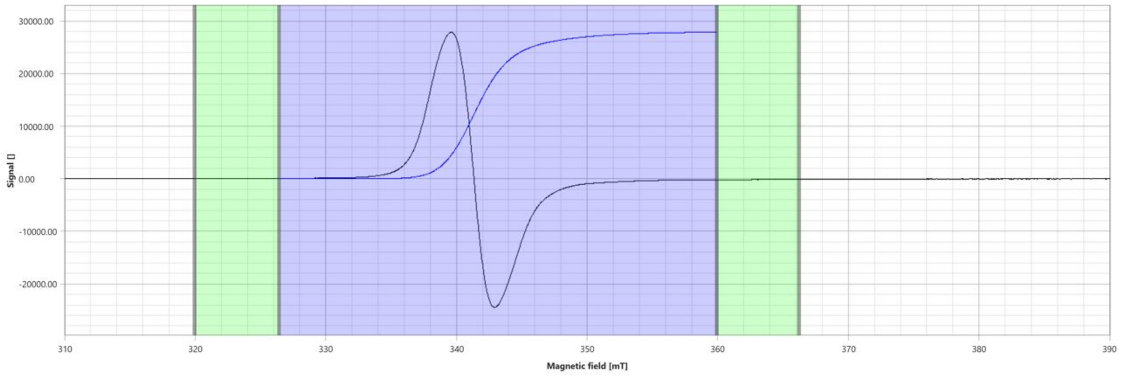


**Figure S11**. Integral used for spin quantification of a 20 mM benzene solution of compound **4** at (160 K) by EPR.

Additionally, we monitored the reaction mixture of phenylacetylene with 2.5 mol% compound **4** (5 mM) in frozen benzene (160 K) using a Bruker Magnettech ESR5000 spectrometer during the catalytic transformation. The EPR spectra, showing a rhombic symmetry that averages to an isotropic g value $g_{iso}$ = 1.977 similar to compound **4**, revealed a decrease in signal intensity over time, as shown in Figure S12. Similarly, EPR analysis of 2-ethynylnaphthalene catalyzed by 2.5 mol% of compound **4** at 160 K displayed a broad signal at a slightly shifted g value (1.958) (Figure S13). These results support the proposal that a mixed valence Ti(II)/Ti(III) inverted sandwich compound similar to **4** is retained during the catalysis, with the formed tris(aryl)benzene acting as the bridging moiety. The decrease of the EPR signal over time suggests a possible decomposition of the catalyst.

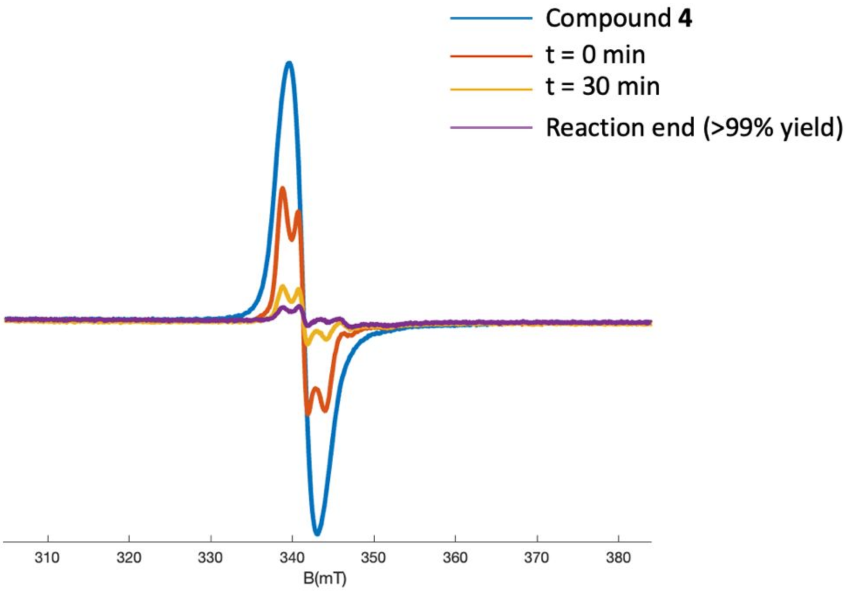


**Figure S12**. EPR comparison for the spectra acquired for compound **4** (blue line), catalytic reaction of phenylacetylene and 2.5mol% of **4** at t=0, (red line), t=30 min (yellow line) and at the reaction end (>99 yield) (purple line).

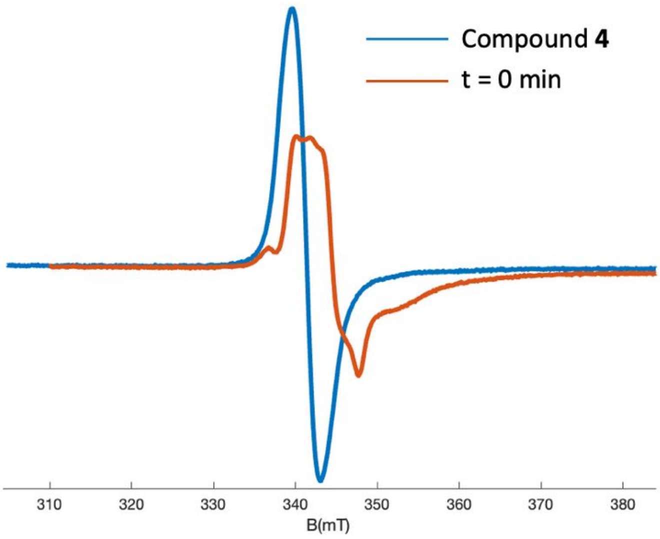


**Figure S13**. EPR comparison for the spectra acquired for compound **4** (blue line) and the catalytic reaction of 2-ethynylnaphthalene and 2.5mol% of **4** at t=0 (red line).

## 4. Monitoring of $H_2$ evolution over time for the synthesis of compound 4

$$\textbf{(2)} + 2.5\ Li(C_5H_9) + C_6H_6 \longrightarrow 0.5\ (\textbf{4}) + C_5H_{10} + 1.5\ C_5H_8 + 0.25\ H_2 \quad \text{Eq. 1}$$

The hydrogen production during the formation of complex **4** was monitored through the Man on the Moon X102 Kit (https://www.manonthemoontech.com/index.html),

monitoring the variation of pressure inside a closed glass reactor. The reaction flask is connected to a switchable 3-way valve which can be switched between two positions. One of the positions connects the reactor vessel to the exterior, while the other connects the flask to the pressure transducer.

A round-bottom 20 mL flask was charged in the glovebox with complex **2** (0.08 mmol, 0.05 g) and 2 mL of benzene. Then, the flask was connected to the switchable three-way valve through a Torion screw. While stirring the reaction mixture at room temperature (298 K), the valve was switched to the pressure transducer which is connected via wireless to the software. Once the pressure measurement was stabilized, cyclopentyl lithium (0.2 mmol, 0.015 g) was added, and was recorded until constant pressure in the micro-reactor was reached (6 h). The number of $H_2$ equivalents released was calculated assuming ideality for hydrogen gas and applying the PV = nRT.

The calculated number of $H_2$ mmol is 0.0185 (0.0228 bar) which is consistent with the release of 0.25 equivalents (0.020 mmol) of $H_2$ in the transformation of 0.08 mmol of compound **2** to **4**, according to equation 1.

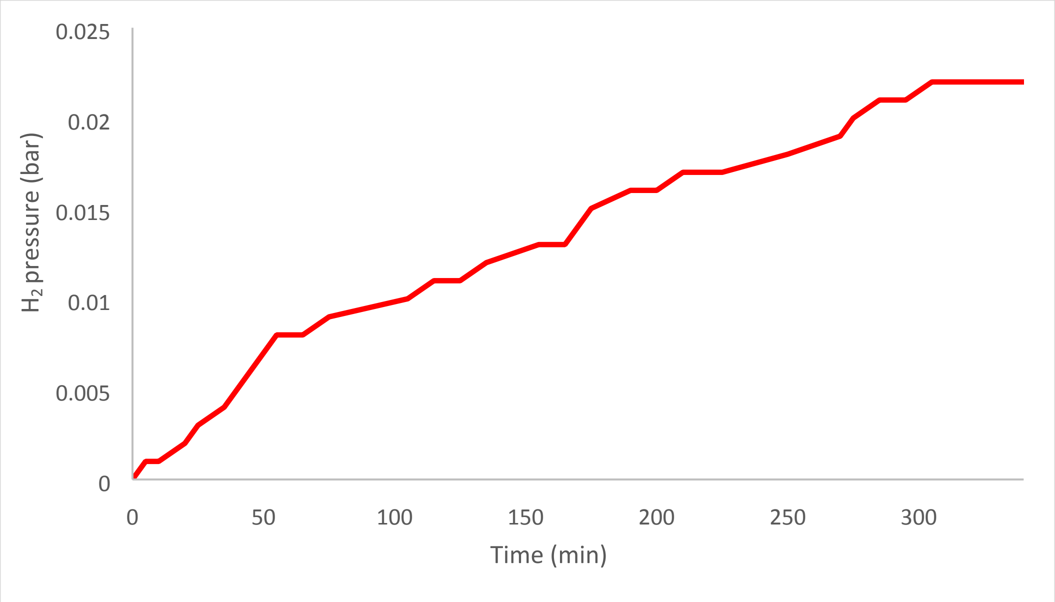


**Figure S14.** Pressure Monitoring for the chemical reduction of compound **2**.

## 5. Reaction monitoring for the synthesis of compound 4 by $^1$H NMR spectroscopy.

Reaction between compound **2** (0.020 g, 0.033 mmol) and cyclopentyl lithium (0.006 g, 0.083 mmol) was monitored in $C_6D_6$ at 298K using *in situ* $^1$H-NMR spectroscopy and a coaxial tube containing a $C_6D_6$ solution of ferrocene (0.0014 mmol) as internal standard. Analysis of the relative integrals disclose the formation of 1.45 equivalents of cyclopentene (0.048 mmol) and 1.21 equivalents of cyclopentane (0.040 mmol), which corresponds to a slight deviation from the theoretical values of 1.5 equivalents for cyclopentene and 1 equivalent for cyclopentane.

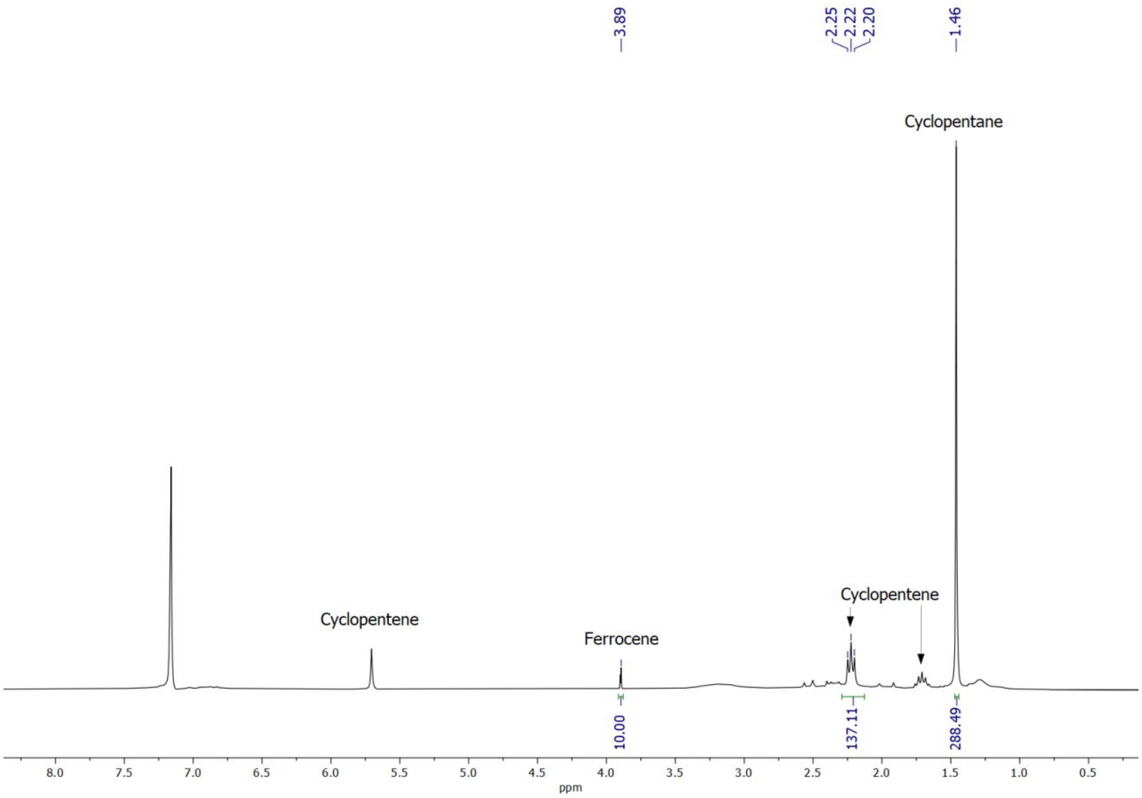


**Figure S15**. $^1$H-NMR spectrum ($C_6D_6$, 300MHz, 298K) for the synthesis of complex $[Li(thf)_4][(Ti^{Mes}PDA)_2(\mu\text{-}\eta^6\text{:}\eta^6\text{-}C_6H_6)]$ (**4**) using ferrocene as internal standard in a coaxial tube

## 6. Kinetic and mechanistic studies

We employed the initial rates and integration methodologies to determine the orders of the catalyst and the alkyne, respectively.

For the initial rates, cyclotrimerization of trimethylsilylacetylene was monitored in $C_6D_6$ at 298K using *in situ* NMR spectroscopy by following the disappearance of the resonance assigned to the Si*Me₃* of the terminal alkyne. The percentage of conversion was restricted to 7.5% in order to calculate the initial rate ($r_0$) of the reaction. The concentration range of the catalyst studied was between 0.00607 M and 0.0204 M while keeping constant the concentration of alkyne to 0.4 M. Under these conditions, the following equations apply:

$$r_0 = -\Delta[\text{Alkyne}]/\Delta t = k[\text{Catalyst}]^n[\text{Alkyne}]^m \qquad \text{Eq. 2}$$

$$[\text{Alkyne}] = [\text{Alkyne}]_0 - r_0 t \qquad \text{Eq. 3}$$

$$r_0 = k[\text{Catalyst}]^n[\text{Alkyne}]^m \qquad \text{Eq. 4}$$

For a series of experiments where the catalyst concentration is modified while the alkyne concentration is kept constant: $r_0 = k'[\text{Catalyst}]^n$ ($k' = k[\text{alkyne}]^m$) Eq. 5

According to these equations, the acquired data were plotted as molar concentration of the starting material versus time yielding straight lines (Eq. 3, Figure S16), which were fitted by conventional linear regression ($r^2 > 0.97$) and $r_0$ values were obtained from the corresponding slopes. Catalyst order was determined by plotting $r_0$ versus the corresponding concentrations (Eq 5. n =1, Figure S17)

For the alkyne, the reaction order was obtained by the integration method. This method involves monitoring the variation of concentration of alkyne versus time (min) to the end of the reaction and fitting the data to the appropriate integrated equation. Under these conditions, the following equations apply:

$$\upsilon = -d[\text{alkyne}]/dt = k'[\text{Alkyne}]^n, \text{ where } k' = k[\text{catalyst}] \qquad \text{Eq. 6}$$

The integrated equation for n = 2 is: $(1/[\text{Alkyne}])-(1/[\text{Alkyne}]_0) = -k't$ Eq. 7

We monitored the cyclotrimerization of trimethylsilylacetylene (0.4 M) using a 5% catalyst loading until 85% yield. The acquired data provides the best fit for a second-order integration equation (Eq. 7, Figure S18).

The rate law derived from all these kinetic experiments is the following:

$$v = k\,[\mathbf{4}]^1[\text{alkyne}]^2$$

**Table S1.** Initial rates for different concentrations of **4**.

| [**4**] (M) | $r_0$ ($Ms^{-1}$) |
|---|---|
| 0.00607 | $1.63 \times 10^{-5}$ |
| 0.01020 | $3.20 \times 10^{-5}$ |
| 0.01527 | $4.36 \times 10^{-5}$ |
| 0.02040 | $6.30 \times 10^{-5}$ |

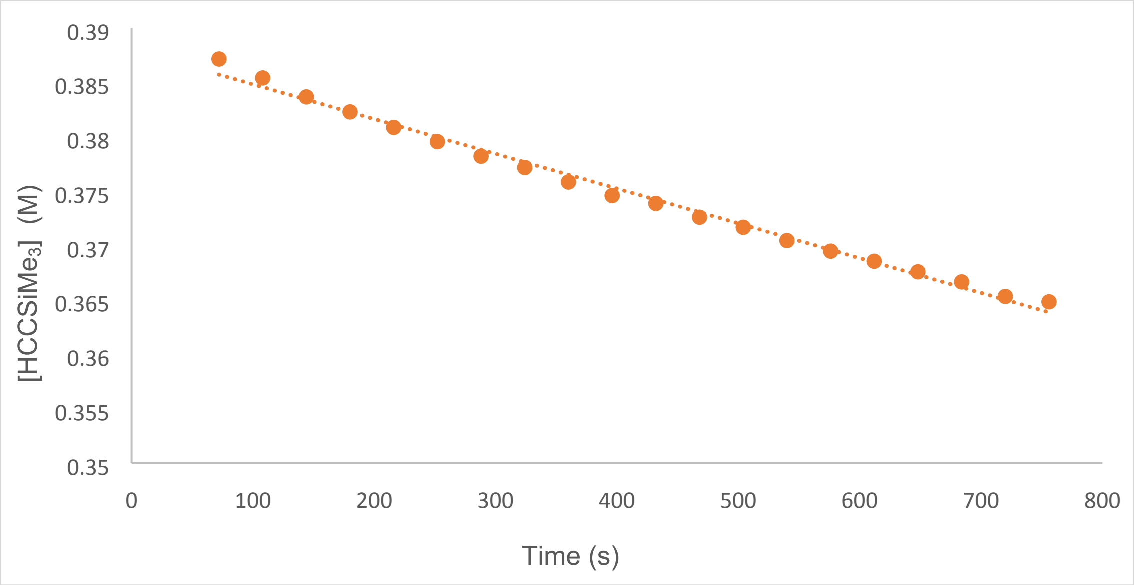


**Figure S16.** Example of the one of the plots of concentration [$HCCSiMe_3$] (M) vs time for the initial rates methodology. The reaction monitored is the cyclotrimerization of trimethylsilylacetylene with 2.5% catalyst loading of catalyst **4** (0.01020M) in [$d_6$]-benzene at 298 K.

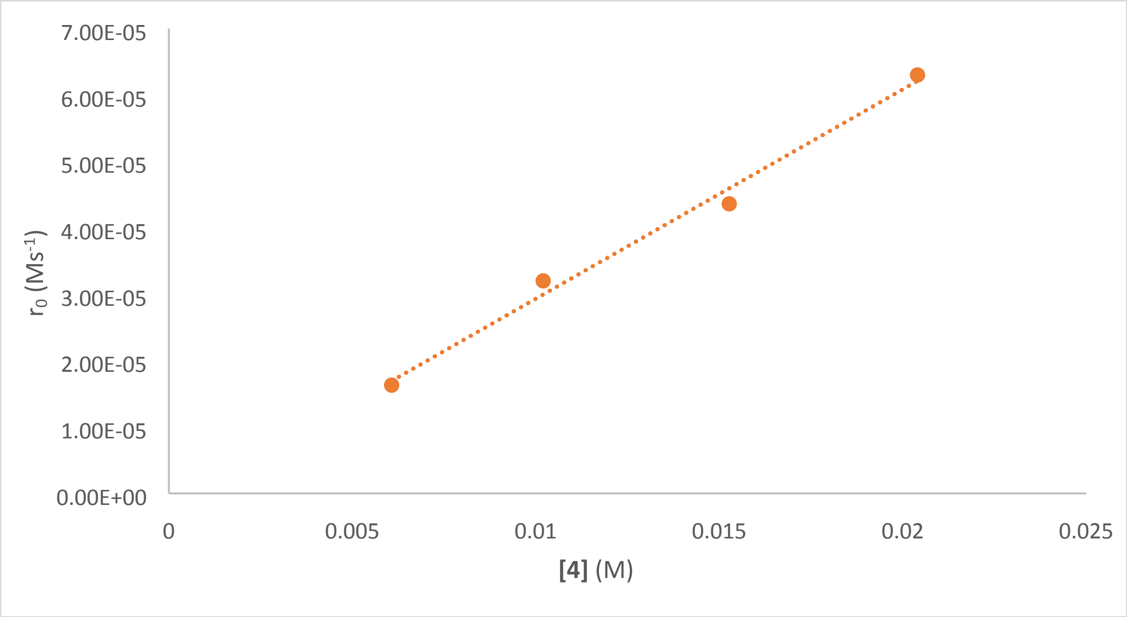


**Figure S17.** Plot of $r_0$ for the cyclotrimerization reaction of trimethylsilylacetylene (0.40 M) at different catalyst concentrations vs [**4**](M).

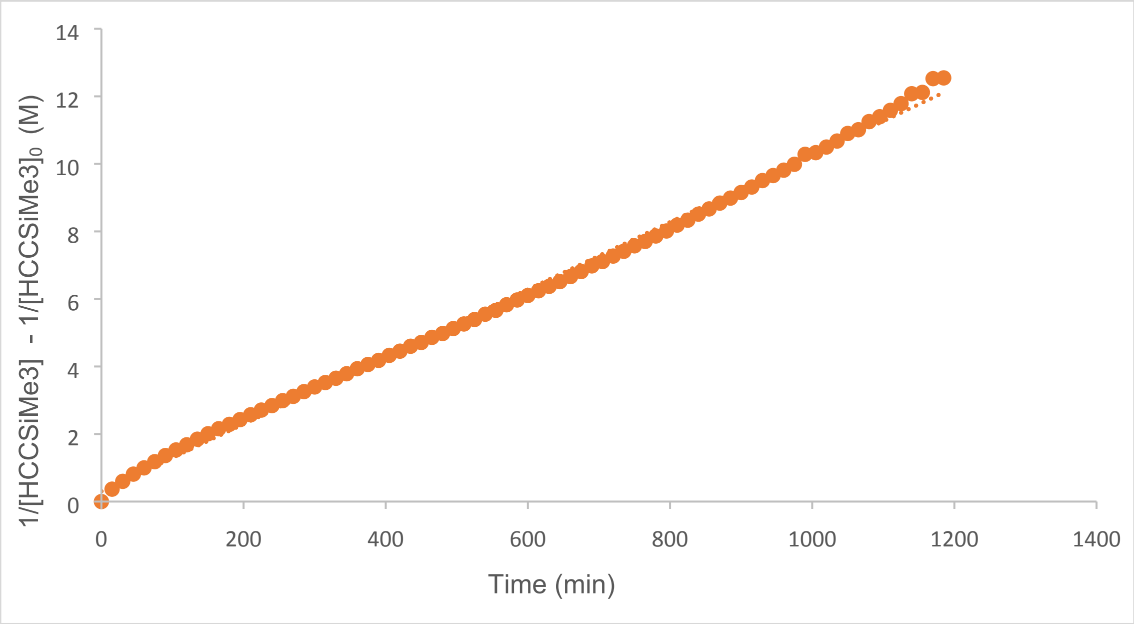


**Figure S18.** Plot of concentration 1/[$HCCSiMe_3$] – 1/[$HCCSiMe_3$]$_0$ (M) vs time (min) for the cyclotrimerization reaction of trimethylsilylacetylene (0.40 M) with 5% rate of catalyst **4** (0.020M) in [$d_6$]-benzene at 298 K.

## 7. Crystallographic data for compounds 2-4 and 8a.

**Table S2**. Selected crystallographic and refinement parameters.

| | **2** | **3** | **4** | **8a** |
|---|---|---|---|---|
| Empirical formula | C44H54Cl2N2O2Ti | C62H74Cl3N4NaO2Ti2 | C70H90LiN4O4Ti2 | C69H81O6 |
| Molecular Weight | 761.69 | 1132.39 | 1154.19 | 1006.33 |
| Temperature (K) | 150 | 200 | 150 | 150 |
| Wavelength (Å) | 0.71073 | 0.71073 | 0.71073 | 0.71073 |
| Crystal System | monoclinic | triclinic | monoclinic | monoclinic |
| Space Group | $P2_1/c$ | P-1 | $P2_1/c$ | $P2_1/n$ |
| a (Å) | 20.6394(12) | 13.4826(8) | 14.6703(6) | 26.744(3) |
| b (Å) | 9.7096(5) | 14.5121(12) | 13.1126(5) | 7.4688(6) |
| c (Å) | 16.5660(11) | 17.0881(11) | 33.4709(14) | 33.289(3) |
| $\alpha$ (º) | 90 | 105.902(7) | 90 | 90 |
| $\beta$ (º) | 97.049(3) | 103.590(4) | 94.585(2) | 110.412(4) |
| $\chi$ (º) | 90 | 105.740(8) | 90 | 90 |
| Cell Volume ($Å^3$) | 3294.7(3) | 2917.1(4) | 6418.1(4) | 6232.0(10) |
| Z | 4 | 2 | 4 | 4 |
| $\rho$ Calc ($g \cdot cm^{-3}$) | 1.536 | 1.289 | 1.194 | 1.073 |
| $\mu$ ($mm^{-1}$) | 0.469 | 0.464 | 0.299 | 0.067 |
| *F* (000) | 1616 | 1192 | 2468 | 2172 |
| 2θ max (º) | 55.28 | 55 | 55.096 | 52.742 |
| Index Ranges | $-26 \leq h \leq 26$<br>$-12 \leq k \leq 11$<br>$-21 \leq h \leq 21$ | $-17 \leq h \leq 17$<br>$-18 \leq k \leq 18$<br>$-22 \leq h \leq 22$ | $-19 \leq h \leq 19$<br>$-17 \leq k \leq 16$<br>$-43 \leq h \leq 43$ | $-33 \leq h \leq 33$<br>$-9 \leq k \leq 7$<br>$-41 \leq h \leq 41$ |
| Reflections collected | 73523 | 61520 | 136333 | 120284 |
| Reflections Unique | 7573 | 13292 | 14664 | 12722 |
| Reflections obs. | 6406 | 7625 | 11980 | 8593 |
| $R_{int}$ | 0.0630 | 0.1026 | 0.0533 | 0.0846 |
| No. Parameters | 374 | 708 | 782 | 601 |
| Goodnes of Fit on $F^2$ (GOF) | 1.057 | 1.084 | 1.077 | 1.044 |
| Final *R* indices [$I > 2s(I)$] | 0.0559 | 0.0509 | 0.0623 | 0.0544 |
| *R* indices (all data) | 0.1663 | 0.1295 | 0.1599 | 0.1700 |
| Largest diff. peak and hole (e $Å^{-3}$) | 0.731, -0.4 | 0.479, -0.494 | 0.435, -0.398 | 0.200, -0.183 |

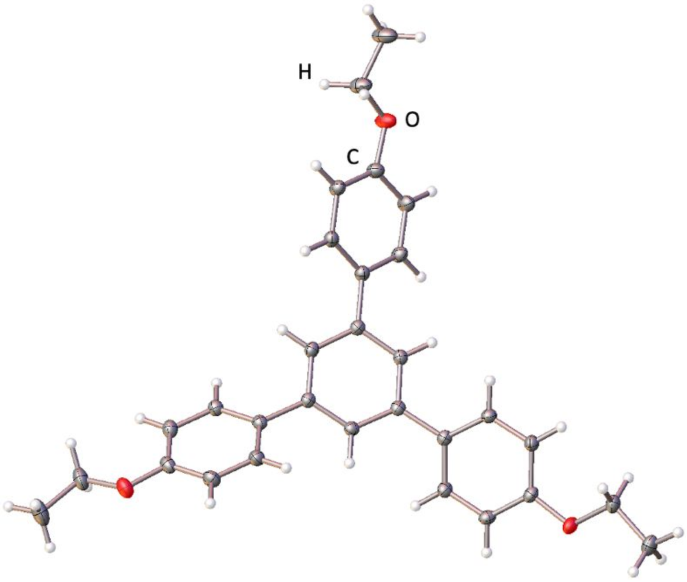


**Figure S19**. Solid state structure of compound **8a** with ellipsoids at 30% of probability.

## 8. Quenching Experiment for the Cyclotrimerization of $HCCSiMe_3$ Catalyzed by Compound 4 (15 mol%)

A $C_6D_6$ solution containing trimethylsilylacetylene and 15 mol% of catalyst **4** was monitored in situ via $^1H$ NMR spectroscopy. Upon reaching 20% conversion, the reaction was intentionally quenched by exposure to air. Subsequent $^1H$ NMR analysis of the crude mixture revealed only the presence of unreacted trimethylsilylacetylene, the free $^{Mes}PDAH_2$ ligand, and the final trisaryl product (Figure S20)

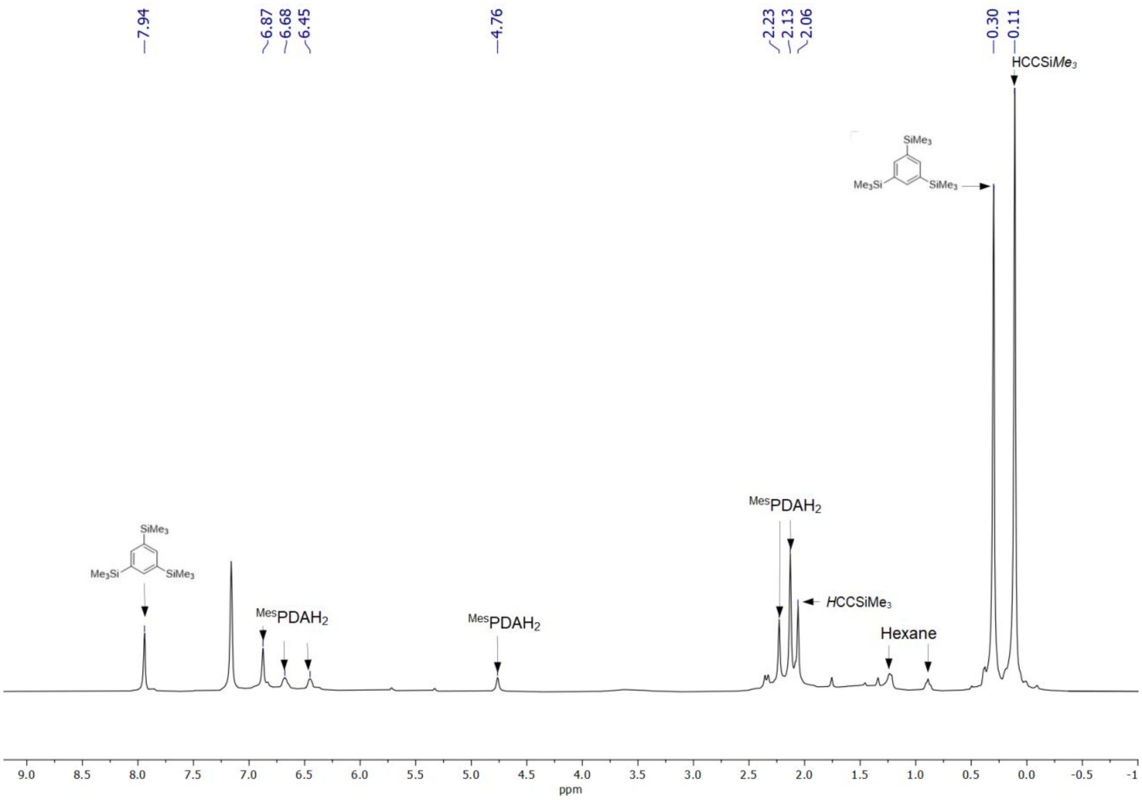


**Figure S20.** $^{1}$H-NMR spectrum ($C_6D_6$, 300MHz, 298K) for the hydrolysis of the cyclotrimerization reaction of trimethylsilylacetylene with 15% rate of catalyst **4** in [$d_6$]-benzene at 298 K.

## 9. Ratio of isomers a and b determined by $^{1}$H-NMR spectroscopy.

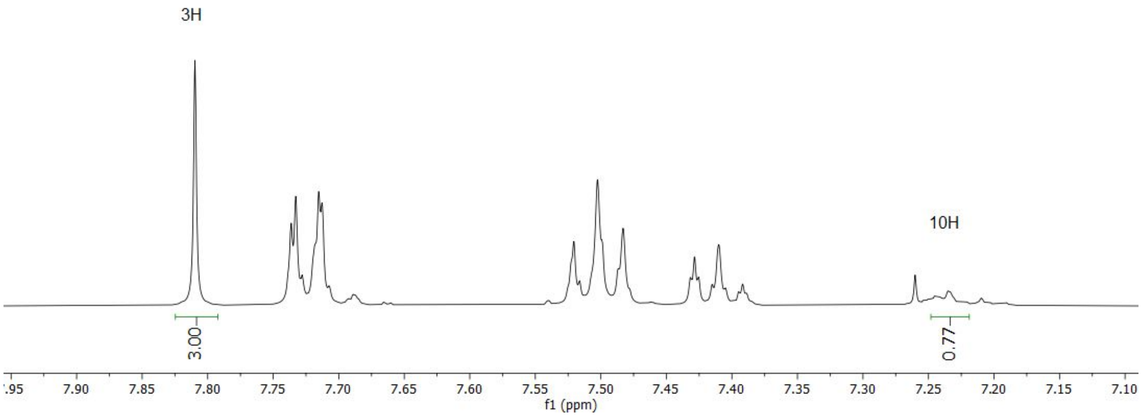


**Figure S21.** $^{1}$H-NMR spectrum ($CDCl_3$, 300MHz, 298K) for determination of ratio of isomers of compound (**5**). Resonance with integral value of 3 (3 H) corresponds to isomer 1, 3, 5. Resonance with integral value of 0.77 (10 H) corresponds to isomer 1, 2, 4.

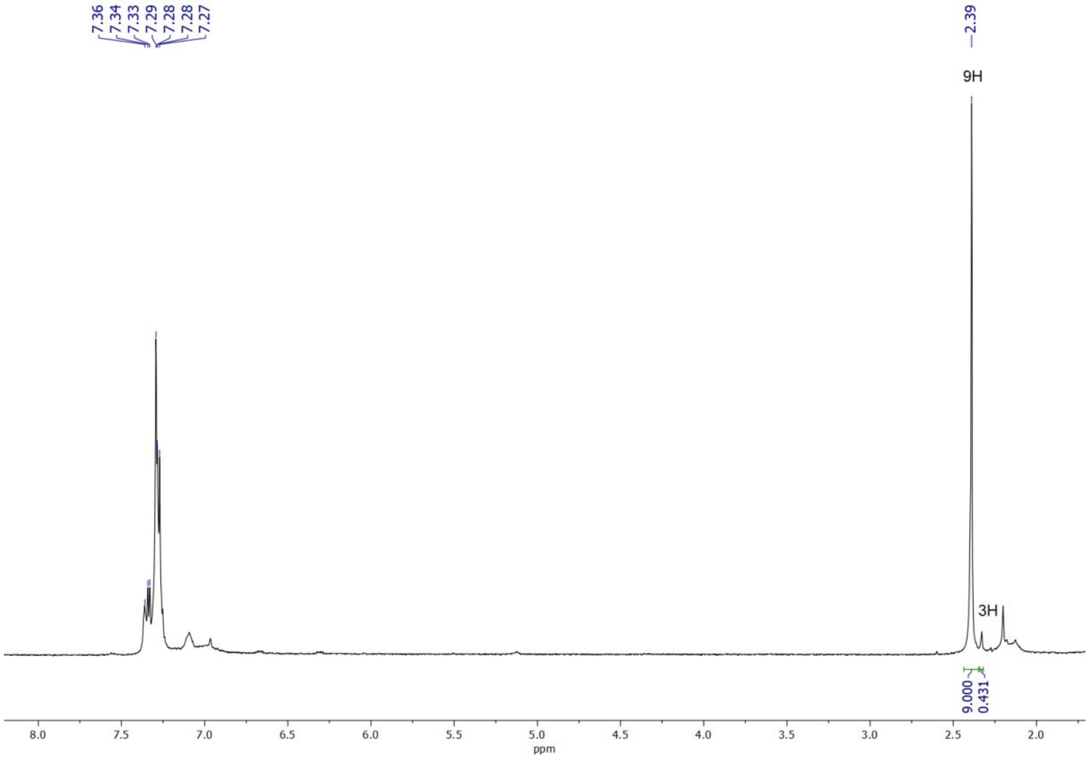


**Figure S22.** $^{1}H$-NMR spectrum ($CDCl_3$, 300MHz, 298K) for determination of ratio of isomers of compound (**6**). Resonance with integral value of 9 (9 H) corresponds to isomer 1, 3, 5. Resonance with integral value of 0.43 (3 H) corresponds to isomer 1, 2, 4.

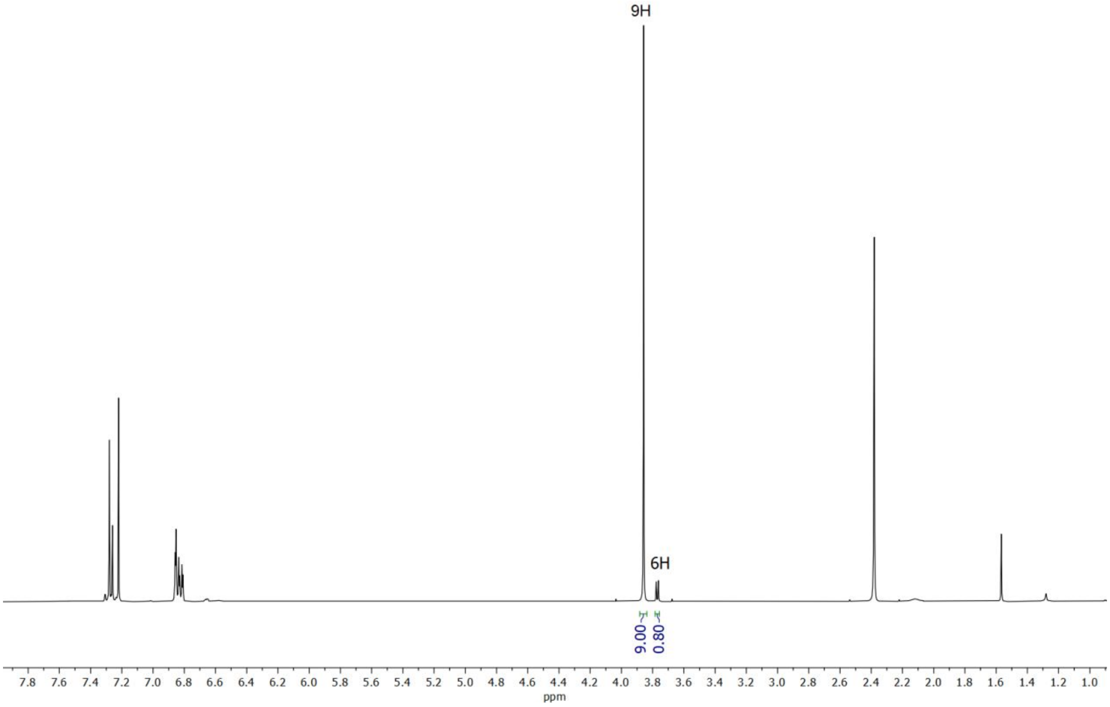


**Figure S23.** $^{1}H$-NMR spectrum ($CDCl_3$, 400MHz, 298K) for determination of ratio of isomers of compound (**7**). Resonance with integral value of 9 (9 H) corresponds to isomer 1, 3, 5. Resonance with integral value of 0.8 (6 H) corresponds to isomer 1, 2, 4.

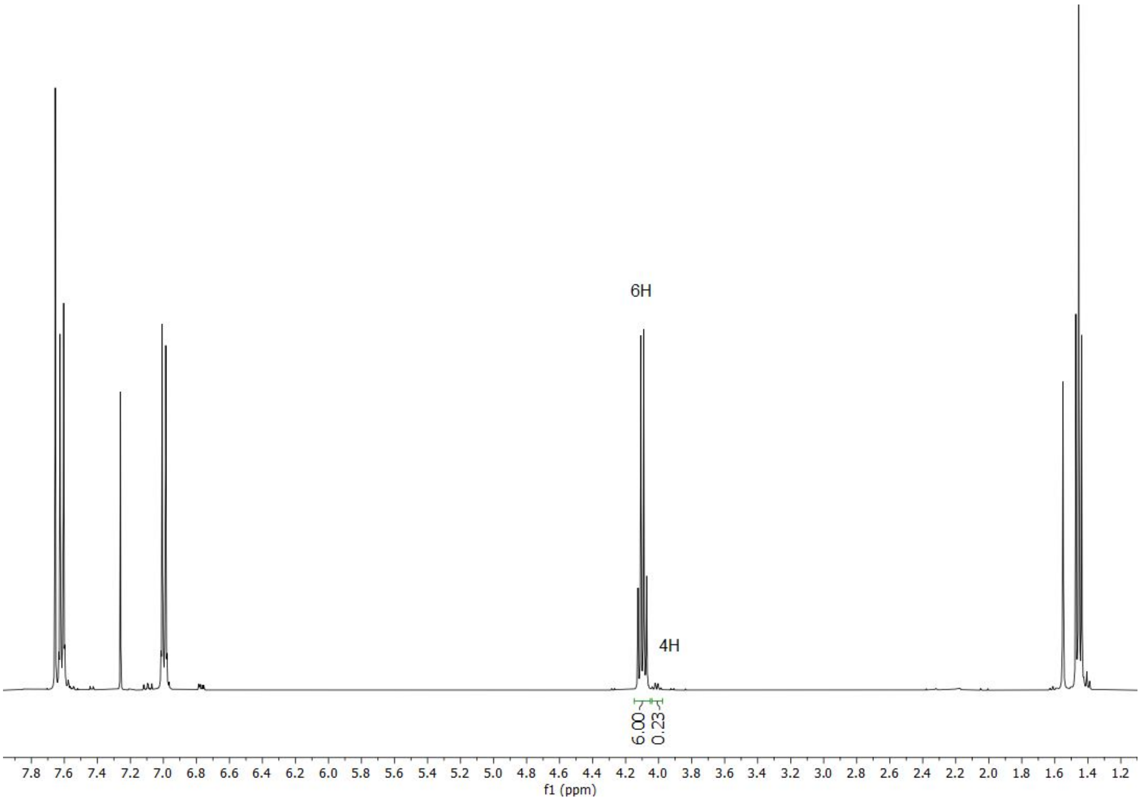


**Figure S24.** $^{1}$H-NMR spectrum ($CDCl_3$, 300MHz, 298K) for determination of ratio of isomers of compound (**8**). Resonance with integral value of 6 (6 H) corresponds to isomer 1, 3, 5. Resonance with integral value of 0.23 (4 H) corresponds to isomer 1, 2, 4.

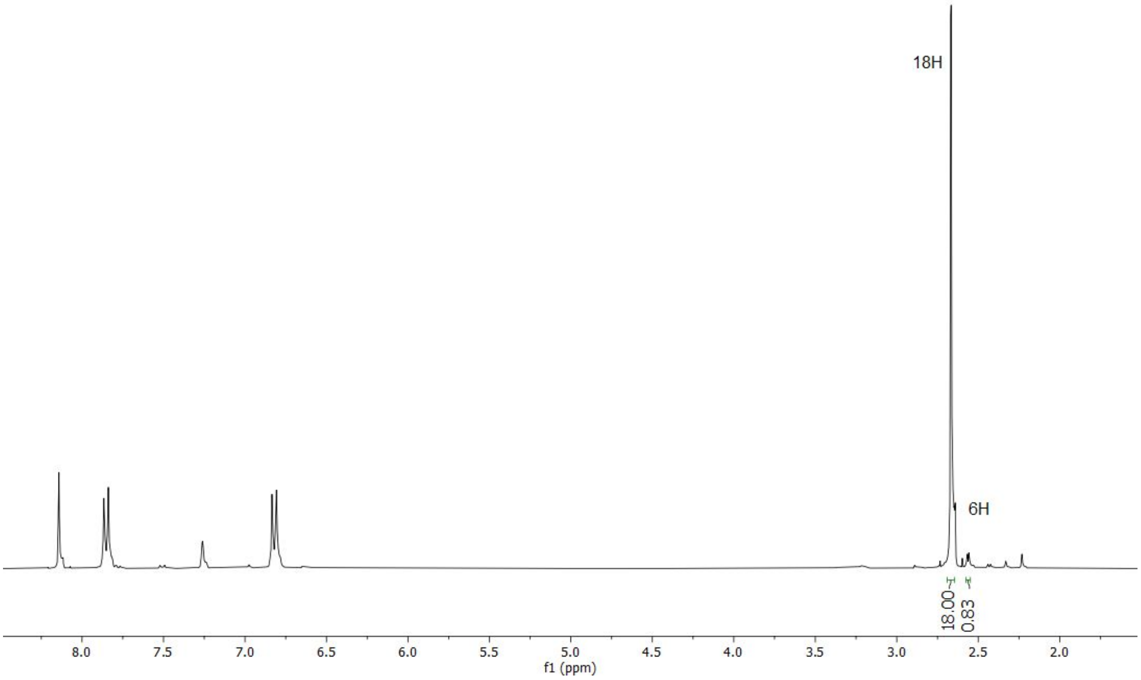


**Figure S25.** $^{1}$H-NMR spectrum ($CDCl_3$, 300MHz, 298K) for determination of ratio of isomers of compound (**9**). Resonance with integral value of 18 (18 H) corresponds to isomer 1, 3, 5. Resonance with integral value of 0.83 (6 H) corresponds to isomer 1, 2, 4.

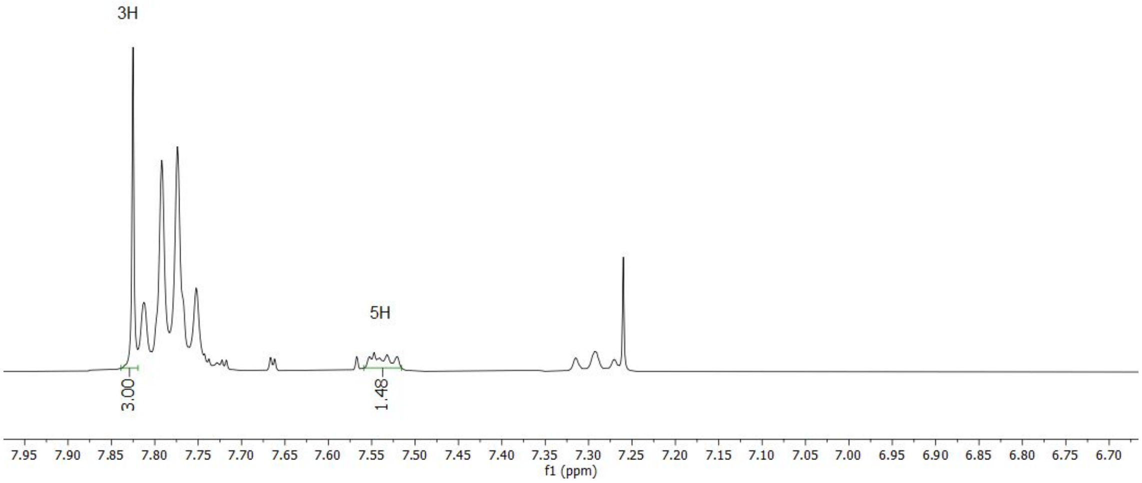


**Figure S26.** $^1$H-NMR spectrum ($CDCl_3$, 300MHz, 298K) for determination of ratio of isomers of compound (**12**). Resonance with integral value of 3 (3 H) corresponds to isomer 1, 3, 5. Resonance with integral value of 1.48 (5 H) corresponds to isomer 1, 2, 4.

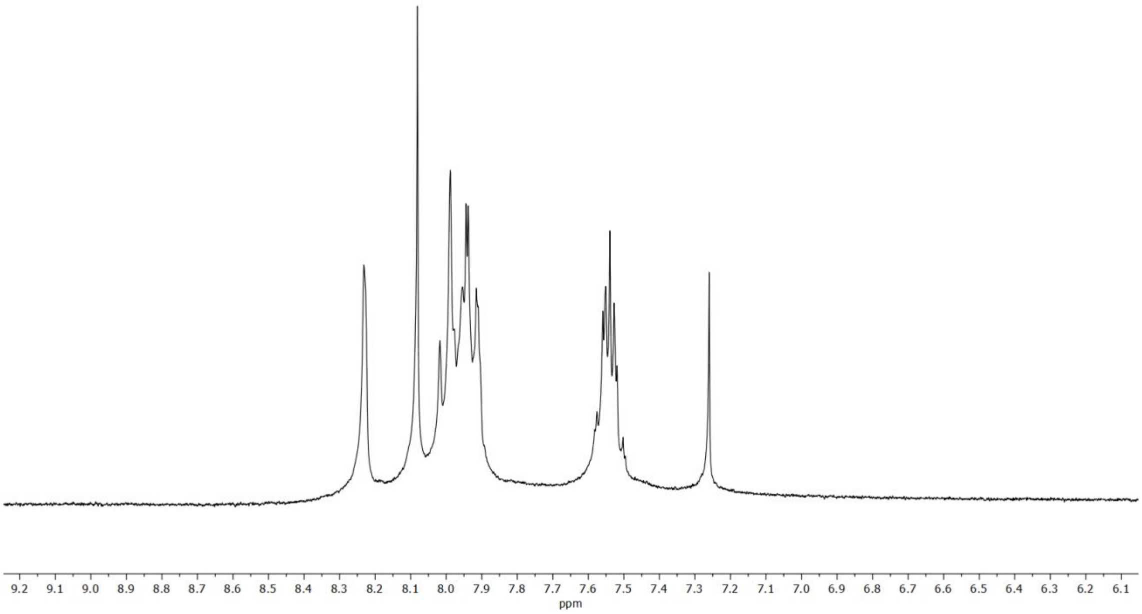


**Figure S27.** $^1$H-NMR spectrum ($CDCl_3$, 300MHz, 298K) for determination of ratio of isomers of compound (**13**). Isomer 1,2,4 is not observed.

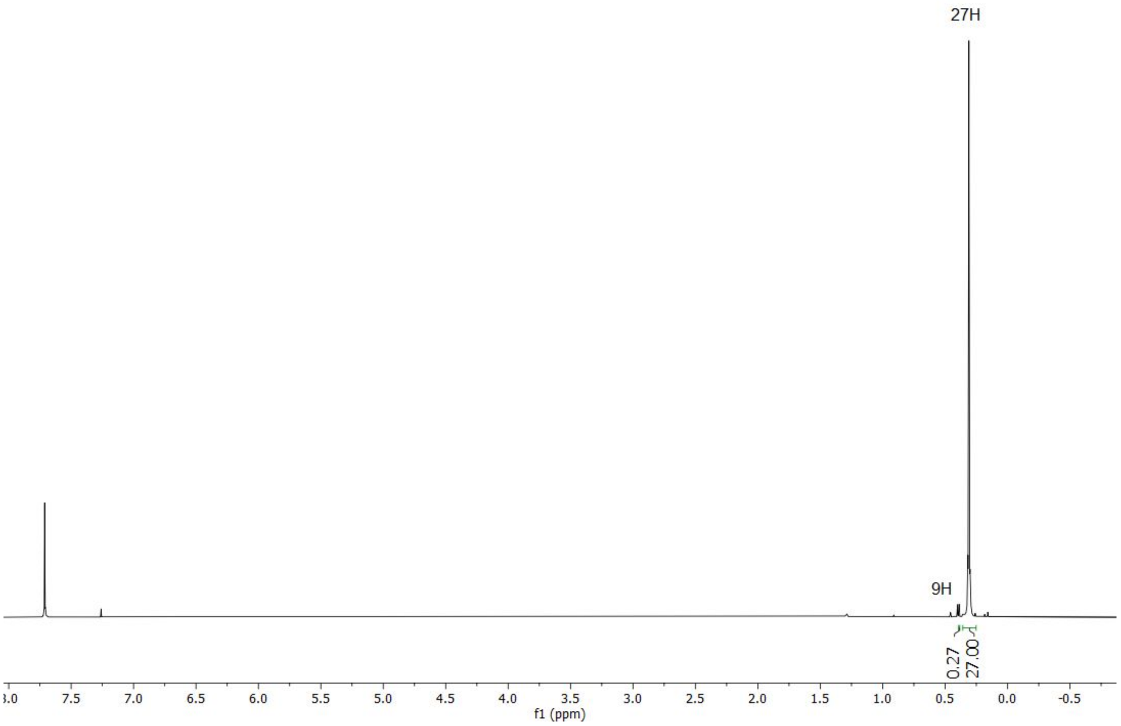


**Figure S28.** $^1$H-NMR spectrum ($CDCl_3$, 300MHz, 298K) for determination of ratio of isomers of compound (**14**). Resonance with integral value of 27 (27 H) corresponds to isomer 1, 3, 5. Resonance with integral value of 0.27 (9 H) corresponds to isomer 1, 2, 4.

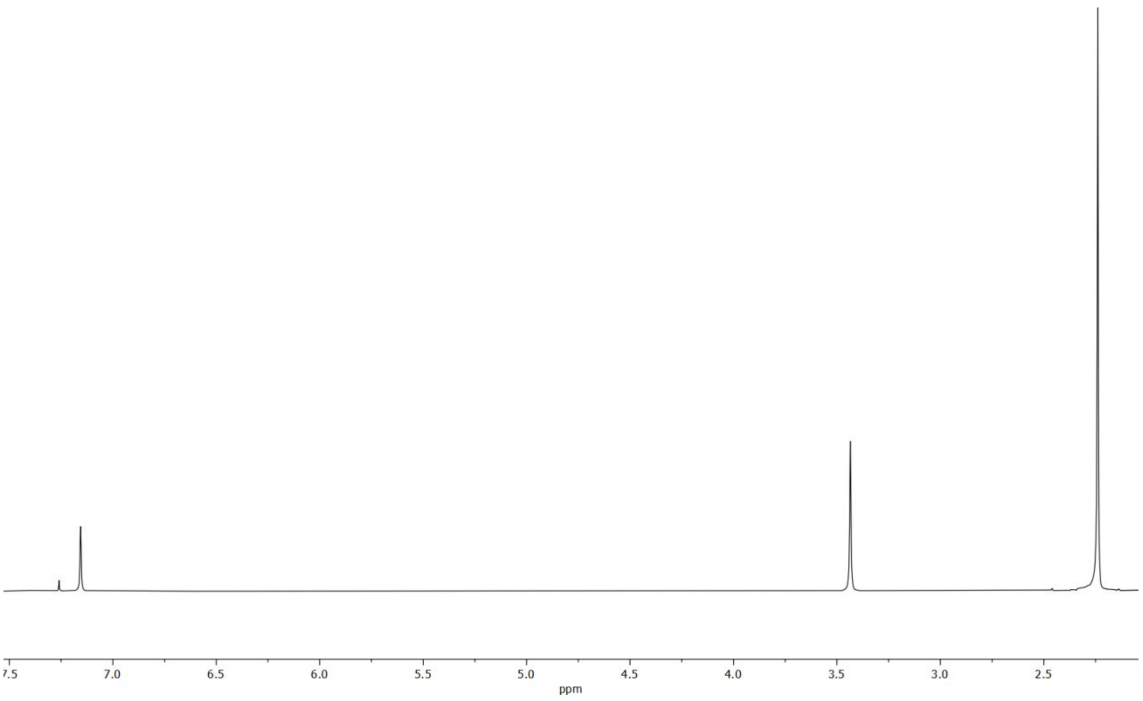


**Figure S29.** $^1$H-NMR spectrum ($CDCl_3$, 300MHz, 298K) for determination of ratio of isomers of compound (**15**). Isomer 1,2,4 is not observed.

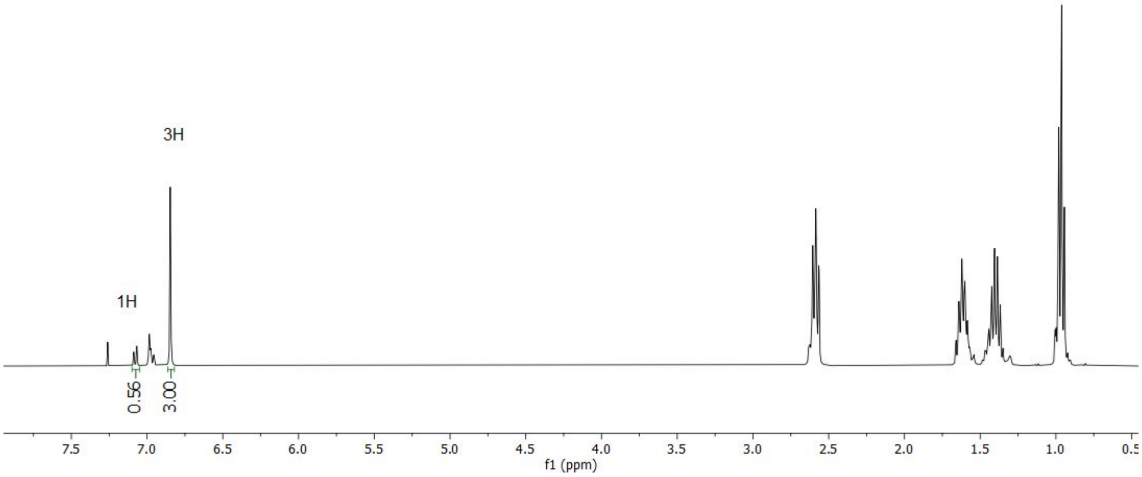


**Figure S30.** $^{1}$H-NMR spectrum ($CDCl_3$, 300MHz, 298K) for determination of ratio of isomers of compound (**16**). Resonance with integral value of 3 (3 H) corresponds to isomer 1, 3, 5. Resonance with integral value of 0.56 (1 H) corresponds to isomer 1, 2, 4.

## 10. Spectroscopical details of isolated products

**Triphenylbenzene (5)**. After flash column chromatography (ethyl acetate/ hexane, 1/9), the expected product was isolated as a white solid in a 90% yield (0.275 g, 0.9 mmol) of both isomers (93:7). MS (EI, 70 ev): m/z (intensity) = 306 (100) $[M]^+$.

**1,3,5-isomer** [3]**:** $^{1}$H-NMR ($CDCl_3$, 300MHz, 298K): δ 7.81 (s, 3 H), 7.75-7.70 (m, 6 H), 7.53-7.47 (m, 6 H), 7.44-7.38 (m, 3 H).$^{13}$C-{$^{1}$H}-NMR ($CDCl_3$, 75MHz, 298K): δ 142.4, 141.2, 129.9, 127.6, 127.4, 125.2.

**1,2,4-isomer** [4]**:** $^{1}$H-NMR ($CDCl_3$, 300MHz, 298K): δ 7.69-7.62 (m, 4 H), 7.53-7.36 (m, 4H), 7.24-7.15 (m, 10 H). $^{13}$C-{$^{1}$H}-NMR ($CDCl_3$, 75MHz, 298K): δ: 141.6, 141.2, 141.1, 140.7, 140.5, 139.7, 131.2, 130.5, 130.0, 129.6, 129.0, 128.1, 128.0, 127.6, 127.3, 126.8, 126.7, 126.3.

**Tris(2-methylphenyl)benzene (6)**: After flash column chromatography (ethyl acetate/ hexane, 1/9), the expected product was isolated as a white solid in an 82% yield (0.285 g, 0.82 mmol) of both isomers (88:12). MS (EI, 70 ev): m/z (intensity) = 348 (100) $[M]^+$, 333 (17) $[M-Me]^+$.

**1,3,5-isomer** [3]**:** $^1$H-NMR ($CDCl_3$, 300MHz, 298K) δ 7.46-7.31 (m, 15H), 2.39 (s, 9H). $^{13}$C-{$^1$H}-NMR ($CDCl_3$, 75MHz, 298K): 142.0, 141.8, 135.7, 130.7, 130.3, 128.9, 127,7, 126,2, 21.0.

**1,2,4-isomer** [5]**:** $^1$H-NMR ($CDCl_3$, 300MHz, 298K) δ 7.35-7.09 (m, 15H), 2.33 (s, 3H), 2.20 (s, 6H). $^{13}$C-{$^1$H}-NMR ($CDCl_3$, 75MHz, 298K): δ 141.5, 140.3, 139.2, 135.7, 135.4, 131.4, 130.4, 129.9, 129.8, 127.6, 127.3, 126.8, 125.8, 124.8, 20,6.

**Tris(4-methoxy-2-methylphenyl)benzene (7)**. After flash column chromatography (ethyl acetate/ hexane, 1/9), the expected product was isolated as a pale-white solid in a 85% yield (0.372 g, 0.85 mmol) of both isomers (88:12). MS (EI, 70 ev): m/z (intensity) = 438 (100) $[M]^+$, 423 (5) $[M-Me]^+$.

**1,3,5-isomer** [5]**:** $^1$H-NMR ($CDCl_3$, 300MHz, 298K): δ 7.28 (s, 3H), 7.22 (s, 3H), 6.84 (m, 6H], 3.86 (s, 9H), 2.38 (s, 9H). $^{13}$C-{$^1$H}-NMR ($CDCl_3$, 75MHz, 298K): δ 158.9, 141.1, 136.9, 134.6, 131.1, 129.5, 128.8, 115.9, 111.3, 55.4, 21.1.

**1,2,4-isomer** [5]**:** $^1$H-NMR ($CDCl_3$, 300MHz, 298K) δ δ 7.70 - 7.48 (m, 3H), 7.14 (m, 2H), 6.98 (d, 1H), 6.90 - 6.75 (m, 4H), 6.66 (m, 2H), 3.96 (s, 3H), 3.92 (s, 3H), 3.88 (s, 6H), 3.63 (s, 6H). $^{13}$C-{$^1$H}-NMR ($CDCl_3$, 75MHz, 298K):158.7, 158.1, 139.8, 137.0, 136.8, 134.2, 132.0, 131.0, 130.7, 129.1, 128.3, 127.6, 115.8, 115.0, 111.2, 110.5, 55.3, 55.1, 21.0.

**Tris(4-ethoxyphenyl)benzene (8)**. After flash column chromatography (ethyl acetate/ hexane, 1/9), the expected product was isolated as a white solid in a 90% yield (0.394 g, 0.9 mmol) of both isomers (95:5). MS (EI, 70 ev): m/z (intensity) = 438 (100) $[M]^+$.

**1,3,5-isomer** [6]**:** $^1$H-NMR ($CDCl_3$, 300MHz, 298 K): δ 7.65 (s, 3H), 7.64-7.58 (m, 6H), 7.02-6.97 (m, 6H), 4.10 [c, 6H, $J_{H-H}$=5.4 Hz], 1.45 [t, 9H, $J_{H-H}$=5,4 Hz].$^{13}$C-{$^1$H}-NMR ($CDCl_3$, 75MHz, 298 K): δ 158.8, 142.0, 133.9, 128.5, 123.9, 114.9, 63.7, 15.0.

**1,2,4-isomer** is not reported in the literature. Due to the low ratio observed in our samples, a detailed assignment of the $^1$H and $^{13}$C resonances is not possible.

**Tris(4-(*N*,*N*-dimethylamino)phenyl)benzene (9)**. After flash column chromatography (ethyl acetate/ hexane, 1/9), the expected product was isolated as a pale-yellow solid in a 90% yield (0.392 g, 0.9 mmol) of both isomers (88:12). MS (EI, 70 ev): m/z (intensity) = 435 (100) $[M]^+$, 420 (10) $[M-Me]^+$.

**1,3,5-isomer** [7]**:** $^{1}$H-NMR ($CDCl_3$, 300MHz, 298 K): δ 7.68 (s, 3H), 7.62 [d, 6H, $J_{H-H}$=9 Hz], 6.88 [d, 6H, $J_{H-H}$=9 Hz], 3.04 (s, 18H).$^{13}$C-{$^{1}$H}-NMR ($CDCl_3$, 75MHz 298K): δ 149.9, 142.1, 130.7, 128.1, 122.8, 113.1, 40.9.

**1,2,4-isomer** [5]**:** $^{1}$H-NMR ($CDCl_3$, 300MHz, 298K) δ δ 7.60 – 7.46 (m, 4H), 7.43 (d, 1H), 7.12 (m, 4H), 6.82 (d, 2H), 6.67 (m, 4H), 3.01 (s, 6H), 2.94 (s, 12H). $^{13}$C-{$^{1}$H}-NMR ($CDCl_3$, 75MHz, 298K): 148.6, 148.3, 143.0, 141.6, 139.5, 130.3, 129.1, 128.6, 128.5, 128.4, 128.0, 126.3, 125.9, 114.4, 114.0, 113.5, 113.1, 42.0, 41.7.

**Tris(trifluoromethylphenyl)benzene (12)**. After flash column chromatography (ethyl acetate/ hexane, 1/9), the expected product was isolated as a white solid in a 78% yield (0.398 g, 0.78 mmol) of both isomers (77:23). MS (EI, 70 ev): m/z (intensity) = 510 (100) $[M]^+$, 441 (27) $[M-CF_3]^+$.

**1,3,5-isomer** [3]**:** $^{1}$H-NMR ($CDCl_3$, 300MHz, 298K): δ 7.82 (s, 3 H), 7.78 (q, 12 H). $^{13}$C-{$^{1}$H}-NMR ($CDCl_3$, 75MHz, 298K): δ. 144.3, 141.9, 130.4 (q, $J^{1}_{C-F}$= 36Hz), 128.0, 126.5, 126.3 (q, $J^{3}_{C-F}$= 3Hz).$^{19}$F{$^{1}$H}-RMN ($CDCl_3$, 376MHz, 298K): δ - 61.4.

**1,2,4-isomer** [5]**:** $^{1}$H-NMR ($CDCl_3$, 300MHz, 298K) δ 7.80–7.70 (m, 5H), 7.67 (m, 1H), 7.60–7.45 (m, 5H), 7.29 (m, 4H). $^{13}$C-{$^{1}$H}-NMR ($CDCl_3$, 75MHz, 298K): 144.1, 143.5, 143.2, 139.9, 139.0, 131.5, 130.1, 129.6, 129.0, 127.5, 127.2, 126.0, 125.9, 125.2. $^{19}$F{$^{1}$H}-RMN ($CDCl_3$, 376MHz, 298K): δ - 62.9.

**Trisnaphthylbenzene (13)**: After flash column chromatography (ethyl acetate/ hexane, 5/95), the expected product was isolated as a white solid in an 80% yield (0.363 g, 0.80 mmol). Ratio of isomers >99:1. MS (EI, 70 ev): m/z (intensity) = 456 (100) $[M]^+$.

**1,3,5-isomer** [8]**:** $^{1}$H-NMR ($CDCl_3$, 300MHz, 298K): δ 8.23 (s, 3H), 8.08 (s, 3H), 8.05-7.80 (m, 12H), 7.65-7.40 (m, 6H).$^{13}$C{$^{1}$H}-NMR ($CDCl_3$, 75MHz, 298K): δ 143.2, 139.2, 134.5, 133.6, 129.4, 129.1, 129.0, 128.5, 127.2, 126.9, 126.5.

**Tris(trimethylsilyl)benzene (14)**. After flash column chromatography (ethyl acetate/ hexane, 1/9), the expected product was isolated as a brown oil in a 98% yield (0.289 g, 0.98 mmol) of both isomers (99:1). MS (EI, 70 ev): m/z (intensity) = 294 (40) $[M]^+$, 279 (84) $[M-Me]^+$.

**1,3,5-isomer** [7]**:** $^{1}$H-NMR ($CDCl_3$, 300MHz, 298K): δ 7.71 (s, 3H), 0.31 (s, 27H).$^{13}$C-{$^{1}$H}-NMR ($CDCl_3$, 75MHz, 298K): δ 139.0, 138.4, -0.9.

**1,2,4-isomer [9]:** $^1$H-NMR ($CDCl_3$, 300MHz, 298K) δ 7.85 (s, 1H), 7.67 (d, 1H), 7.49 (d, 1H), 0.37 (s, 18H), 0.27 (s, 9H) $^{13}$C-{$^1$H}-NMR ($CDCl_3$, 75MHz, 298K): 146.8, 144.9, 140.1, 139.5, 139.0, 138.4, 134.6, 132.9, 2.1, 2.0, -0.9, -1.1.

**Tris(dimethylaminomethyl)benzene (15):** After flash column chromatography (ethyl acetate/ methanol, 8/2), the expected product was isolated as a white solid in an 83% yield (0.207 g, 0.83 mmol). Ratio of isomers >99:1. MS (EI, 70 ev): m/z (intensity) = 206 (100) [M-$NMe_2$]$^+$, 162 (30) [M-2$NMe_2$]$^+$.

**1,3,5-isomer [10]:** $^1$H-NMR ($CDCl_3$, 300MHz, 298K): δ 7.16 (s, 3H), 3.44 (s, 6H), 2.24 (s, 18H). $^{13}$C-{$^1$H}-NMR ($CDCl_3$, 75MHz, 298K) δ 138.5, 129.2, 64.1, 45.2.

**Tributylbenzene (16)**: After flash column chromatography (ethyl acetate/ hexane, 1/9), the expected product was isolated as a pale-brown oil in an 80% yield (0.197 g, 0.80 mmol) of both isomers (64:36). MS (EI, 70 ev): m/z (intensity) = 246(100) [M]$^+$.

**1,3,5-isomer [7]:** $^1$H-NMR ($CDCl_3$, 300MHz, 298K): δ 6.85 (s, 3H), 2.59 [t, 6H, $J^3_{H-H}$ =6 Hz, C$H_2$], 1.65-1.55 (m, 6H, C$H_2$), 1.46-1.34 (m, 6H, C$H_2$), 0.96 (t, 9H, $J^3_{H-H}$ = 6 Hz, C$H_3$). $^{13}$C-{$^1$H}-NMR ($CDCl_3$, 75MHz, 298K) δ 143.0, 126.2, 36.0, 34.1, 22.9, 14.3.

**1,2,4-isomer [7]:** $^1$H-NMR ($CDCl_3$, 300MHz, 298K) δ 7.04 (d, 1H), 6.95-6.90 (m, 2H), 2.60-2.50 (m, 6H), 1.65-1.50 (m, 6H), 1.45-1.30 (m, 6H), 1.00- 0.90 (m, 9H).$^{13}$C-{$^1$H}-NMR ($CDCl_3$, 75MHz, 298K): 140.4, 140.2, 137.8, 129.4, 129.1, 125.8, 35.5, 33.9, 33.7, 32.6, 32.2, 23.1, 23.0, 22.7, 14.3, 14.2, 14.1.

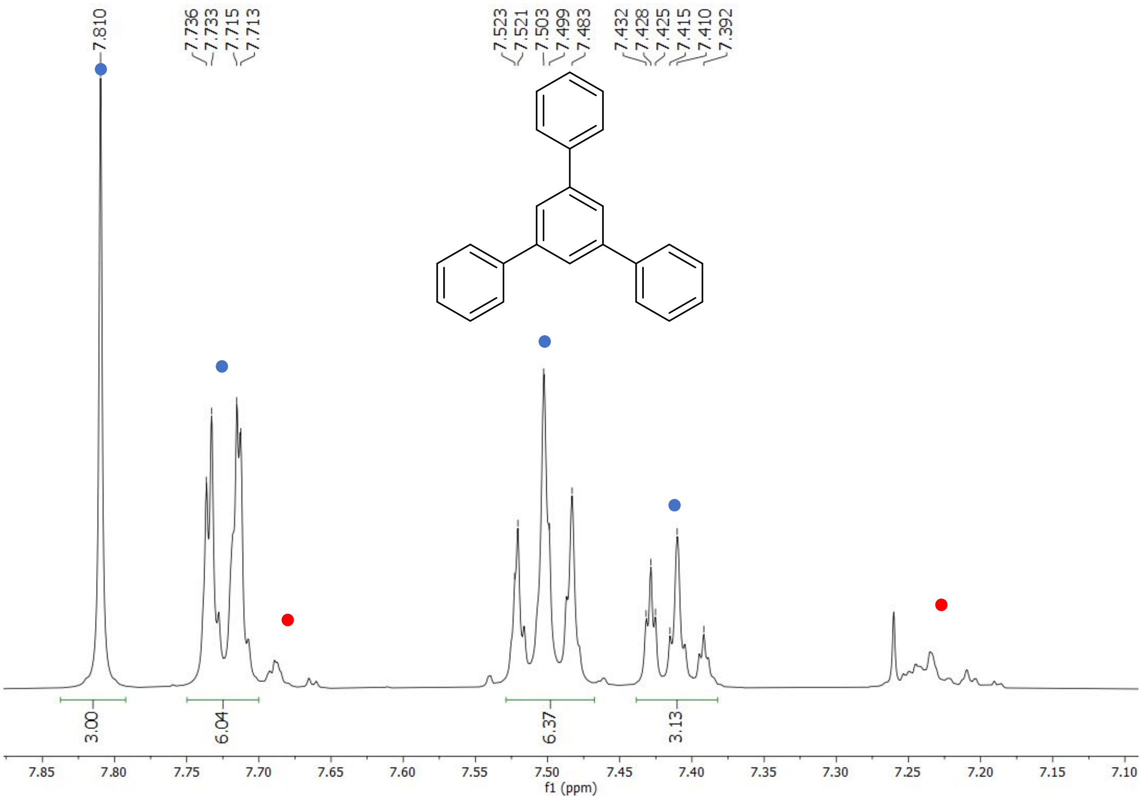


**Figure S31.** $^{1}$H-NMR ($CDCl_3$, 300MHz, 298K) spectrum for **5**. 1,3,5-isomer (●), 1,2,4-isomer (●)

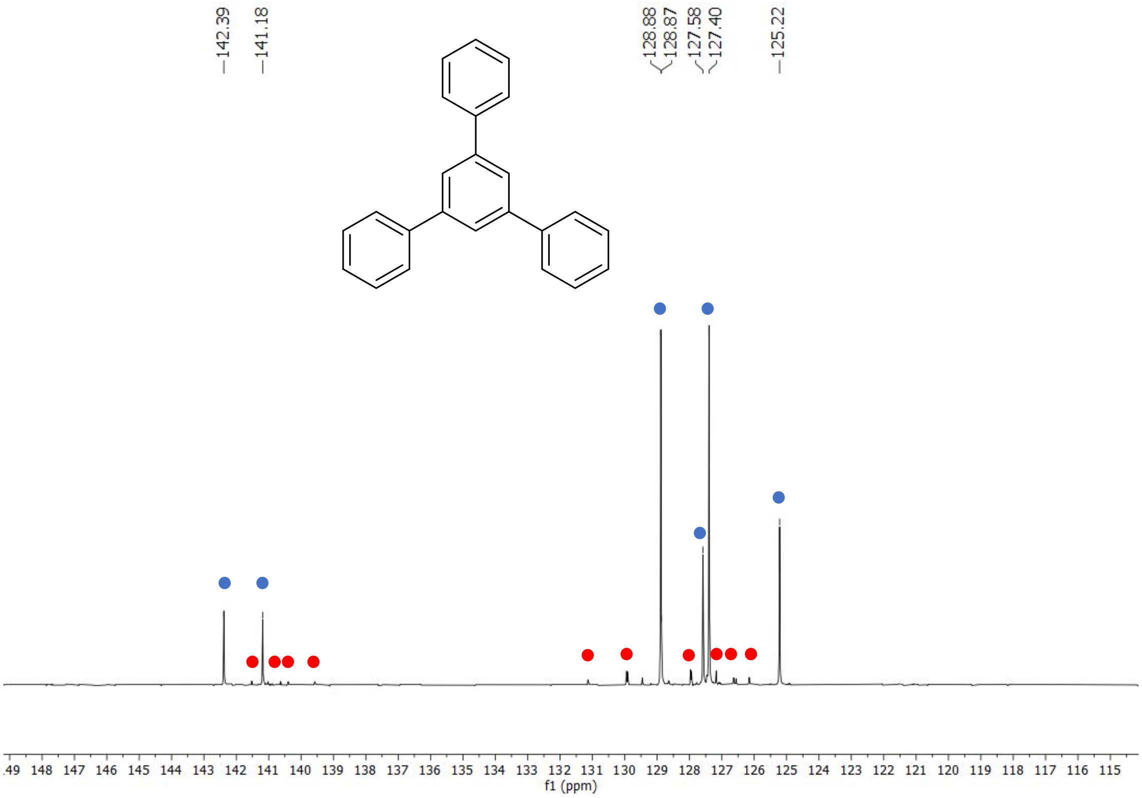


**Figure S32.** $^{13}$C-{$^{1}$H}-NMR ($CDCl_3$, 75 MHz, 298K) spectrum for **5.** 1,3,5-isomer (●), 1,2,4-isomer (●)

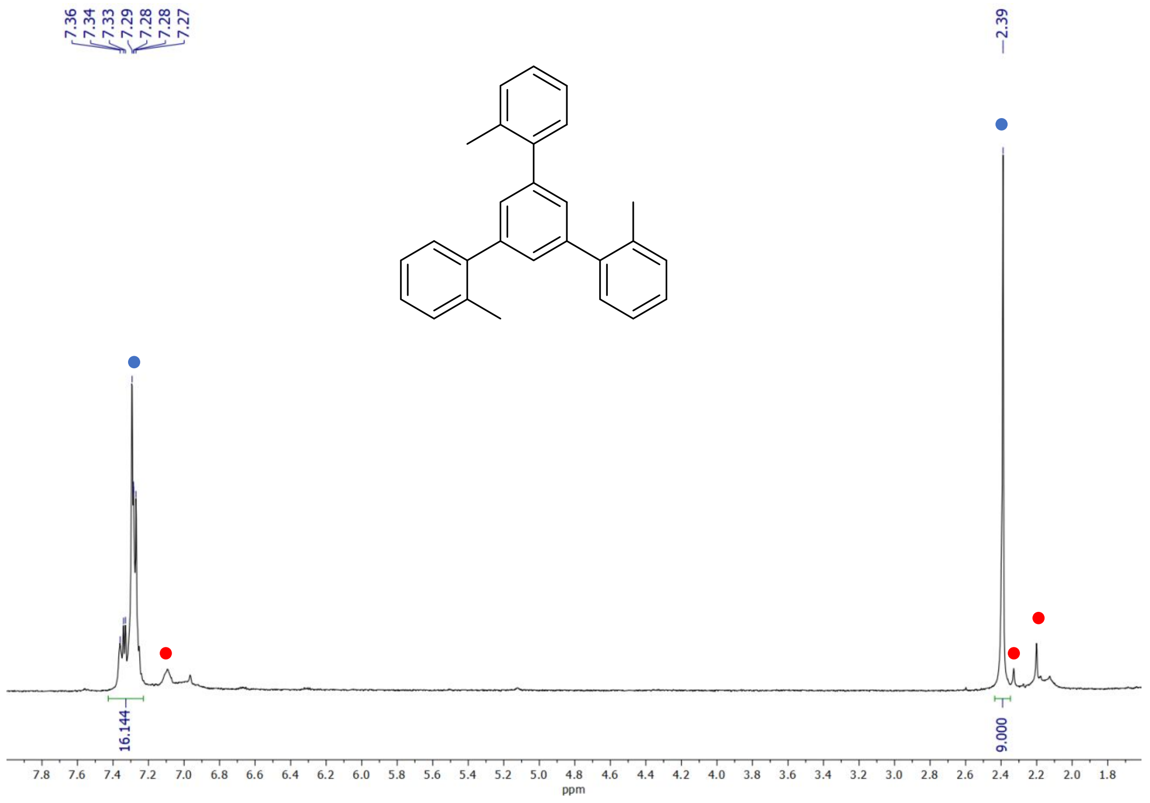


**Figure S33.** $^{1}$H-NMR ($CDCl_3$, 300MHz, 298K) spectrum for **6**. 1,3,5-isomer (●), 1,2,4-isomer (●)

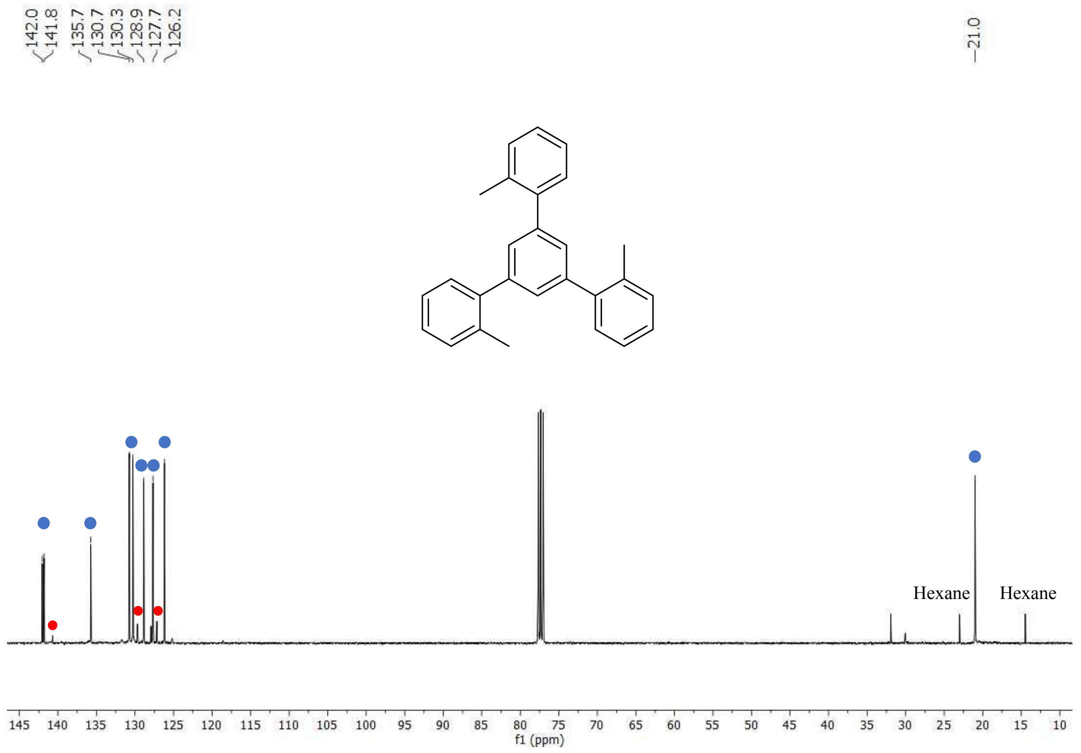


**Figure S34.** $^{13}$C-{$^{1}$H}-NMR ($CDCl_3$, 75 MHz, 298K) spectrum for **6**. 1,3,5-isomer (●), 1,2,4-isomer (●)

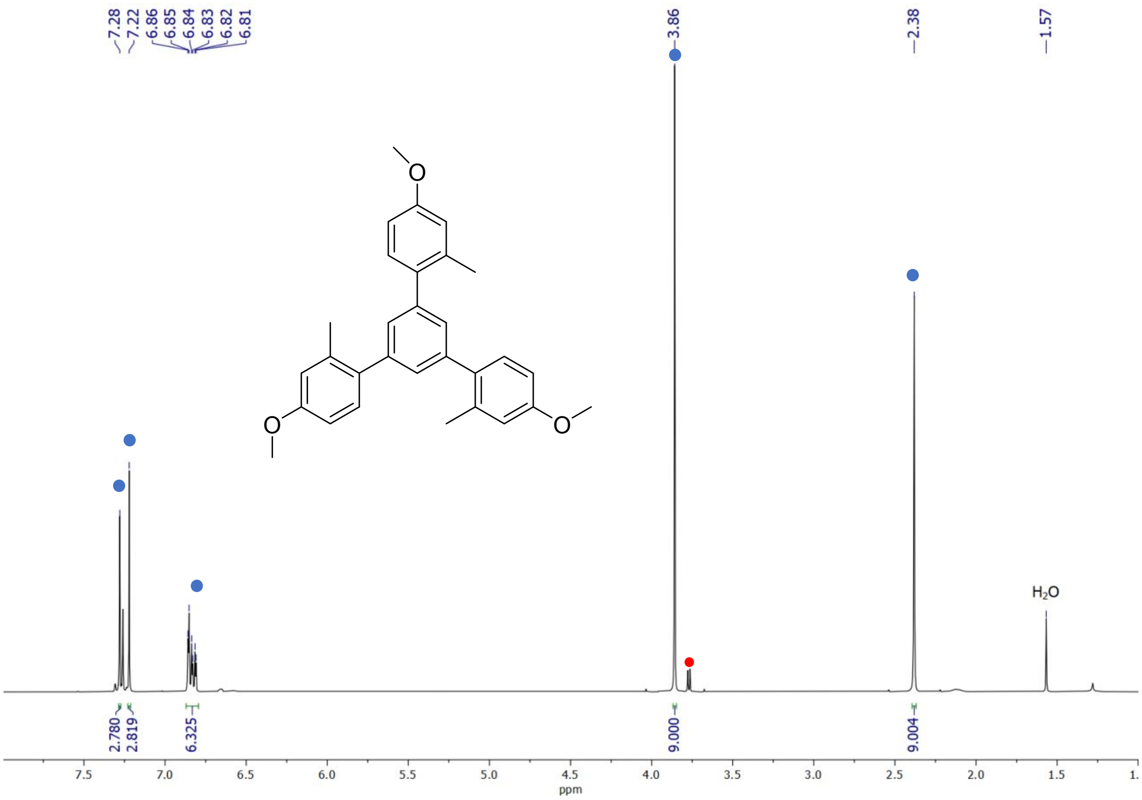


**Figure S35.** $^{1}$H-NMR ($CDCl_3$, 300MHz, 298K) spectrum for **7**. 1,3,5-isomer (●), 1,2,4-isomer (●)

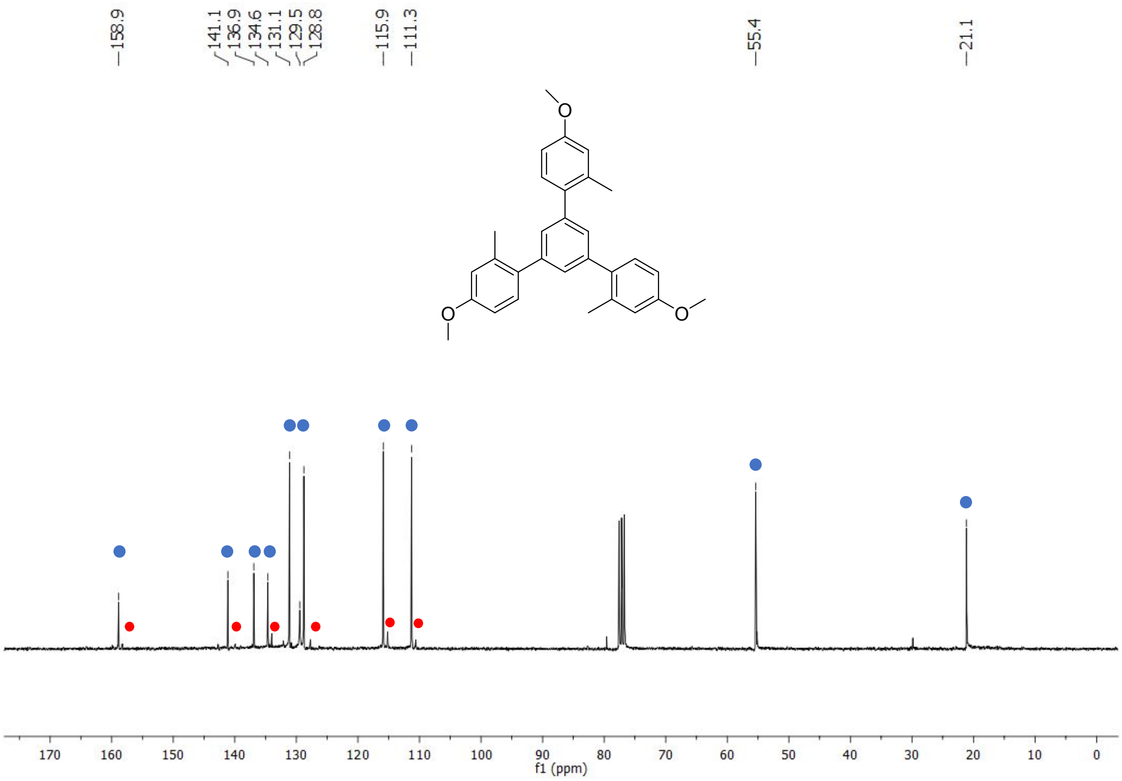


**Figure S36.** $^{13}$C-{$^{1}$H}-NMR ($CDCl_3$, 75 MHz, 298K) spectrum for **7**. 1,3,5-isomer (●), 1,2,4-isomer (●)

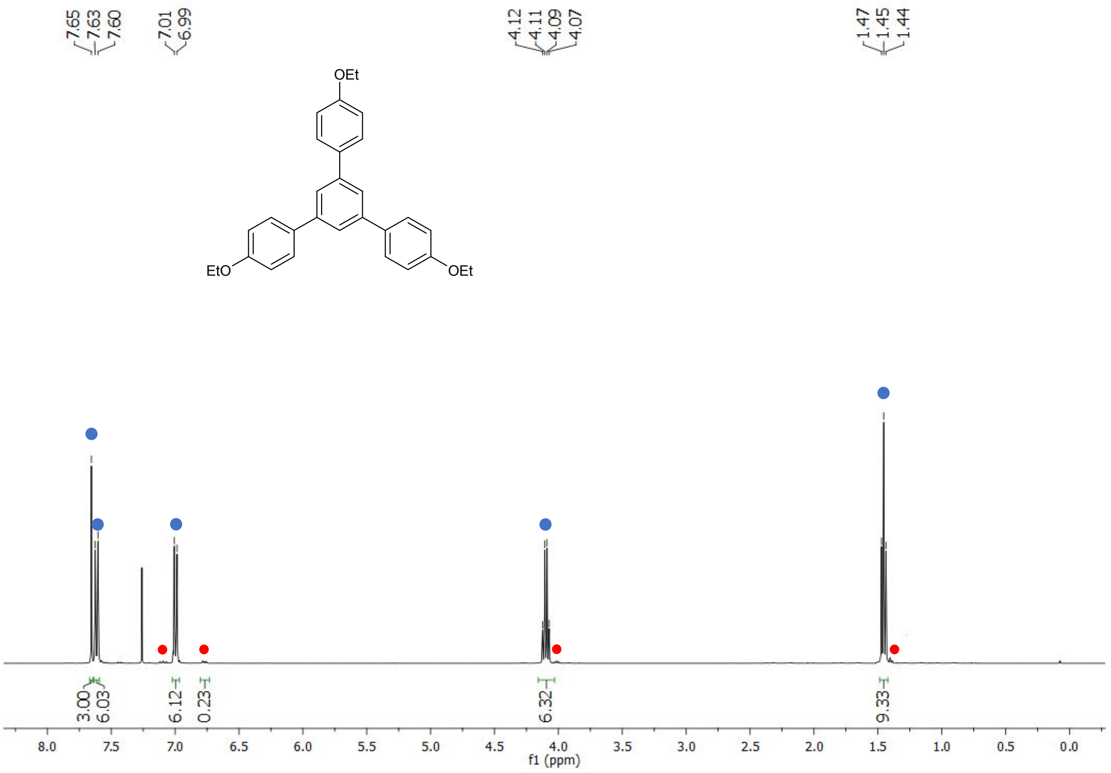


**Figure S37.** $^{1}$H-NMR ($CDCl_3$, 300MHz, 298K) spectrum spectrum for **8** 1,3,5-isomer (●), 1,2,4-isomer (●)

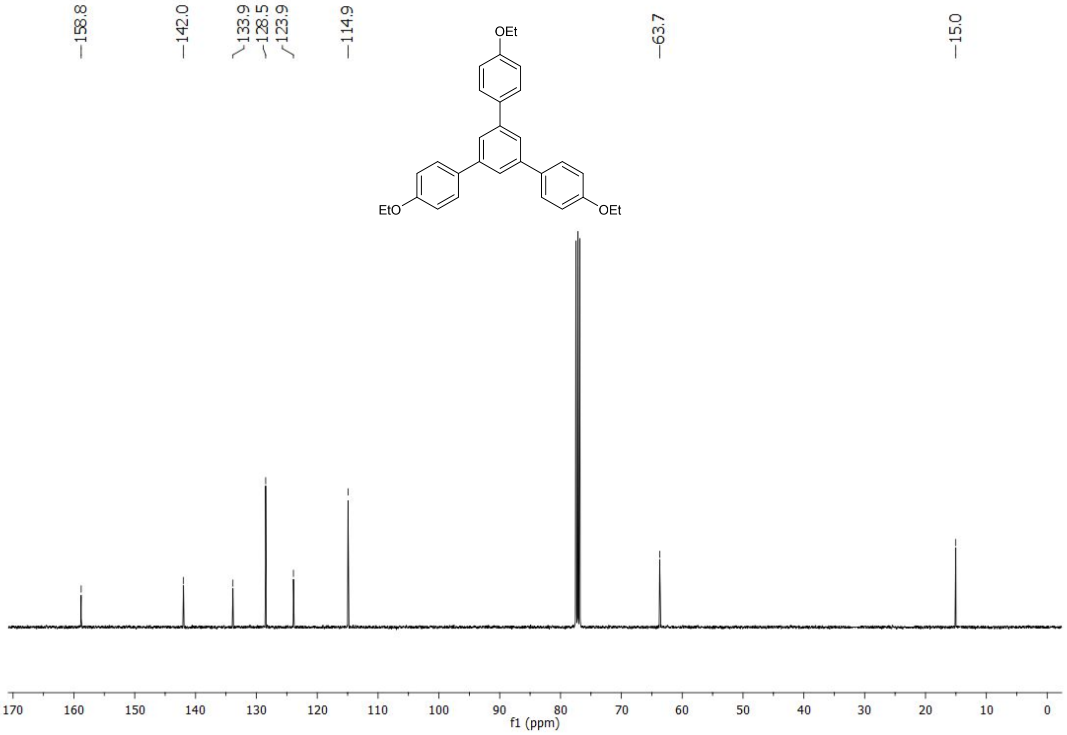


**Figure S38.** $^{13}$C-{$^{1}$H}-NMR ($CDCl_3$, 75 MHz, 298K) spectrum for **8**.

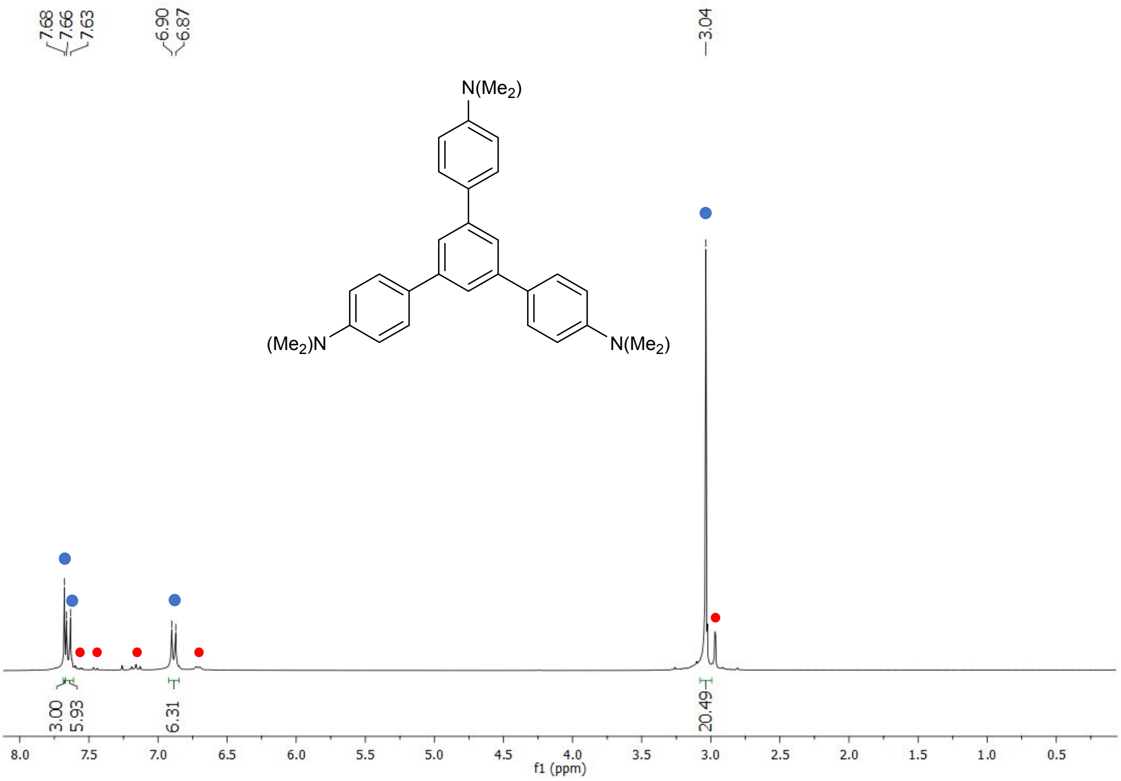


**Figure S39.** $^{1}H$-NMR ($CDCl_3$, 300MHz, 298K) spectrum for **9**. 1,3,5-isomer (●), 1,2,4-isomer (●)

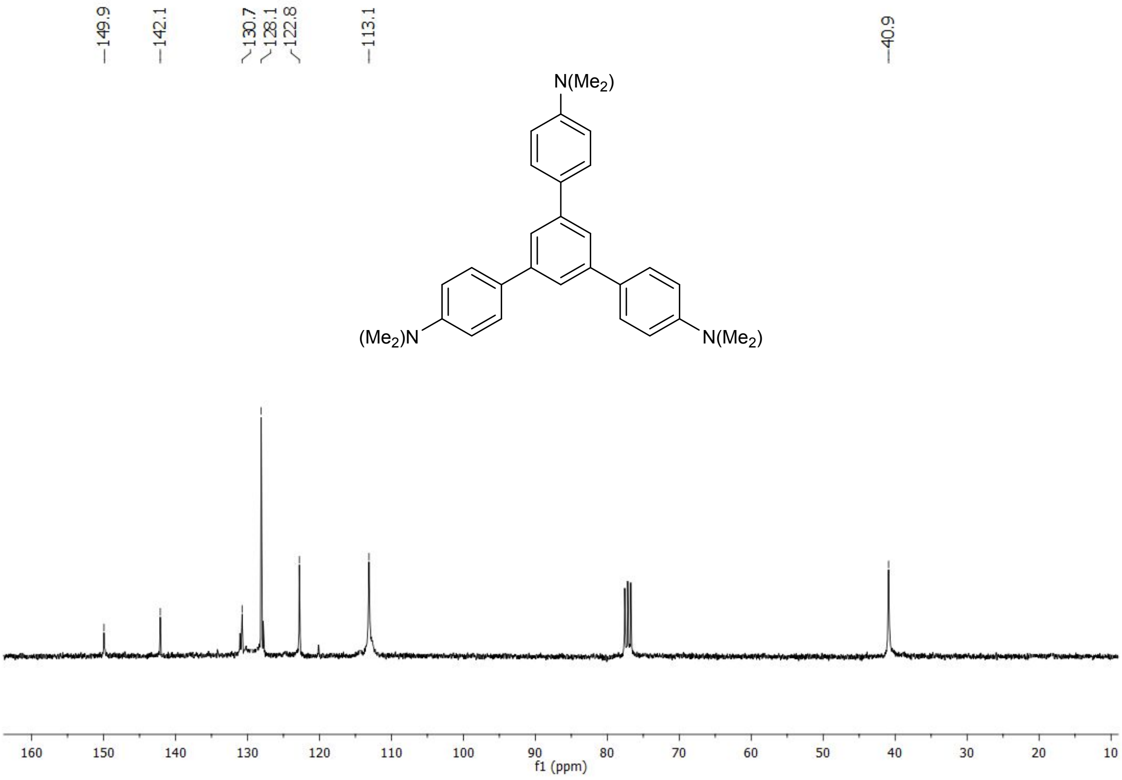


**Figure S40.** $^{13}C$-{$^{1}H$}-NMR ($CDCl_3$, 75 MHz, 298K) spectrum for **9**.

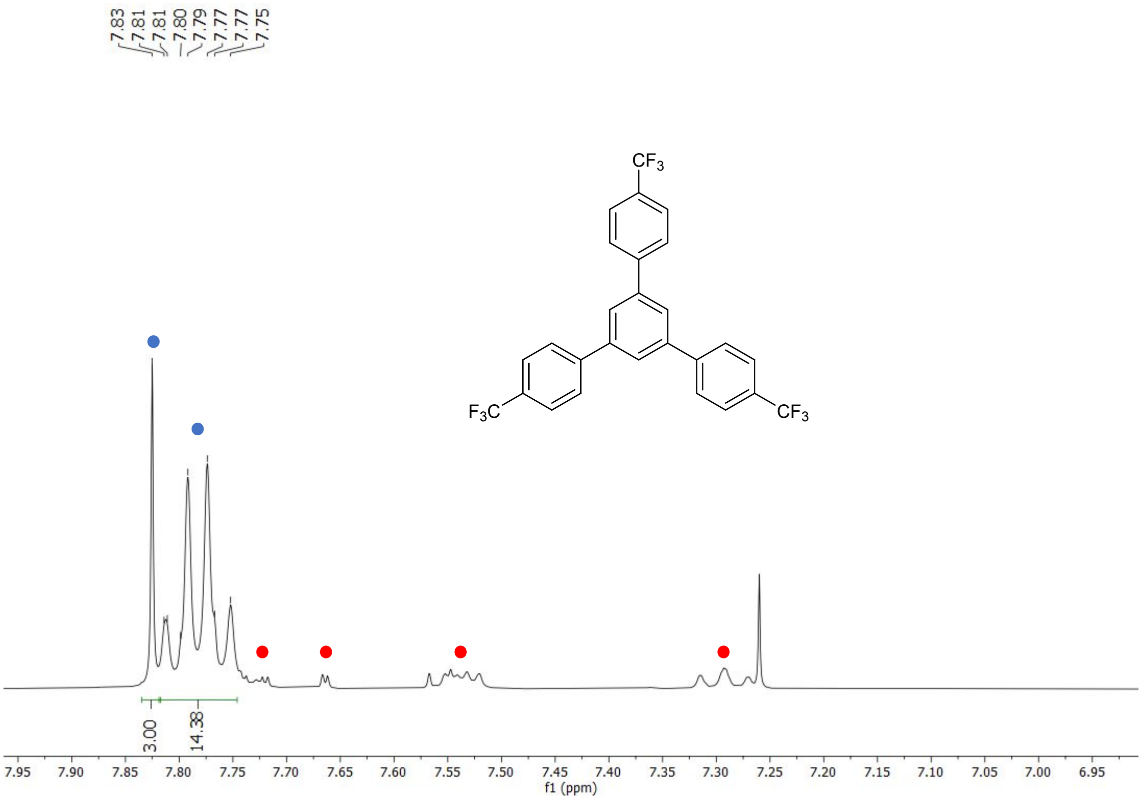


**Figure S41.** $^{1}$H-NMR ($CDCl_3$, 300MHz, 298K) spectrum for **12**. 1,3,5-isomer (●), 1,2,4-isomer (●)

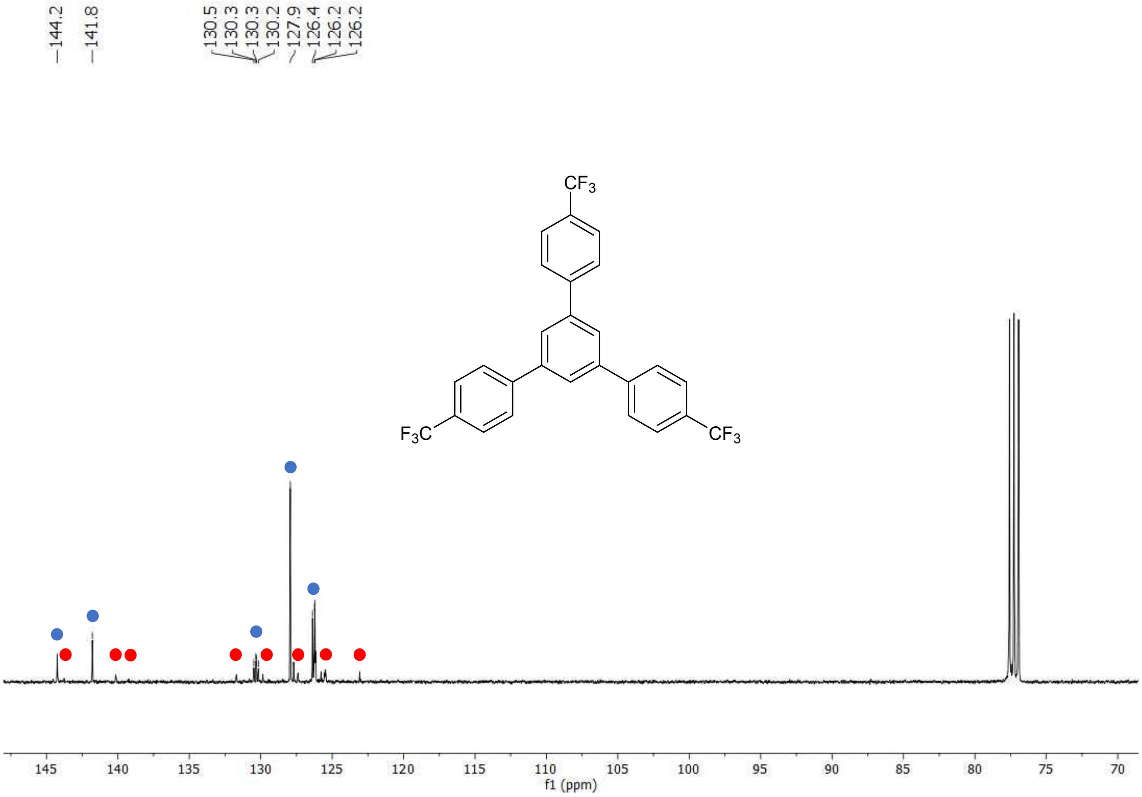


**Figure S42.** $^{13}$C-{$^{1}$H}-NMR ($CDCl_3$, 75 MHz, 298K) spectrum for **12**. 1,3,5-isomer (●), 1,2,4-isomer (●)

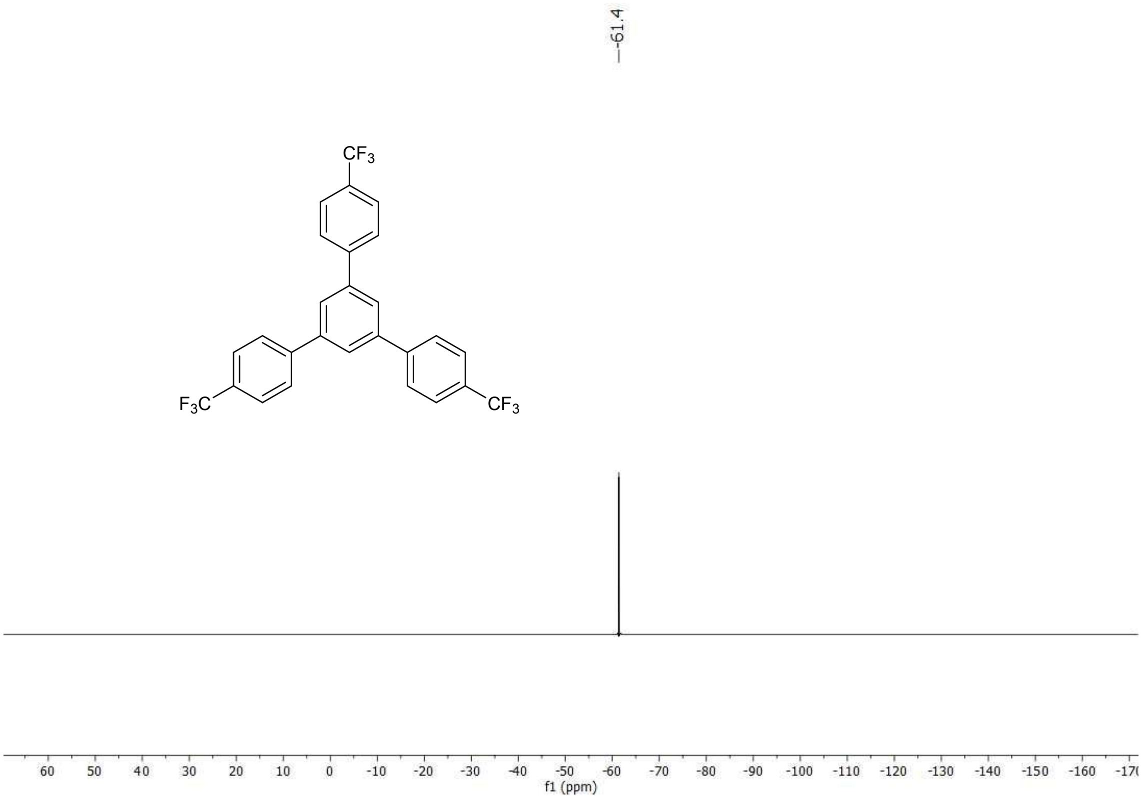


**Figure S43.** $^{19}F$-{$^{1}H$}-NMR ($CDCl_3$, 376 MHz, 298K) **12**.

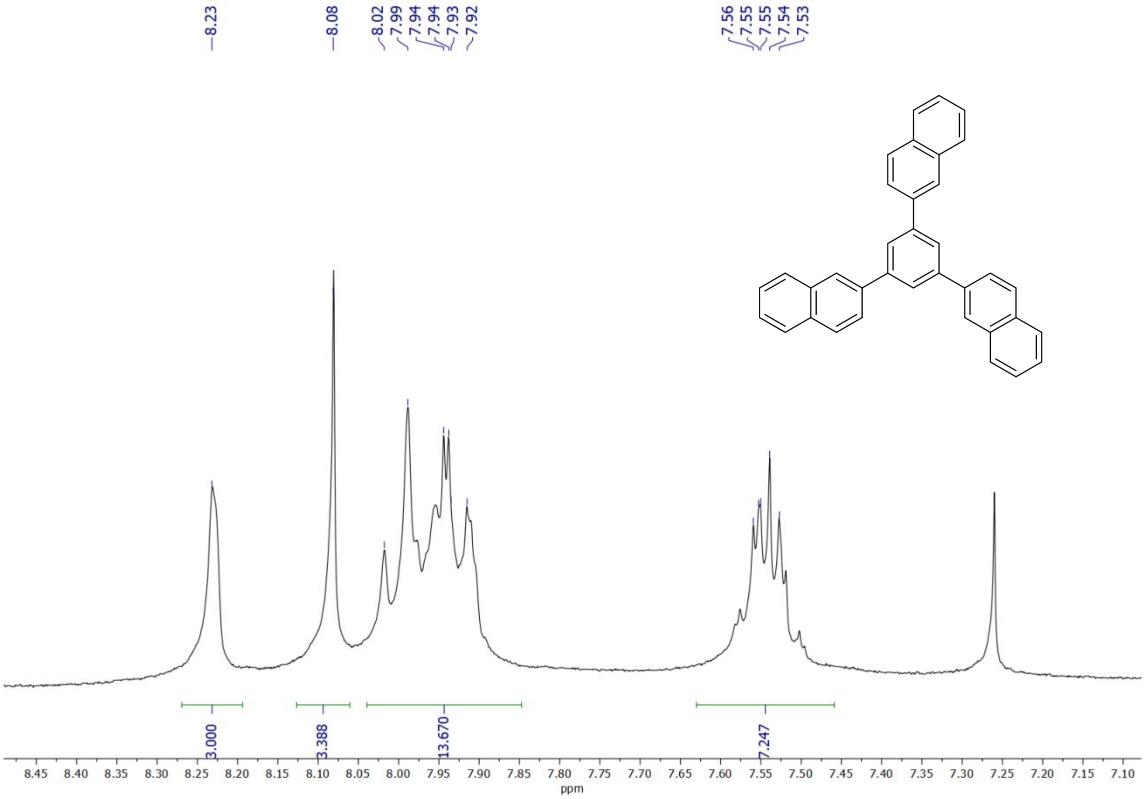


**Figure S44.** $^{1}H$-NMR ($CDCl_3$, 300MHz, 298K) spectrum for **13**.

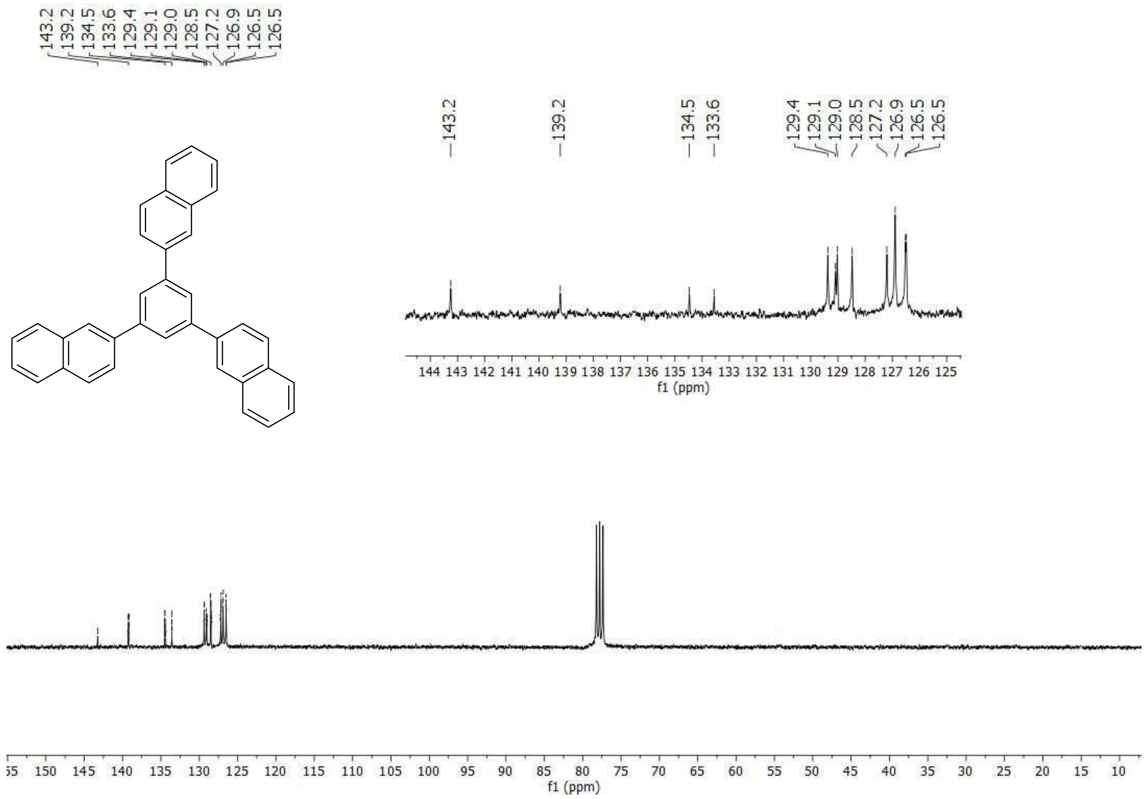


**Figure S45.** $^{13}C$-{$^{1}H$}-NMR ($CDCl_3$, 75 MHz, 298K) spectrum for **13.**

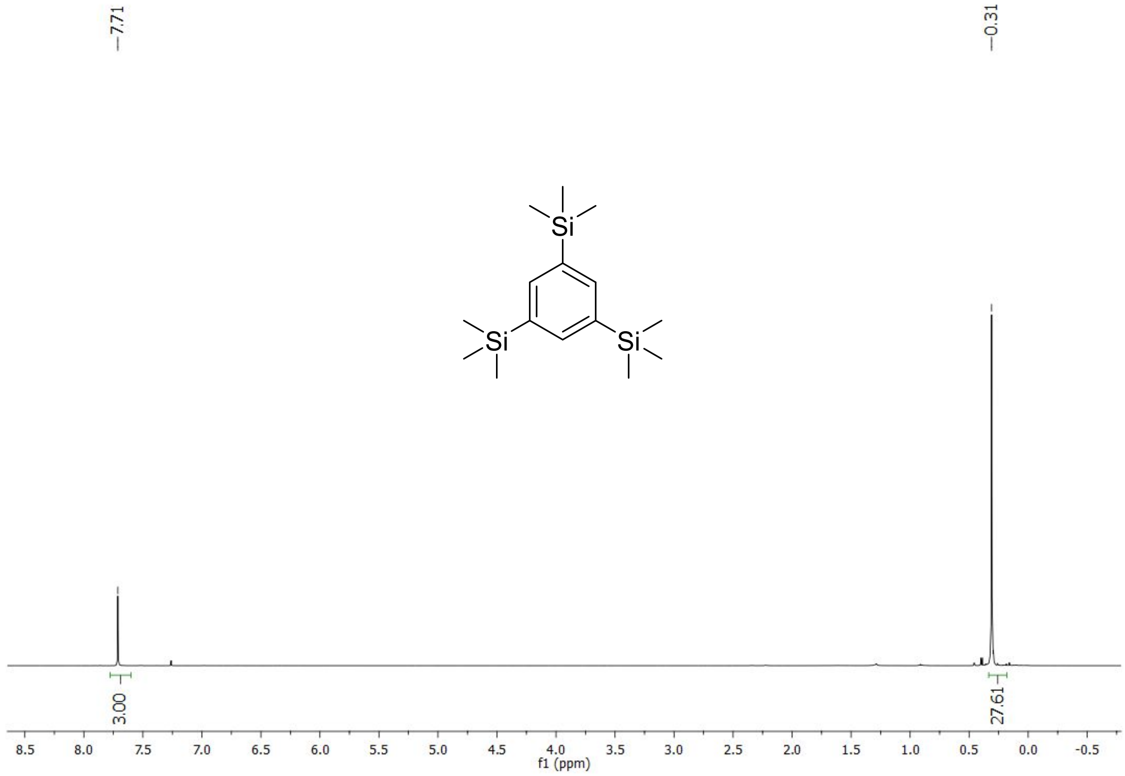


**Figure S46.** $^{1}H$-NMR ($CDCl_3$, 300MHz, 298 K) spectrum for **14**.

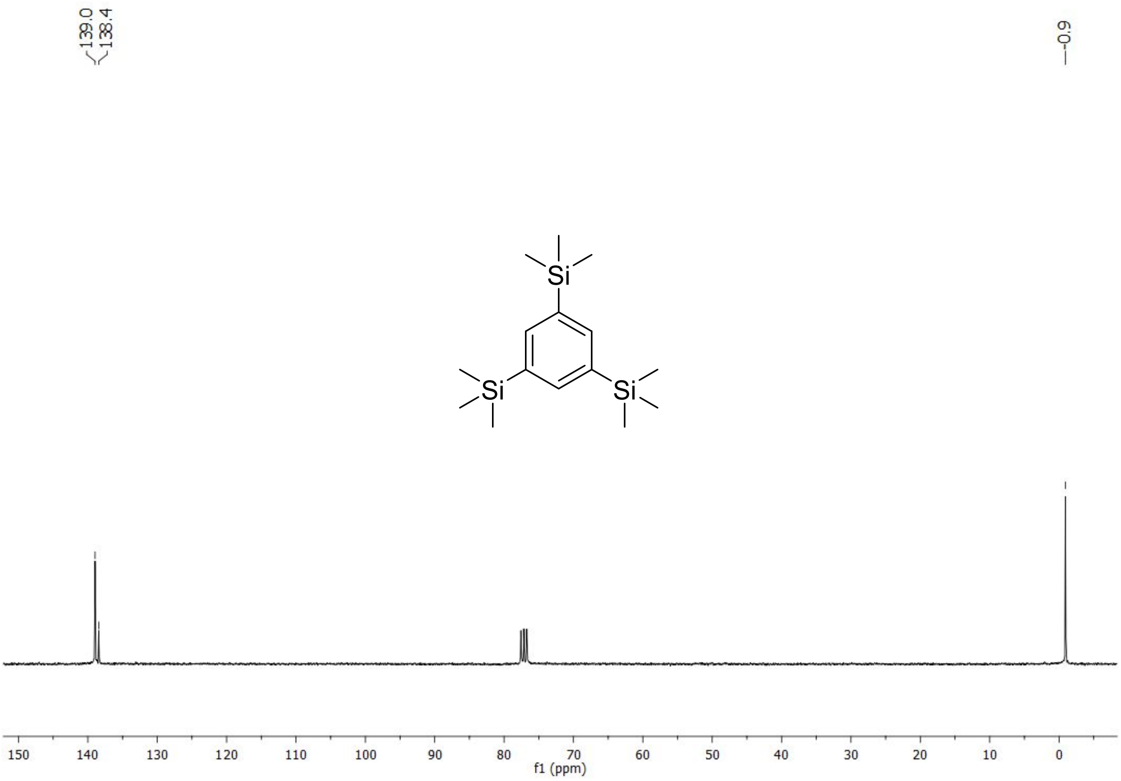


**Figure S47.** $^{13}C-\{^{1}H\}$-NMR ($CDCl_3$, 75 MHz, 298K) spectrum for **14**.

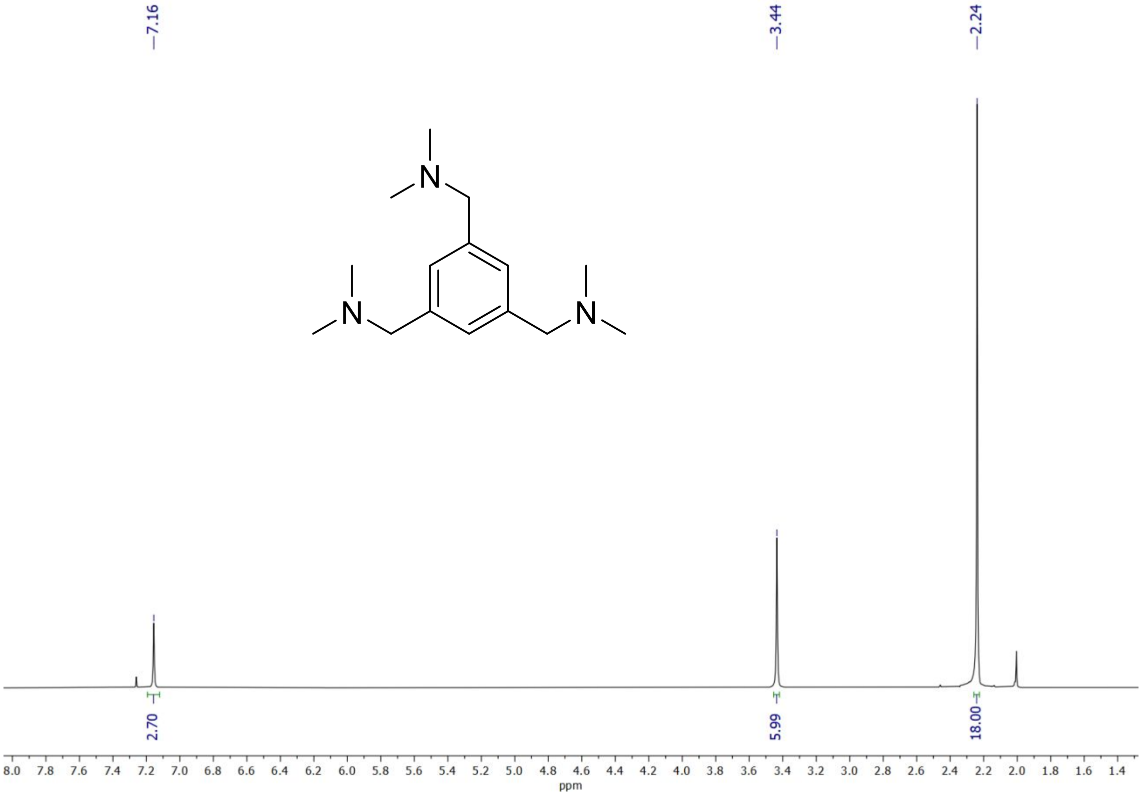


**Figure S48.** $^{1}H$-NMR ($CDCl_3$, 300MHz, 298K) spectrum for **15**.

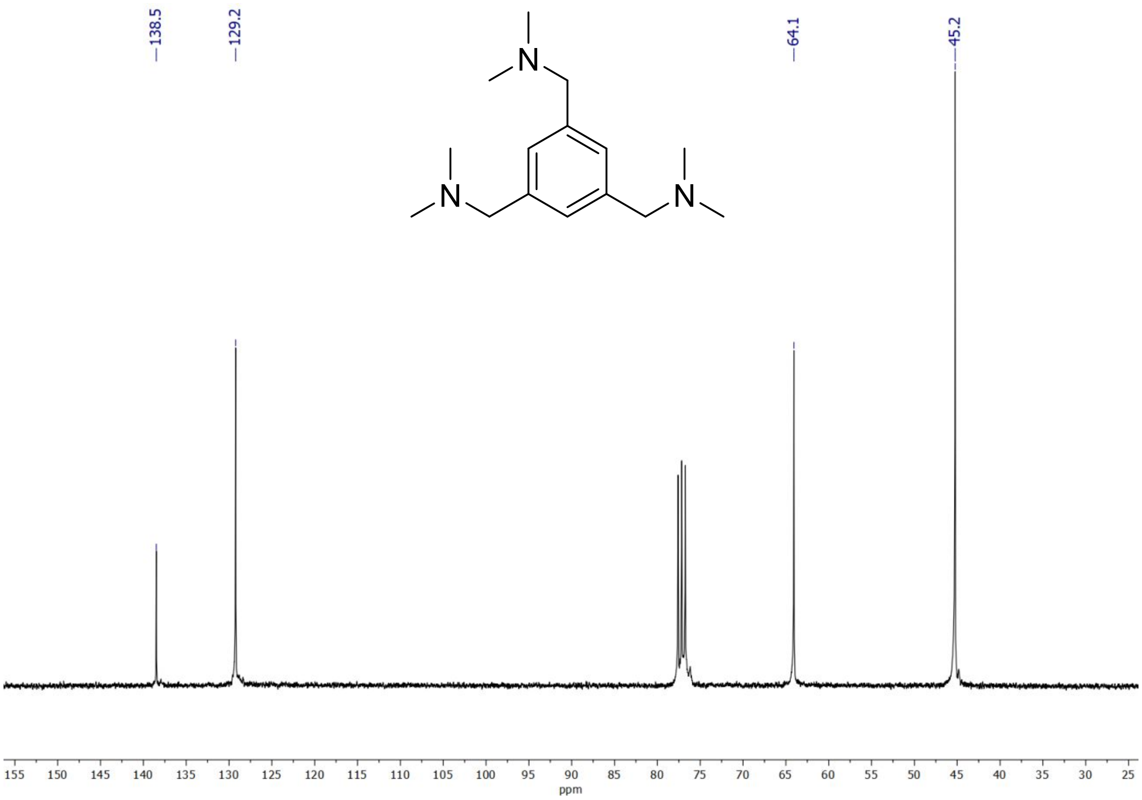


**Figure S49.** $^{13}C-\{^{1}H\}$-NMR ($CDCl_3$, 75 MHz, 298K) spectrum for **15**.

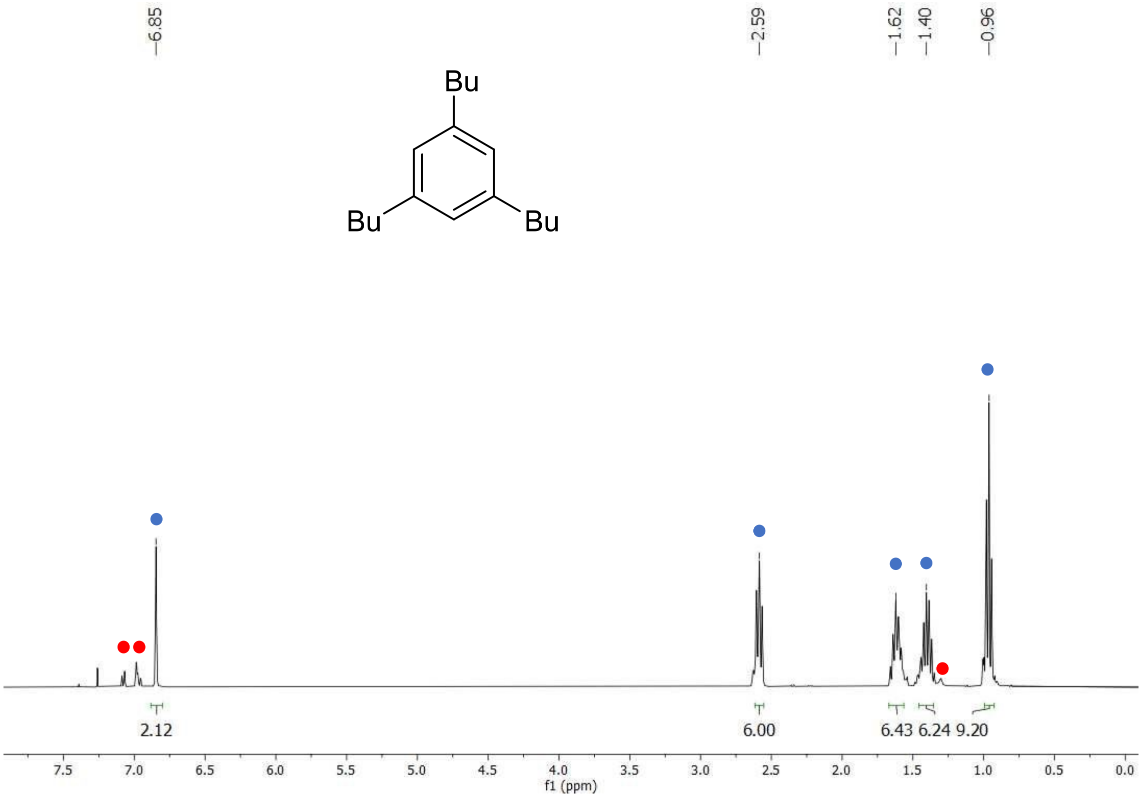


**Figure S50.** $^{1}H$-NMR ($CDCl_3$, 300MHz, 298K) spectrum for **16**. 1,3,5-isomer (●) and 1,2,4-isomer (●)

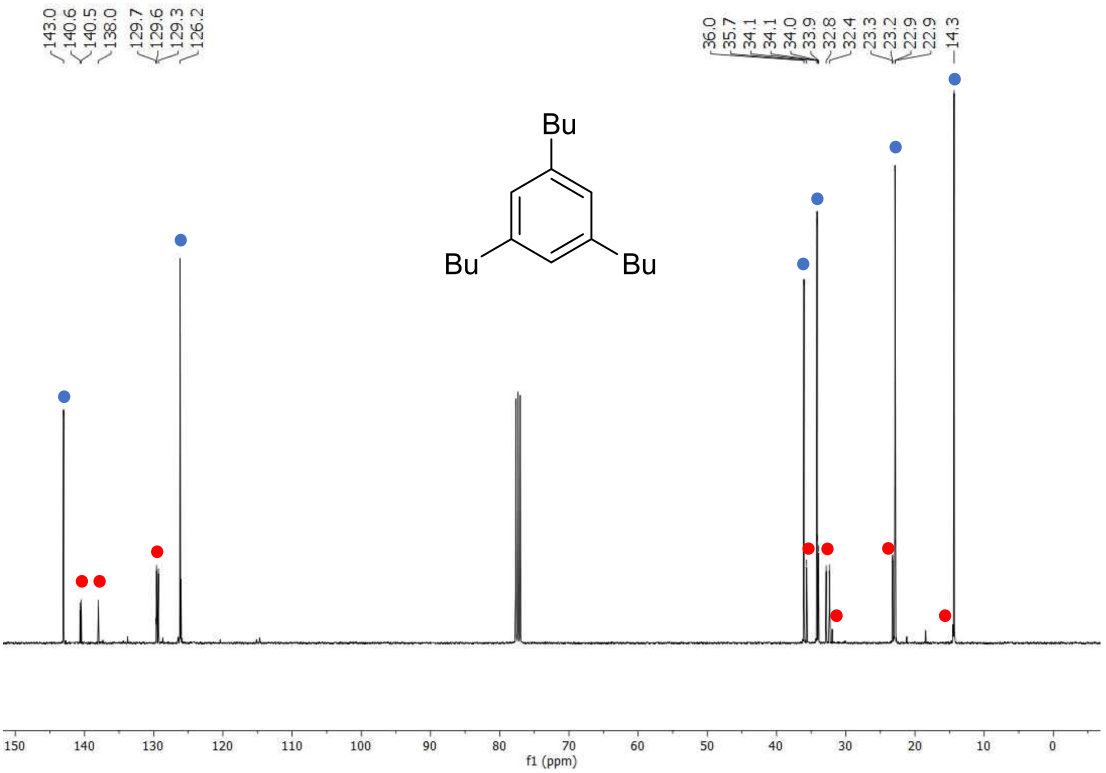


**Figure S51.** $^{13}C$-{$^{1}H$}-NMR ($CDCl_3$, 75 MHz, 298K) spectrum for **16**. 1,3,5-isomer (●) and 1,2,4-isomer (●)

## 11. Computational Details.

All the calculations reported in this paper were obtained with the Gaussian 16 suite of programs.[11] All species were optimized using the M06L functional[12] using the standard double-ζ quality def2-SVP3[13] basis sets for all atoms. This level is denoted M06L/def2-SVP. All stationary points were characterized by frequency calculations.[14] All species have positive definite Hessian matrices confirming that they are minima on the potential energy Surface.

Cartesian coordinates (in Å) and free energies (in a.u.) of all the stationary points discussed in the text. All calculations have been performed at the M06L/def2-SVP.

**A:** G= -2082.965978

| | | | |
|---|---|---|---|
| Ti | 0.150034000 | -0.994718000 | -0.703370000 |
| C | 0.001794000 | -3.011670000 | -0.430751000 |
| N | 1.515980000 | 0.316465000 | -0.310974000 |
| N | -1.168655000 | 0.432127000 | -0.475791000 |

| | | | |
|---|---|---|---|
| C | 1.053093000 | 4.012541000 | -0.658463000 |
| H | 1.649098000 | 4.926052000 | -0.717423000 |
| C | -0.336948000 | 4.074898000 | -0.696464000 |
| H | -0.843791000 | 5.038797000 | -0.782540000 |
| C | 0.954532000 | 1.595578000 | -0.435373000 |
| C | -0.471885000 | 1.654708000 | -0.506750000 |
| C | 1.694018000 | 2.776608000 | -0.540878000 |
| H | 2.785633000 | 2.717431000 | -0.513638000 |
| C | 4.958763000 | -0.944269000 | -0.698638000 |
| H | 5.526233000 | -1.548135000 | -1.414403000 |
| C | -1.092719000 | 2.900899000 | -0.635037000 |
| H | -2.185128000 | 2.938700000 | -0.676484000 |
| C | 5.598492000 | -0.456357000 | 0.445691000 |
| C | 4.834288000 | 0.280223000 | 1.356731000 |
| H | 5.303236000 | 0.629630000 | 2.283014000 |
| C | 3.612320000 | -0.681222000 | -0.952403000 |
| C | -4.842733000 | 0.003441000 | -0.769528000 |
| H | -5.586673000 | -0.226726000 | -1.539708000 |
| C | 2.923919000 | -1.226568000 | -2.167431000 |
| H | 2.448055000 | -0.430237000 | -2.762105000 |
| H | 2.140487000 | -1.971695000 | -1.910969000 |
| H | 3.616073000 | -1.765577000 | -2.827293000 |
| C | -3.088522000 | -0.020279000 | -2.577001000 |
| H | -3.959715000 | -0.063954000 | -3.243253000 |
| H | -2.502382000 | -0.936122000 | -2.765259000 |
| H | -2.455160000 | 0.819006000 | -2.901272000 |
| C | -1.483742000 | -3.040770000 | -0.707166000 |
| H | -1.792152000 | -3.746299000 | -1.492204000 |
| H | -1.829653000 | -2.036614000 | -1.090587000 |
| C | -2.158733000 | -3.236520000 | 0.653631000 |
| H | -3.189807000 | -2.850697000 | 0.684533000 |
| H | -2.213293000 | -4.316108000 | 0.865680000 |
| C | 2.877299000 | 0.106231000 | -0.041728000 |
| C | -3.501322000 | 0.125146000 | -1.145042000 |
| C | -2.542758000 | 0.422272000 | -0.155487000 |
| C | 3.482200000 | 0.565544000 | 1.149074000 |
| C | -2.944303000 | 0.662196000 | 1.177938000 |
| C | 2.681274000 | 1.283296000 | 2.186355000 |
| H | 1.674693000 | 0.848613000 | 2.280761000 |
| H | 2.528629000 | 2.346645000 | 1.942919000 |
| H | 3.171395000 | 1.239428000 | 3.167537000 |
| C | -5.262245000 | 0.184099000 | 0.551395000 |
| C | 7.053341000 | -0.709234000 | 0.688868000 |
| H | 7.417437000 | -1.582918000 | 0.132392000 |
| H | 7.268017000 | -0.874950000 | 1.753692000 |
| H | 7.670929000 | 0.147836000 | 0.375855000 |
| C | -1.939274000 | 1.063395000 | 2.209641000 |
| H | -0.993855000 | 0.511637000 | 2.092615000 |
| H | -2.316963000 | 0.902930000 | 3.228007000 |
| H | -1.676421000 | 2.130192000 | 2.119730000 |
| C | -4.295530000 | 0.527617000 | 1.503061000 |
| H | -4.603896000 | 0.696343000 | 2.540604000 |
| C | -1.223224000 | -2.538835000 | 1.654119000 |
| H | -1.662379000 | -1.592203000 | 2.012088000 |
| H | -1.074562000 | -3.150747000 | 2.560593000 |
| C | 0.077893000 | -2.345160000 | 0.887128000 |
| H | 1.024641000 | -2.372875000 | 1.446386000 |
| C | -6.696061000 | 0.006115000 | 0.942230000 |
| H | -7.369392000 | 0.076212000 | 0.077783000 |
| H | -7.017070000 | 0.754810000 | 1.679922000 |
| H | -6.869867000 | -0.979310000 | 1.403347000 |

| | | | |
|---|---|---|---|
| H | 0.646202000 | -3.838978000 | -0.755144000 |

**B:** G= -2083.563492

| | | | |
|---|---|---|---|
| Ti | -0.051261000 | 0.901690000 | -0.572565000 |
| C | 0.398628000 | 2.873646000 | -0.328347000 |
| N | 1.104783000 | -0.669932000 | -0.209155000 |
| N | -1.553777000 | -0.393376000 | -0.470997000 |
| C | 0.144613000 | -4.233191000 | -0.832177000 |
| H | 0.607448000 | -5.222321000 | -0.897957000 |
| C | -1.233363000 | -4.089025000 | -0.965964000 |
| H | -1.867792000 | -4.962016000 | -1.144865000 |
| C | 0.386050000 | -1.828671000 | -0.487982000 |
| C | -1.034947000 | -1.680643000 | -0.620815000 |
| C | 0.945523000 | -3.108980000 | -0.599279000 |
| H | 2.026905000 | -3.217721000 | -0.468376000 |
| C | 4.240543000 | -0.608664000 | 1.755528000 |
| H | 4.545883000 | -0.520939000 | 2.804941000 |
| C | -1.813207000 | -2.818575000 | -0.864055000 |
| H | -2.897369000 | -2.694364000 | -0.952139000 |
| C | 5.222558000 | -0.708642000 | 0.764865000 |
| C | 4.801248000 | -0.808905000 | -0.565876000 |
| H | 5.552831000 | -0.873735000 | -1.361930000 |
| C | 2.876494000 | -0.619959000 | 1.450778000 |
| C | -4.581301000 | -0.347271000 | 1.669988000 |
| H | -4.868517000 | -0.618528000 | 2.693431000 |
| C | 1.837065000 | -0.505281000 | 2.516813000 |
| H | 1.182546000 | -1.391096000 | 2.538565000 |
| H | 1.156912000 | 0.343291000 | 2.315945000 |
| H | 2.283670000 | -0.380793000 | 3.513261000 |
| C | -2.253562000 | -1.156353000 | 2.192807000 |
| H | -2.618450000 | -1.184025000 | 3.228991000 |
| H | -1.323310000 | -0.562604000 | 2.150674000 |
| H | -1.959312000 | -2.179452000 | 1.906857000 |
| C | 1.865462000 | 2.937761000 | 0.116100000 |
| H | 2.497148000 | 2.104405000 | -0.246841000 |
| H | 1.938269000 | 2.915739000 | 1.219601000 |
| C | 2.331594000 | 4.296894000 | -0.411812000 |
| H | 3.260017000 | 4.665680000 | 0.056421000 |
| H | 2.537104000 | 4.204272000 | -1.491801000 |
| C | 2.473683000 | -0.725771000 | 0.101342000 |
| C | -3.268677000 | -0.581286000 | 1.261603000 |
| C | -2.879145000 | -0.214423000 | -0.052757000 |
| C | 3.449134000 | -0.819014000 | -0.918942000 |
| C | -3.824682000 | 0.376873000 | -0.919324000 |
| C | 3.016372000 | -0.893341000 | -2.345101000 |
| H | 2.303158000 | -0.080988000 | -2.579474000 |
| H | 2.461976000 | -1.822545000 | -2.554439000 |
| H | 3.868129000 | -0.837822000 | -3.036981000 |
| C | -5.533689000 | 0.239829000 | 0.826237000 |
| C | 6.679514000 | -0.731607000 | 1.113916000 |
| H | 6.868781000 | -0.308339000 | 2.110585000 |
| H | 7.285407000 | -0.164274000 | 0.391600000 |
| H | 7.086502000 | -1.757055000 | 1.122267000 |
| C | -3.416175000 | 0.754225000 | -2.305603000 |
| H | -2.619917000 | 1.522137000 | -2.304767000 |
| H | -4.262330000 | 1.142890000 | -2.889910000 |
| H | -2.978439000 | -0.100751000 | -2.843298000 |
| C | -5.128145000 | 0.595183000 | -0.463006000 |
| H | -5.854296000 | 1.054041000 | -1.144662000 |
| C | 1.131466000 | 5.222903000 | -0.180484000 |
| H | 1.251567000 | 5.780746000 | 0.763829000 |

| | | | |
|---|---|---|---|
| H | 1.043161000 | 5.987435000 | -0.969325000 |
| C | -0.107819000 | 4.288354000 | -0.112479000 |
| H | -0.591533000 | 4.380603000 | 0.874551000 |
| H | -0.884096000 | 4.559080000 | -0.846330000 |
| C | -6.935014000 | 0.477246000 | 1.299897000 |
| H | -7.452556000 | -0.461509000 | 1.557985000 |
| H | -7.543046000 | 0.983627000 | 0.537192000 |
| H | -6.964575000 | 1.101359000 | 2.207705000 |
| H | 0.457492000 | 2.701688000 | -1.479023000 |

**C:** G= -2119.946324

| | | | |
|---|---|---|---|
| Ti | -0.006042000 | -1.128971000 | 0.036600000 |
| N | 1.279716000 | 0.479714000 | 0.259270000 |
| N | -1.280453000 | 0.487667000 | 0.277086000 |
| C | 0.721239000 | 1.739404000 | 0.297319000 |
| C | 1.407901000 | 2.959714000 | 0.350784000 |
| H | 2.503203000 | 2.949692000 | 0.347720000 |
| C | 0.708841000 | 4.174004000 | 0.404479000 |
| H | 1.264395000 | 5.116080000 | 0.442483000 |
| C | -0.684271000 | 4.178449000 | 0.412984000 |
| H | -1.233291000 | 5.124054000 | 0.457807000 |
| C | -1.391706000 | 2.968600000 | 0.367835000 |
| H | -2.487060000 | 2.965787000 | 0.378248000 |
| C | -0.713732000 | 1.743894000 | 0.306218000 |
| C | 2.664016000 | 0.320246000 | 0.128967000 |
| C | 3.427867000 | -0.125777000 | 1.229744000 |
| C | 4.798999000 | -0.344401000 | 1.070173000 |
| H | 5.383026000 | -0.687013000 | 1.933004000 |
| C | 5.446771000 | -0.133281000 | -0.151554000 |
| C | 4.671569000 | 0.296741000 | -1.234144000 |
| H | 5.151902000 | 0.449189000 | -2.208527000 |
| C | 3.297729000 | 0.524289000 | -1.119588000 |
| C | 2.471805000 | 0.937522000 | -2.293677000 |
| H | 1.588089000 | 0.283866000 | -2.390924000 |
| H | 3.044727000 | 0.908445000 | -3.230903000 |
| H | 2.067277000 | 1.955260000 | -2.167540000 |
| C | 6.925046000 | -0.335570000 | -0.290008000 |
| H | 7.489774000 | 0.594974000 | -0.108370000 |
| H | 7.201510000 | -0.674510000 | -1.299314000 |
| H | 7.307150000 | -1.077668000 | 0.425937000 |
| C | 2.751351000 | -0.354027000 | 2.543837000 |
| H | 2.234018000 | 0.554402000 | 2.889166000 |
| H | 3.463545000 | -0.666590000 | 3.320645000 |
| H | 1.966006000 | -1.124257000 | 2.464529000 |
| C | -2.664478000 | 0.333420000 | 0.140672000 |
| C | -3.432681000 | -0.121255000 | 1.234350000 |
| C | -4.804276000 | -0.335878000 | 1.068226000 |
| H | -5.393194000 | -0.680903000 | 1.926738000 |
| C | -5.447676000 | -0.111898000 | -0.152695000 |
| C | -4.669545000 | 0.335873000 | -1.226701000 |
| H | -5.148402000 | 0.508426000 | -2.198545000 |
| C | -3.296045000 | 0.558044000 | -1.106695000 |
| C | -2.468883000 | 0.990880000 | -2.272768000 |
| H | -2.074616000 | 2.011272000 | -2.135940000 |
| H | -3.037197000 | 0.964497000 | -3.212859000 |
| H | -1.578545000 | 0.346475000 | -2.371153000 |
| C | -6.914051000 | -0.371727000 | -0.319326000 |
| H | -7.411778000 | 0.421199000 | -0.898236000 |
| H | -7.428388000 | -0.446237000 | 0.649346000 |
| H | -7.109819000 | -1.314995000 | -0.856807000 |
| C | -2.763158000 | -0.353015000 | 2.551622000 |

| | | | |
|---|---|---|---|
| H | -1.976790000 | -1.122243000 | 2.475325000 |
| H | -3.479463000 | -0.669129000 | 3.323230000 |
| H | -2.249186000 | 0.555073000 | 2.902912000 |
| C | -1.218195000 | -3.023920000 | 0.295592000 |
| H | -2.149691000 | -3.139397000 | 0.856273000 |
| C | 0.049030000 | -3.037355000 | 1.009994000 |
| H | 0.088829000 | -3.366937000 | 2.049713000 |
| C | 1.261597000 | -2.997275000 | 0.209052000 |
| H | 2.232992000 | -3.081101000 | 0.704349000 |
| C | 1.210038000 | -2.616098000 | -1.140164000 |
| H | 2.141203000 | -2.407604000 | -1.674734000 |
| C | -0.057750000 | -2.272014000 | -1.761715000 |
| H | -0.097276000 | -2.001841000 | -2.818274000 |
| C | -1.269721000 | -2.647961000 | -1.054883000 |
| H | -2.240663000 | -2.460334000 | -1.522579000 |

**4:** G= -4007.927186

| | | | |
|---|---|---|---|
| Ti | -1.638881000 | -0.079902000 | 0.136378000 |
| Ti | 1.637414000 | -0.069489000 | -0.120131000 |
| N | 3.263206000 | -1.098623000 | 0.529275000 |
| N | 3.176009000 | 1.023290000 | -0.866462000 |
| N | -3.177021000 | 1.011948000 | 0.885205000 |
| N | -3.262506000 | -1.100752000 | -0.525049000 |
| C | 4.506665000 | -0.565529000 | 0.251356000 |
| C | 4.458001000 | 0.627980000 | -0.535620000 |
| C | 5.651547000 | 1.264235000 | -0.893578000 |
| H | 5.605658000 | 2.175644000 | -1.499147000 |
| C | 6.884890000 | 0.740978000 | -0.480829000 |
| H | 7.809899000 | 1.250849000 | -0.765109000 |
| C | 6.932249000 | -0.422419000 | 0.284641000 |
| H | 7.894843000 | -0.830575000 | 0.606039000 |
| C | 5.747151000 | -1.076393000 | 0.648848000 |
| H | 5.775800000 | -1.991294000 | 1.250208000 |
| C | 3.131460000 | -2.239100000 | 1.339160000 |
| C | 3.113473000 | -2.112599000 | 2.745884000 |
| C | 2.886779000 | -3.247446000 | 3.528444000 |
| H | 2.869681000 | -3.141278000 | 4.619522000 |
| C | 2.677382000 | -4.509842000 | 2.961314000 |
| C | 2.713717000 | -4.613878000 | 1.567457000 |
| H | 2.567212000 | -5.594935000 | 1.100795000 |
| C | 2.941771000 | -3.505178000 | 0.746511000 |
| C | 2.997035000 | -3.634982000 | -0.742227000 |
| H | 2.239484000 | -3.003142000 | -1.234290000 |
| H | 2.840428000 | -4.672408000 | -1.067463000 |
| H | 3.965782000 | -3.291777000 | -1.137731000 |
| C | 2.395302000 | -5.703367000 | 3.821528000 |
| H | 2.987184000 | -5.692839000 | 4.748428000 |
| H | 2.611383000 | -6.645439000 | 3.298566000 |
| H | 1.337199000 | -5.747201000 | 4.128666000 |
| C | 3.320682000 | -0.766423000 | 3.361770000 |
| H | 4.321840000 | -0.367147000 | 3.132796000 |
| H | 3.204212000 | -0.791915000 | 4.453527000 |
| H | 2.611790000 | -0.028337000 | 2.950956000 |
| C | 2.950174000 | 2.198336000 | -1.603141000 |
| C | 2.686553000 | 2.117973000 | -2.987043000 |
| C | 2.372959000 | 3.284620000 | -3.690749000 |
| H | 2.170813000 | 3.214361000 | -4.765944000 |
| C | 2.315826000 | 4.534189000 | -3.066399000 |
| C | 2.593847000 | 4.592867000 | -1.696147000 |
| H | 2.559030000 | 5.561687000 | -1.184128000 |
| C | 2.910227000 | 3.452262000 | -0.953856000 |

| | | | |
|---|---|---|---|
| C | 3.191830000 | 3.526026000 | 0.512010000 |
| H | 4.224468000 | 3.216971000 | 0.740982000 |
| H | 2.546609000 | 2.832132000 | 1.076115000 |
| H | 3.042116000 | 4.539309000 | 0.907923000 |
| C | 1.937305000 | 5.766318000 | -3.829699000 |
| H | 2.100107000 | 5.645532000 | -4.909793000 |
| H | 2.509136000 | 6.646924000 | -3.502243000 |
| H | 0.872513000 | 6.019595000 | -3.696321000 |
| C | 2.747957000 | 0.790719000 | -3.673452000 |
| H | 2.018052000 | 0.080623000 | -3.250758000 |
| H | 3.731286000 | 0.313625000 | -3.540379000 |
| H | 2.551827000 | 0.880130000 | -4.750581000 |
| C | -4.458328000 | 0.624430000 | 0.541856000 |
| C | -5.651649000 | 1.264460000 | 0.893390000 |
| H | -5.606190000 | 2.173620000 | 1.502313000 |
| C | -6.884278000 | 0.747892000 | 0.469970000 |
| H | -7.809126000 | 1.260843000 | 0.749189000 |
| C | -6.931048000 | -0.412667000 | -0.299606000 |
| H | -7.892997000 | -0.815913000 | -0.628976000 |
| C | -5.745949000 | -1.070069000 | -0.657980000 |
| H | -5.774051000 | -1.982398000 | -1.263195000 |
| C | -4.506363000 | -0.565156000 | -0.250797000 |
| C | -2.949885000 | 2.193626000 | 1.610536000 |
| C | -2.670580000 | 2.125380000 | 2.991795000 |
| C | -2.347841000 | 3.298159000 | 3.681602000 |
| H | -2.128174000 | 3.236508000 | 4.753835000 |
| C | -2.299874000 | 4.542502000 | 3.046482000 |
| C | -2.584736000 | 4.587853000 | 1.676970000 |
| H | -2.543931000 | 5.550413000 | 1.153651000 |
| C | -2.908774000 | 3.441079000 | 0.948127000 |
| C | -3.183809000 | 3.499646000 | -0.519654000 |
| H | -2.537635000 | 2.797524000 | -1.072869000 |
| H | -3.029109000 | 4.508167000 | -0.925767000 |
| H | -4.216122000 | 3.191224000 | -0.750878000 |
| C | -1.978849000 | 5.792572000 | 3.807010000 |
| H | -2.887432000 | 6.361298000 | 4.066058000 |
| H | -1.341306000 | 6.474925000 | 3.225999000 |
| H | -1.460126000 | 5.576121000 | 4.751180000 |
| C | -2.720991000 | 0.803532000 | 3.689674000 |
| H | -3.705693000 | 0.325154000 | 3.572865000 |
| H | -2.511254000 | 0.901885000 | 4.763486000 |
| H | -1.996758000 | 0.089512000 | 3.263662000 |
| C | -3.131840000 | -2.238283000 | -1.339387000 |
| C | -2.943291000 | -3.506450000 | -0.750900000 |
| C | -2.723485000 | -4.613994000 | -1.575453000 |
| H | -2.579290000 | -5.596795000 | -1.111755000 |
| C | -2.691255000 | -4.506309000 | -2.969016000 |
| C | -2.894606000 | -3.240954000 | -3.531951000 |
| H | -2.878506000 | -3.131633000 | -4.622733000 |
| C | -3.114600000 | -2.107267000 | -2.745676000 |
| C | -3.316504000 | -0.758022000 | -3.356796000 |
| H | -4.319362000 | -0.359390000 | -3.133936000 |
| H | -3.191767000 | -0.778446000 | -4.447754000 |
| H | -2.610596000 | -0.022122000 | -2.936726000 |
| C | -2.418855000 | -5.699335000 | -3.832994000 |
| H | -3.022533000 | -5.689706000 | -4.752328000 |
| H | -2.627361000 | -6.641521000 | -3.307193000 |
| H | -1.364747000 | -5.741985000 | -4.153656000 |
| C | -2.986111000 | -3.639082000 | 0.737845000 |
| H | -2.209129000 | -3.024833000 | 1.222222000 |
| H | -2.847016000 | -4.680284000 | 1.058906000 |

| | | | |
|---|---|---|---|
| H | -3.942516000 | -3.276272000 | 1.145397000 |
| C | 0.002605000 | -1.547074000 | -0.054689000 |
| H | -0.003666000 | -2.639759000 | -0.101860000 |
| C | 0.003703000 | -0.865714000 | 1.247868000 |
| H | 0.166044000 | -1.454386000 | 2.157433000 |
| C | 0.190928000 | 0.601563000 | 1.295558000 |
| H | 0.202046000 | 1.112249000 | 2.264090000 |
| C | 0.008756000 | 1.393011000 | 0.070902000 |
| H | -0.000356000 | 2.485751000 | 0.119852000 |
| C | -0.200133000 | 0.711384000 | -1.214393000 |
| H | -0.210943000 | 1.302339000 | -2.136004000 |
| C | -0.008268000 | -0.754545000 | -1.293730000 |
| H | -0.167525000 | -1.264718000 | -2.249833000 |

**4(1,3,5):** G= -5233.076685

| | | | |
|---|---|---|---|
| Ti | -1.644851000 | 0.023368000 | -0.055405000 |
| Ti | 1.678031000 | 0.130251000 | 0.178924000 |
| N | 3.213857000 | -1.104337000 | 0.790449000 |
| N | 3.323713000 | 1.005716000 | -0.741988000 |
| N | -3.408420000 | 0.807266000 | 0.742668000 |
| N | -3.056137000 | -1.298218000 | -0.776223000 |
| C | 4.424434000 | -0.919806000 | 0.138546000 |
| C | 4.472578000 | 0.221168000 | -0.716576000 |
| C | 5.647317000 | 0.487330000 | -1.435687000 |
| H | 5.680359000 | 1.358867000 | -2.095649000 |
| C | 6.769514000 | -0.335665000 | -1.312366000 |
| H | 7.671398000 | -0.111520000 | -1.888782000 |
| C | 6.736053000 | -1.428860000 | -0.449549000 |
| H | 7.615611000 | -2.067774000 | -0.330228000 |
| C | 5.574827000 | -1.714651000 | 0.274702000 |
| H | 5.562108000 | -2.562156000 | 0.965513000 |
| C | 3.189016000 | -2.100790000 | 1.786582000 |
| C | 3.283751000 | -1.719875000 | 3.142043000 |
| C | 3.367715000 | -2.707867000 | 4.128714000 |
| H | 3.445516000 | -2.400028000 | 5.178286000 |
| C | 3.378733000 | -4.070400000 | 3.813985000 |
| C | 3.273332000 | -4.426970000 | 2.466202000 |
| H | 3.258200000 | -5.488629000 | 2.193573000 |
| C | 3.156892000 | -3.473306000 | 1.451825000 |
| C | 3.015605000 | -3.885554000 | 0.024667000 |
| H | 2.197208000 | -3.333039000 | -0.461839000 |
| H | 2.816530000 | -4.961463000 | -0.073969000 |
| H | 3.921861000 | -3.652653000 | -0.559747000 |
| C | 3.431083000 | -5.113455000 | 4.886844000 |
| H | 3.919948000 | -4.742442000 | 5.798725000 |
| H | 3.969258000 | -6.014750000 | 4.560133000 |
| H | 2.420195000 | -5.442336000 | 5.181734000 |
| C | 3.356396000 | -0.268267000 | 3.494747000 |
| H | 4.252405000 | 0.201548000 | 3.058971000 |
| H | 3.384340000 | -0.113914000 | 4.582438000 |
| H | 2.496258000 | 0.296038000 | 3.093777000 |
| C | 3.574625000 | 2.335485000 | -1.156251000 |
| C | 3.229285000 | 2.814511000 | -2.435775000 |
| C | 3.603054000 | 4.112647000 | -2.809918000 |
| H | 3.329121000 | 4.466900000 | -3.810874000 |
| C | 4.322004000 | 4.956131000 | -1.963783000 |
| C | 4.653028000 | 4.467601000 | -0.695068000 |
| H | 5.201001000 | 5.114003000 | 0.000581000 |
| C | 4.288894000 | 3.189425000 | -0.275750000 |
| C | 4.623446000 | 2.716808000 | 1.101374000 |
| H | 5.364271000 | 1.900471000 | 1.088801000 |

| | | | |
|---|---|---|---|
| H | 3.734357000 | 2.298690000 | 1.603012000 |
| H | 5.023342000 | 3.529363000 | 1.723406000 |
| C | 4.679704000 | 6.351424000 | -2.372065000 |
| H | 4.678200000 | 6.472518000 | -3.464304000 |
| H | 5.672845000 | 6.647098000 | -2.003235000 |
| H | 3.965546000 | 7.088795000 | -1.969016000 |
| C | 2.523792000 | 1.945848000 | -3.424161000 |
| H | 1.987046000 | 1.137305000 | -2.918992000 |
| H | 3.230101000 | 1.466743000 | -4.123590000 |
| H | 1.810023000 | 2.516692000 | -4.037209000 |
| C | -4.514237000 | -0.028607000 | 0.603792000 |
| C | -5.773171000 | 0.192001000 | 1.183314000 |
| H | -5.919974000 | 1.071765000 | 1.816572000 |
| C | -6.841581000 | -0.678340000 | 0.945750000 |
| H | -7.811497000 | -0.484706000 | 1.412506000 |
| C | -6.666699000 | -1.776539000 | 0.108793000 |
| H | -7.499480000 | -2.454751000 | -0.098098000 |
| C | -5.421927000 | -2.014355000 | -0.481761000 |
| H | -5.299570000 | -2.864460000 | -1.158414000 |
| C | -4.329183000 | -1.166100000 | -0.235786000 |
| C | -3.755971000 | 2.127607000 | 1.115860000 |
| C | -3.648916000 | 2.583331000 | 2.446407000 |
| C | -4.075305000 | 3.873718000 | 2.772190000 |
| H | -3.977652000 | 4.211300000 | 3.810871000 |
| C | -4.601281000 | 4.747749000 | 1.818454000 |
| C | -4.723515000 | 4.275076000 | 0.509134000 |
| H | -5.151641000 | 4.930641000 | -0.258491000 |
| C | -4.337254000 | 2.982299000 | 0.146291000 |
| C | -4.586104000 | 2.481901000 | -1.239412000 |
| H | -3.768192000 | 1.834259000 | -1.590240000 |
| H | -4.721816000 | 3.304709000 | -1.954974000 |
| H | -5.489286000 | 1.849303000 | -1.274054000 |
| C | -4.984855000 | 6.149535000 | 2.179900000 |
| H | -5.750265000 | 6.554260000 | 1.502777000 |
| H | -4.123082000 | 6.836299000 | 2.127450000 |
| H | -5.374777000 | 6.217906000 | 3.205763000 |
| C | -3.111398000 | 1.684817000 | 3.511796000 |
| H | -3.850114000 | 0.921541000 | 3.807121000 |
| H | -2.838556000 | 2.245295000 | 4.417285000 |
| H | -2.227828000 | 1.130405000 | 3.162877000 |
| C | -2.944967000 | -2.276225000 | -1.788635000 |
| C | -2.818449000 | -3.647239000 | -1.472955000 |
| C | -2.875464000 | -4.594922000 | -2.497474000 |
| H | -2.789716000 | -5.656430000 | -2.237428000 |
| C | -3.024286000 | -4.231210000 | -3.839219000 |
| C | -3.117196000 | -2.868253000 | -4.135765000 |
| H | -3.235314000 | -2.554554000 | -5.179855000 |
| C | -3.090673000 | -1.888115000 | -3.137340000 |
| C | -3.288019000 | -0.444642000 | -3.473736000 |
| H | -4.237905000 | -0.070050000 | -3.059458000 |
| H | -3.303515000 | -0.277065000 | -4.559660000 |
| H | -2.497885000 | 0.191908000 | -3.039574000 |
| C | -3.023170000 | -5.264307000 | -4.922721000 |
| H | -3.517502000 | -4.903781000 | -5.835699000 |
| H | -3.528623000 | -6.189471000 | -4.610092000 |
| H | -1.997187000 | -5.550517000 | -5.209205000 |
| C | -2.666073000 | -4.070982000 | -0.050647000 |
| H | -1.879840000 | -3.488747000 | 0.452600000 |
| H | -2.417932000 | -5.137757000 | 0.035511000 |
| H | -3.588720000 | -3.890374000 | 0.526421000 |
| C | 0.082642000 | -1.352849000 | 0.016187000 |

| | | | |
|---|---|---|---|
| H | 0.120427000 | -2.444044000 | -0.024263000 |
| C | -0.130465000 | -0.704762000 | 1.321838000 |
| C | -0.002361000 | 0.770762000 | 1.366908000 |
| H | -0.124574000 | 1.265916000 | 2.335805000 |
| C | -0.006687000 | 1.603718000 | 0.144169000 |
| C | -0.006874000 | 0.879859000 | -1.151761000 |
| H | 0.079341000 | 1.473163000 | -2.067998000 |
| C | 0.242135000 | -0.588218000 | -1.237502000 |
| Si | 0.831946000 | -1.504412000 | -2.792974000 |
| Si | -0.690180000 | -1.719587000 | 2.816843000 |
| Si | -0.201914000 | 3.484749000 | 0.227077000 |
| C | 2.711628000 | -1.571180000 | -2.907104000 |
| H | 3.159787000 | -1.959115000 | -1.980272000 |
| H | 2.981639000 | -2.272729000 | -3.713890000 |
| H | 3.211382000 | -0.618748000 | -3.116450000 |
| C | 0.304477000 | -3.311403000 | -2.754199000 |
| H | 0.215746000 | -3.738551000 | -1.744362000 |
| H | -0.662596000 | -3.458739000 | -3.250403000 |
| H | 1.050825000 | -3.918244000 | -3.290564000 |
| C | 0.150575000 | -0.691799000 | -4.351503000 |
| H | 0.771896000 | -0.963093000 | -5.219502000 |
| H | -0.875040000 | -1.029487000 | -4.559287000 |
| H | 0.133696000 | 0.406488000 | -4.297722000 |
| C | -0.127394000 | -0.903848000 | 4.417662000 |
| H | -0.601383000 | -1.405136000 | 5.276184000 |
| H | 0.960833000 | -0.970103000 | 4.556597000 |
| H | -0.410164000 | 0.158644000 | 4.473661000 |
| C | -0.041352000 | -3.480847000 | 2.733186000 |
| H | 0.939801000 | -3.570332000 | 3.216772000 |
| H | -0.737126000 | -4.158400000 | 3.252606000 |
| H | 0.071216000 | -3.861751000 | 1.707004000 |
| C | -2.568144000 | -1.831719000 | 2.850469000 |
| H | -2.978619000 | -2.186880000 | 1.894253000 |
| H | -2.886557000 | -2.541972000 | 3.631225000 |
| H | -3.056570000 | -0.871251000 | 3.051672000 |
| C | 1.331031000 | 4.545057000 | 0.019966000 |
| H | 2.089508000 | 4.342765000 | 0.789013000 |
| H | 1.811296000 | 4.474673000 | -0.963612000 |
| H | 1.007209000 | 5.591162000 | 0.156121000 |
| C | -1.332284000 | 3.971308000 | -1.199164000 |
| H | -0.741103000 | 4.034831000 | -2.127568000 |
| H | -2.137944000 | 3.246747000 | -1.373429000 |
| H | -1.795214000 | 4.957142000 | -1.039632000 |
| C | -0.802666000 | 3.984391000 | 1.944016000 |
| H | -1.617104000 | 4.721271000 | 1.881450000 |
| H | -1.174403000 | 3.157976000 | 2.559730000 |
| H | 0.027775000 | 4.455951000 | 2.491742000 |

**4(1,2,4):** G= -5233.065105

| | | | |
|---|---|---|---|
| Ti | 1.642482000 | -0.105371000 | -0.149453000 |
| Ti | -1.669127000 | -0.009818000 | -0.035954000 |
| N | -3.236902000 | -1.319679000 | -0.265833000 |
| N | -3.300664000 | 1.227700000 | 0.343799000 |
| N | 3.218580000 | -0.245509000 | -1.511771000 |
| N | 3.269290000 | -0.251168000 | 1.106047000 |
| C | -4.467305000 | -0.850455000 | 0.174955000 |
| C | -4.493868000 | 0.531885000 | 0.523046000 |
| C | -5.691486000 | 1.094024000 | 0.988747000 |
| H | -5.711012000 | 2.155185000 | 1.254416000 |
| C | -6.855674000 | 0.326515000 | 1.094828000 |
| H | -7.776565000 | 0.787605000 | 1.463008000 |

| | | | |
|---|---|---|---|
| C | -6.838337000 | -1.015348000 | 0.722966000 |
| H | -7.747331000 | -1.620222000 | 0.785266000 |
| C | -5.653068000 | -1.597069000 | 0.260967000 |
| H | -5.646593000 | -2.646046000 | -0.049038000 |
| C | -3.239959000 | -2.603193000 | -0.851055000 |
| C | -3.348625000 | -2.724773000 | -2.253627000 |
| C | -3.414661000 | -3.998120000 | -2.828500000 |
| H | -3.498618000 | -4.080606000 | -3.918203000 |
| C | -3.373364000 | -5.163992000 | -2.058064000 |
| C | -3.274938000 | -5.023228000 | -0.670810000 |
| H | -3.248673000 | -5.920395000 | -0.040923000 |
| C | -3.212773000 | -3.769646000 | -0.055918000 |
| C | -3.157286000 | -3.643872000 | 1.429107000 |
| H | -2.298002000 | -3.029915000 | 1.737489000 |
| H | -3.081304000 | -4.622020000 | 1.924306000 |
| H | -4.051396000 | -3.132047000 | 1.821587000 |
| C | -3.375104000 | -6.516934000 | -2.698334000 |
| H | -3.881673000 | -6.511048000 | -3.673757000 |
| H | -3.868135000 | -7.270982000 | -2.068117000 |
| H | -2.348724000 | -6.879764000 | -2.877326000 |
| C | -3.423632000 | -1.495084000 | -3.099564000 |
| H | -4.299673000 | -0.882548000 | -2.834690000 |
| H | -3.487452000 | -1.739134000 | -4.168659000 |
| H | -2.546562000 | -0.842700000 | -2.949180000 |
| C | -3.472940000 | 2.616381000 | 0.163575000 |
| C | -3.124328000 | 3.544708000 | 1.165318000 |
| C | -3.379670000 | 4.906378000 | 0.961793000 |
| H | -3.104413000 | 5.615048000 | 1.752204000 |
| C | -3.999850000 | 5.383655000 | -0.195071000 |
| C | -4.351310000 | 4.447292000 | -1.173549000 |
| H | -4.826540000 | 4.793879000 | -2.098773000 |
| C | -4.088344000 | 3.085159000 | -1.025213000 |
| C | -4.434068000 | 2.119685000 | -2.110158000 |
| H | -5.302948000 | 1.494667000 | -1.844748000 |
| H | -3.605954000 | 1.412525000 | -2.280310000 |
| H | -4.661211000 | 2.633291000 | -3.054487000 |
| C | -4.242268000 | 6.846546000 | -0.403136000 |
| H | -4.264911000 | 7.398289000 | 0.547008000 |
| H | -5.193099000 | 7.035344000 | -0.922635000 |
| H | -3.453538000 | 7.309168000 | -1.019771000 |
| C | -2.561758000 | 3.066823000 | 2.464090000 |
| H | -1.735825000 | 2.357391000 | 2.312617000 |
| H | -3.323105000 | 2.522733000 | 3.047223000 |
| H | -2.196993000 | 3.897043000 | 3.085202000 |
| C | 4.393159000 | -0.712502000 | -0.929812000 |
| C | 5.506009000 | -1.180946000 | -1.638252000 |
| H | 5.457642000 | -1.210241000 | -2.731203000 |
| C | 6.672286000 | -1.569014000 | -0.971822000 |
| H | 7.531017000 | -1.928830000 | -1.545475000 |
| C | 6.738598000 | -1.481086000 | 0.417502000 |
| H | 7.653630000 | -1.761714000 | 0.946788000 |
| C | 5.629720000 | -1.037308000 | 1.146010000 |
| H | 5.684890000 | -0.958362000 | 2.235958000 |
| C | 4.437955000 | -0.680522000 | 0.496746000 |
| C | 3.345698000 | 0.329591000 | -2.792081000 |
| C | 2.612412000 | -0.147421000 | -3.903637000 |
| C | 2.700189000 | 0.519390000 | -5.133419000 |
| H | 2.118620000 | 0.130697000 | -5.978051000 |
| C | 3.519393000 | 1.629009000 | -5.327491000 |
| C | 4.283877000 | 2.055726000 | -4.235460000 |
| H | 4.944105000 | 2.923326000 | -4.353239000 |

| | | | |
|---|---|---|---|
| C | 4.220865000 | 1.438807000 | -2.987383000 |
| C | 5.055180000 | 1.982417000 | -1.870639000 |
| H | 4.558612000 | 1.854889000 | -0.899114000 |
| H | 5.267053000 | 3.051149000 | -2.020060000 |
| H | 6.019878000 | 1.457257000 | -1.781352000 |
| C | 3.575247000 | 2.346434000 | -6.640588000 |
| H | 4.606925000 | 2.596980000 | -6.930107000 |
| H | 3.021961000 | 3.299788000 | -6.612625000 |
| H | 3.137454000 | 1.748560000 | -7.452050000 |
| C | 1.799961000 | -1.400894000 | -3.829513000 |
| H | 2.096783000 | -2.107298000 | -4.620552000 |
| H | 0.722611000 | -1.216801000 | -3.975210000 |
| H | 1.924609000 | -1.898884000 | -2.860852000 |
| C | 3.322524000 | 0.074612000 | 2.476618000 |
| C | 3.255749000 | -0.921477000 | 3.475269000 |
| C | 3.319911000 | -0.551875000 | 4.821789000 |
| H | 3.256398000 | -1.336153000 | 5.585508000 |
| C | 3.450748000 | 0.781621000 | 5.219843000 |
| C | 3.530041000 | 1.753789000 | 4.218056000 |
| H | 3.640727000 | 2.806798000 | 4.501349000 |
| C | 3.470176000 | 1.426066000 | 2.860190000 |
| C | 3.590846000 | 2.479951000 | 1.809155000 |
| H | 4.527393000 | 2.369083000 | 1.239083000 |
| H | 3.570571000 | 3.492835000 | 2.234262000 |
| H | 2.779851000 | 2.400097000 | 1.066504000 |
| C | 3.447076000 | 1.160636000 | 6.667884000 |
| H | 3.984526000 | 2.102318000 | 6.848262000 |
| H | 3.905399000 | 0.385746000 | 7.298883000 |
| H | 2.420483000 | 1.306247000 | 7.044740000 |
| C | 3.135740000 | -2.352329000 | 3.072865000 |
| H | 2.281656000 | -2.492356000 | 2.392611000 |
| H | 3.009212000 | -3.017897000 | 3.937902000 |
| H | 4.023359000 | -2.690721000 | 2.513432000 |
| C | -0.090427000 | -0.572841000 | 1.304144000 |
| C | 0.084035000 | -1.528406000 | 0.140565000 |
| C | -0.112152000 | -0.947040000 | -1.220242000 |
| H | -0.190601000 | -1.621846000 | -2.073130000 |
| C | -0.089106000 | 0.492629000 | -1.467881000 |
| C | 0.096982000 | 1.442712000 | -0.358354000 |
| C | 0.114226000 | 0.881476000 | 1.004864000 |
| H | 0.200162000 | 1.578843000 | 1.842359000 |
| Si | 0.655055000 | -3.347221000 | -0.003232000 |
| H | -0.063675000 | 0.852188000 | -2.504585000 |
| C | -0.026567000 | -4.102068000 | -1.590045000 |
| H | 0.075934000 | -5.197916000 | -1.535039000 |
| H | -1.080613000 | -3.881715000 | -1.789150000 |
| H | 0.557626000 | -3.772958000 | -2.463142000 |
| C | 0.304748000 | -4.536010000 | 1.412967000 |
| H | 0.684511000 | -5.517087000 | 1.084576000 |
| H | 0.841721000 | -4.280962000 | 2.336343000 |
| H | -0.757640000 | -4.664563000 | 1.655515000 |
| C | 2.516590000 | -3.433444000 | -0.295941000 |
| H | 3.157958000 | -3.039825000 | 0.502015000 |
| H | 2.788183000 | -4.492295000 | -0.443927000 |
| H | 2.800747000 | -2.902208000 | -1.216804000 |
| Si | -0.659511000 | -0.811070000 | 3.108627000 |
| Si | 0.366942000 | 3.283005000 | -0.712173000 |
| C | -0.307209000 | -2.434186000 | 3.992703000 |
| H | 0.756219000 | -2.686844000 | 4.088046000 |
| H | -0.706125000 | -2.312268000 | 5.012880000 |
| H | -0.828455000 | -3.293143000 | 3.549839000 |

| | | | |
|---|---|---|---|
| C | 0.048731000 | 0.582075000 | 4.162765000 |
| H | -0.513760000 | 1.518527000 | 4.026404000 |
| H | -0.031959000 | 0.312734000 | 5.228213000 |
| H | 1.106031000 | 0.792735000 | 3.958831000 |
| C | -2.526968000 | -0.573311000 | 3.218092000 |
| H | -3.127500000 | -1.309388000 | 2.668263000 |
| H | -2.830957000 | -0.625292000 | 4.277302000 |
| H | -2.825427000 | 0.414456000 | 2.841516000 |
| C | 0.402981000 | 4.256661000 | 0.898982000 |
| H | -0.611956000 | 4.531157000 | 1.219069000 |
| H | 0.885291000 | 3.717774000 | 1.728455000 |
| H | 0.965842000 | 5.192099000 | 0.754540000 |
| C | 2.000626000 | 3.482535000 | -1.612459000 |
| H | 2.179756000 | 4.543931000 | -1.848866000 |
| H | 2.864870000 | 3.120671000 | -1.039949000 |
| H | 1.990777000 | 2.933649000 | -2.567263000 |
| C | -0.926968000 | 3.984778000 | -1.878092000 |
| H | -1.299170000 | 3.232185000 | -2.590057000 |
| H | -1.794480000 | 4.387780000 | -1.341310000 |
| H | -0.488750000 | 4.803651000 | -2.469635000 |

## References


[1] a) Evans, D. F., The determination of the paramagnetic susceptibility of substances in solution by nuclear magnetic resonance, *J. Chem. Soc.* **1959**, 2003-2005. b) Sur, S. K.; *J. Magn. Reason.* **1989**, 169-173. c) Bain, G. A.; Berry, J. F., Diamagnetic Corrections and Pascal's Constants, *J. Chem. Educ.* **2008**, *85*, 532-536.

[2] Stoll, S.; Schweiger, A. EasySpin, a comprehensive software package for spectral simulation and analysis in EPR, *Journal of Magnetic Resonance* **2006**, *178*, 42-55.

[3] Sen, A; Sato, T; Ohno, A; Baek, H; Muranaka, A; Yamada Y.M.A; Polymer-Supported-Cobalt-Catalyzed Regioselective Cyclotrimerization of Aryl Alkynes, *JACS Au.* **2021**, *1*, 2080-2087.

[4] Liu, Y; Yan, X; Yang, N; Xi, C; Highly regioselective cyclotrimerization of terminal alkynes catalyzed by Fe(II) complexes bearing 2-(benzimidazolyl)-6-(1-(arylimino)ethyl)pyridines, *Catal. Commun.***2011**, *12*, 489-492.

[5] Drenna, D; Villa, M; Gieshoff, T.N.; Fischer, F; Hapke, M; Jacobi von Wangelin, A; Iron-Catalyzed Cyclotrimerization of Terminal Alkynes by Dual Catalyst Activation in the Absence of Reductants*, Angew. Chem. Int. Ed.* **2017**, *56*, 8451- 8454.

[6] Wagh G.D.; Akamanchi K.G.; Sulfated tungstate catalyzed synthesis of C3-symmetric 1,3,5-triaryl benzenes under solvent-free condition, *Tetrahedron Lett.* **2017**,*58*, 3032-3036.

[7] Xi, C; Supa, Z; Liua, Y; Cyclotrimerization of terminal alkynes catalyzed by the system of $NiCl_2$/Zn and (benzimidazolyl)-6-(1-(arylimino)ethyl)pyridines, *Dalton Trans*. **2013**,*42*, 13327-13330.

[8] Tagliatesta, P; Elakkari, E; Leoni, A; Lemboa, A; Ciceroa, D; Highly selective biaryl formation by the cyclooligomerization of arylethynes catalyzed by rhodium and ruthenium porphyrins, *New J. Chem.*, **2008**, *32*, 1847-1849.

[9] Bu, X; Zhang, Z; Zhou, X; Switching from Dimerization to Cyclotrimerization Selectivity by $FeCl_3$ in the $Y[N(TMS)_2]_3$-Catalyzed Transformation of Terminal Alkynes: A New Strategy for Controlling the Selectivity of Organolanthanide-Based Catalysis, *Organometallics* **2010**, *29,* 3530-3534.

[10] Jeong, J; Fujita, K; Selective Synthesis of Bisdimethylamine Derivatives from Diols and an Aqueous Solution of Dimethylamine through Iridium-Catalyzed Borrowing Hydrogen Pathway, *ChemCatChem* **2022**, *14*, e202101499.

[11] Gaussian 16, Revision B.01, Frisch, M. J.; Trucks, G. W.; Schlegel, H. B.; Scuseria, G. E.; Robb, M. A.; Cheeseman, J. R.; Scalmani, G.; Barone, V.; Petersson, G. A.; Nakatsuji, H.; Li, X.; Caricato, M.; Marenich, A. V.; Bloino, J.; Janesko, B. G.; Gomperts, R.; Mennucci, B.; Hratchian, H. P.; Ortiz, J. V.; Izmaylov, A. F.; Sonnenberg, J. L.; Williams-Young, D.; Ding, F.; Lipparini, F.; Egidi, F.; Goings, J.; Peng, B.; Petrone, A.; Henderson, T.; Ranasinghe, D.; Zakrzewski, V. G.; Gao, J.; Rega, N.; Zheng, G.; Liang, W.; Hada, M.; Ehara, M.; Toyota, K.; Fukuda, R.; Hasegawa, J.; Ishida, M.; Nakajima, T.; Honda, Y.; Kitao, O.; Nakai, H.; Vreven, T.; Throssell, K.; Montgomery, J. A., Jr.; Peralta, J. E.; Ogliaro, F.; Bearpark, M. J.; Heyd, J. J.; Brothers, E. N.; Kudin, K. N.; Staroverov, V. N.; Keith, T. A.; Kobayashi, R.; Normand, J.; Raghavachari, K.; Rendell, A. P.; Burant, J. C.; Iyengar, S. S.; Tomasi, J.; Cossi, M.; Millam, J. M.; Klene, M.; Adamo, C.; Cammi, R.; Ochterski, J. W.; Martin, R. L.; Morokuma, K.; Farkas, O.; Foresman, J. B.; Fox, D. J. Gaussian, Inc., Wallingford CT, 2016

[12] Zhao, Y.; Truhlar, D. G. The M06 suite of density functionals for main group thermochemistry, thermochemical kinetics, noncovalent interactions, excited states, and transition elements: two new functionals and systematic testing of four M06-class functionals and 12 other functionals, *Theor. Chem. Acc.* **2008**, *120*, 215-241.

[13] Weigend, F.; Ahlrichs, R. Balanced basis sets of split valence, triple zeta valence and quadruple zeta valence quality for H to Rn: Design and assessment of accuracy, *Phys. Chem. Chem. Phys*. **2005**, *7*, 3297-3305.

[14] McIver, J. W.; Komornicki, A. K. Structure of transition states in organic reactions. General theory and an application to the cyclobutene-butadiene isomerization using a semiempirical molecular orbital method, *J. Am. Chem. Soc.* **1972**, *94*, 2625-2633.
